# Molecular and cellular mechanisms underlying the pathogenesis of Alzheimer’s disease

**DOI:** 10.1186/s13024-020-00391-7

**Published:** 2020-07-16

**Authors:** Tiantian Guo, Denghong Zhang, Yuzhe Zeng, Timothy Y. Huang, Huaxi Xu, Yingjun Zhao

**Affiliations:** 1 Fujian Provincial Key Laboratory of Neurodegenerative Disease and Aging Research, Institute of Neuroscience, School of Medicine, Xiamen University, Xiamen, China; 2 Department of Orthopaedics, Orthopaedic Center of People’s Liberation Army, The Affiliated Southeast Hospital of Xiamen University, Zhangzhou, China; 3 Neuroscience Initiative, Sanford Burnham Prebys Medical Discovery Institute, La Jolla, California USA

**Keywords:** Alzheimer’s disease, Aβ, Tau, Microglia, Astrocyte

## Abstract

Alzheimer’s disease (AD) is the most common neurodegenerative disorder seen in age-dependent dementia. There is currently no effective treatment for AD, which may be attributed in part to lack of a clear underlying mechanism. Studies within the last few decades provide growing evidence for a central role of amyloid β (Aβ) and tau, as well as glial contributions to various molecular and cellular pathways in AD pathogenesis. Herein, we review recent progress with respect to Aβ- and tau-associated mechanisms, and discuss glial dysfunction in AD with emphasis on neuronal and glial receptors that mediate Aβ-induced toxicity. We also discuss other critical factors that may affect AD pathogenesis, including genetics, aging, variables related to environment, lifestyle habits, and describe the potential role of apolipoprotein E (APOE), viral and bacterial infection, sleep, and microbiota. Although we have gained much towards understanding various aspects underlying this devastating neurodegenerative disorder, greater commitment towards research in molecular mechanism, diagnostics and treatment will be needed in future AD research.

## Introduction

As the most prominent form of dementia, Alzheimer’s disease (AD) is becoming a dire global health concern among the elderly [1]. According to current statistics (2019), nearly 50 million people suffer from AD or AD-related dementia worldwide [2]. Alzheimer’s and age-related dementia are leading causes of disability in aged individuals, where the risk of AD onset increases exponentially with increased age. The prevalence of dementia is predicted to increase by 68% in low- and middle-income countries by 2050 [3]. Clinical symptoms of AD include progressive memory decline, impaired executive function and difficulties executing routine daily activity; early symptoms of AD onset include changes in thinking or unconscious behavior, memory impairment with respect to new information, and dysfunctional changes in language and speech [4]. In addition, 20 to 30% of early AD patients show significant depressive symptoms and mood changes [5]. Patients in advanced stages of AD suffer from severe memory loss, hallucinations, disorientation, and lack self-sufficiency, where individuals eventually die due to respiratory syndrome [6], infection or fasting [4, 7]. Primary pathological hallmarks of AD include Aβ plaques, neurofibrillary tangles (NFTs), gliosis, and neuronal loss [8–12], accompanied by cerebrovascular amyloidosis, inflammation and major synaptic changes [13–15].

## Aβ and AD pathogenesis

### Structure and function of APP

β-amyloid (Aβ) protein is the principal component of AD-associated amyloid plaques, and is produced by protease cleavage of the type I transmembrane amyloid precursor protein (APP) [16, 17]. Anywhere from 8 to 11 APP isoforms can be generated from alternative transcriptional splicing, where the 3 most common splice isoforms include the 695 amino acid form (APP695) predominantly expressed in neurons, 751 and 770 amino acid forms (APP751, APP770) expressed both in neurons and glial cells [18]. Although APP has been extensively investigated, the specific physiological function of APP remains unclear. So far, several physiological roles of APP have been proposed. The extracellular domain of APP mediates cell-to-cell adhesion to support synaptic connections. APP homodimers may function as cell-surface G-protein coupled receptors which can bind Aβ, and mediate neuronal signaling and neurotransmitter release through the activation of calcium channels [17, 19]. More specifically, APP can mediate hippocampal γ-aminobutyric acid (GABA)-ergic inhibition via direct protein-protein interactions with K^+^-Cl^−^ cotransporter 2 (KCC2), thereby stabilizing KCC2 on cell membranes. APP deficiency increases KCC2 degradation via tyrosine-phosphorylation and ubiquitination, therefore, leading to GABA reversal potential depolarization and impairment during GABA_A_ receptor-mediated inhibition [20]. Some aspects of APP function are derived from APP cleavage products such as the soluble amyloid precursor proteins (sAPP) α and β, where sAPPα function has been well characterized. sAPPα plays an important role in neuronal plasticity/survival and has been shown to be protective against Aβ-induced toxicity [21]. In addition, sAPPα can regulate neural stem cell proliferation and early developmental events in the central nervous system (CNS) [22, 23]. It has been suggested that sAPPα can inhibit excitotoxicity-induced cyclin-dependent kinase 5 (CDK5) activation and participates in various aspects of excitoprotection in response to various neuroprotective reagents [24]. Interestingly, sAPPα expression is sufficient to rescue abnormalities in APP-deficient mice [25], suggesting that sAPPα may mediate most aspects of APP function. In contrast, N-terminal fragment of APP derived from sAPPβ may be toxic, where it can bind death receptor 6 and mediate axonal pruning and neuronal cell death [26].

### APP processing

APP processing is mainly dependent on three proteolytic secretase enzymes: α-, β- and γ-secretase. Potential α-secretases include ADAM9, 10 and 17. In brain, BACE1 is the major β-secretase, while γ-secretase is comprised of at least four core components, including presenilins (PS1 and PS2), niacstrin, PEN2, and APH1 [27]. Based on its cleavage products, APP processing can be divided into non-amyloidogenic and amyloidogenic processing pathways. The non-amyloidogenic pathway involves α-secretase-mediated cleavage of full-length APP, which releases the sAPPα ectodomain outside the cell membrane, retaining an 83 amino acid-C-terminal APP fragment (α-CTF or C83) within the plasma membrane. C83 can be further cleaved by γ-secretase which then releases a small p3 fragment into the extracellular space, where the remaining APP intracellular domain is retained in the cytoplasm [27]. The amyloidogenic pathway comprises sequential proteolytic cleavage of APP by β-secretase and the γ-secretase complex. Following β-cleavage, the sAPPβ ectodomain is released, and a 99 amino acid APP carboxy-terminal fragment (β-CTF or C99) can be further cleaved by γ-secretase at various sites. APP cleavage by γ-secretase can generate amyloid peptides of varying chain lengths including Aβ37, 38, 39, 40, 42 and 43 [28, 29]. Among them, Aβ42 and Aβ40 comprise the two major Aβ species in the brain. Although soluble Aβ40 is much more abundant than soluble Aβ42, Aβ42 exhibits a higher propensity for aggregation, due to hydrophobicity within its two terminal residues. Indeed, Aβ42 is the main component of amyloid plaques and is shown to be neurotoxic [30]. Therefore, Aβ42 is thought to be a key player in initiating plaque formation and AD pathogenesis [31]. In addition, it has been shown that the levels of Aβ38, Aβ42 and the Aβ42/Aβ38 ratio in cerebral spinal fluid (CSF) can be used to distinguish AD from other dementias [32–34]. Notably, non-amyloidogenic and amyloidogenic pathways have been shown to compete, suggesting that both enhancing non-amyloidogenic pathway and reducing amyloidogenic pathway represent viable strategies to reduce Aβ generation.

In addition to the classical APP processing pathways as the above described, other types of APP cleavage may exist. A recent study shows that APP can be cleaved by a potential membrane-bound matrix-metalloproteinase η-secretase, such as MT5-MMP, which co-localizes with amyloid plaques in AD brain [35]. η-secretase-mediated APP cleavage releases a soluble APPη ectodomain and retains a membrane bound η-CTF product [36]. In addition, other soluble and lower molecular weight soluble peptides (Aη) presumably derived from BACE1 (Aη-β) or ADAM10 (Aη-α)-dependent η-CTF cleavage, or alternatively from η-secretase cleavage of sAPPα/β. Inhibition of β-secretase activity and consequent enhancement of α-secretase cleavage leads to enhanced production of a long Aη-α species, and decreased production of a shorter Aη-β species. Importantly, both BACE1 inhibitor and Aη-α can alter synaptic plasticity as evident through impaired long-term potentiation (LTP) in the hippocampus, suggesting that BACE1 inhibition may manifest adverse effects despite reductions in Aβ production [36].

Dysregulated APP processing may contribute to AD pathogenesis by elevating Aβ production, and reducing the Aβ40/42 ratio. Strongest evidence supporting a role for Aβ40/42 alterations in AD was first derived from characterization of early onset familial mutations identified in *APP* and presenilin (*PSEN1*, *PS1* and *PSEN2*, *PS2*) genes. Mutations in *PSEN1* are especially prominent in familial Alzheimer’s disease (FAD), where 221 mutations pathogenic mutations have been identified so far. Thirty-two pathogenic mutations have been described for *APP*, while 19 different pathogenic mutations for *PSEN2* have been reported [37]. Mutations in *PS1* and *PS2* primarily alter APP γ-cleavage, thereby resulting in a decreased Aβ40/42 ratio. Most FAD mutations in APP are clustered in proximity to the γ-secretase cleavage site, which may alter Aβ40/42 ratios [38]. However, the extensively characterized Swedish APP FAD mutation (APPswe, K595N/M596L) resides adjacently to the BACE1 cleavage site, thereby enhancing BACE-mediated APP cleavage [39]. Not all APP mutations are pathogenic, a rare APP protective mutation (A673T) has been identified recently, which can reduce risk of AD onset through the attenuation of Aβ production [40].

Alterations in the intracellular trafficking of APP, as well as β- and γ-secretases can also impact APP processing. β- and γ-secretases exhibit optimal APP proteolysis in acidic compartments such as late endosomes. Increased distribution of APP, β- and γ-secretases in endocytic pathways has been shown to promote Aβ generation, whereas enhanced distribution of APP and β-secretase at the cell surface can reduce Aβ production. Recent studies have identified numerous proteins that can regulate APP processing by modulating protein trafficking. For example, low-density lipoprotein receptor-related protein 1 (LRP1), an AD risk factor, is able to enhance APP endocytosis, leading to increased Aβ and sAPPβ generation [41], whereas mutation of LRP1 increases sAPPα secretion in vitro [42, 43]. Another AD risk factor sortilin-related receptor containing LDLR A repeats (SORLA) can bind and sequester APP in intracellular compartments to reduce Aβ production [44]. Members of the sorting nexin (SNX) family which are endosomal trafficking components have also been found to regulate APP processing/Aβ production by modulating the trafficking of AD-associated processing components. For instance, SNX6 can associate with BACE1 and reducing SNX6 levels results in elevated steady-state BACE1 levels as well as increased endocytic retrograde BACE1 transport, thus increasing Aβ generation [45]. SNX12 binds to BACE1, and downregulation of SNX12 increases BACE1 endocytosis and reduces steady-state levels of BACE1 at the cell surface, thereby modulating β-cleavage of APP and consequent Aβ production [46]. SNX27 regulates APP processing via two pathways: SNX27 can limit Aβ production through the interaction with PS1 which leads to destabilization of γ-secretase complex; in addition, SNX27 can enhance non-amyloidogenic APP processing through promoting the recycling of APP to the cell surface via interacting with SORLA [47, 48]. The Golgi-localized, γ-ear-containing clathrin adaptor ARF binding protein 3 (GGA3) regulates the trafficking of BACE1 to lysosomes, and modulates BACE1 levels through interactions with ubiquitin sorting machinery, where depletion and overexpression of GGA3 inversely regulates BACE1 levels [49, 50]. Markedly, changes in the expression of trafficking regulators have been observed in AD. For example, the levels of SNX12 and GGA3 are reduced in the AD brain [51]. Altogether, these studies indicate a fundamental role for APP trafficking components in Aβ generation and accumulation, and suggest that dysregulated protein trafficking may contribute to AD pathogenesis.

### Aβ aggregation and neurotoxicity

During AD pathogenesis, Aβ aggregates are assembled from Aβ monomers into a variety of unstable oligomeric species. Oligomeric Aβ (oAβ) then further aggregates to form short, flexible, irregular protofibrils, which ultimately elongate into insoluble fibrillar assemblies comprising β-strand repeats oriented perpendicularly to the fiber axis. Extracellular Aβ aggregates in their fibrillar form are resistant to hydrolytic degradation [52, 53].

The Aβ peptide is a primary component of senile plaques, and is crucial to neuronal and synaptic dysfunction during AD progression. Although Aβ monomers at physiological concentrations are generally considered to be nontoxic, multiple lines of evidence suggest that Aβ oligomers rather than Aβ fibrils are neurotoxic [54]. oAβ can induce abnormal elevations in extrasynaptic glutamate levels and subsequent extrasynaptic N-methyl-D-aspartic acid receptor (NMDAR)-mediated excitotoxicity, thereby inhibiting hippocampal LTP. This also results in postsynaptic depression and dendritic spine loss through enhancement of long-term depression (LTD)-related mechanisms. Additionally, oAβ can disrupt intracellular calcium balance, impair mitochondria dysfunction, and induce the production of reactive oxygen species (ROS). All of these events eventually lead to neuronal apoptosis and cell death [55].

### oAβ associated-receptors in neurons

Although mechanisms underlying oAβ-dependent synaptic dysfunction have not been exhaustively characterized, studies have identified several receptors which can mediate Aβ synaptotoxicity. These receptors bind Aβ with a relatively high affinity, which include the NMDAR, ephrin type-B receptor 2 (EphB2), ephrin type-A receptor 4 (EphA4), cellular prion protein (PrPc), and leukocyte immunoglobulin-like receptor B2 (Lilrb2).

#### NMDAR

NMDARs are glutamate-triggered ion-gated cationic channels which play a pivotal role in excitatory synaptic transmission, plasticity and excitotoxicity in the nervous system [56]. Seven NMDAR subunits have been characterized in total for GluN1, GluN2 and GluN3 subtypes: GluN1, GluN2A through D, and GluN3A and B. Structurally, functional NMDAR comprises two GluN1 and GluN2 or GluN3 subunits which can form a Ca^2+^-permeable ion channel [57].

Several single nucleotide polymorphisms (SNPs) in NMDAR genes have been associated with AD onset. For example, rs1806201 within exon 13 of the *GRIN2B* gene locus may play a role in modulating susceptibility to AD [58]. Additionally, frequency of the Ht2-AG haplotype in the *GluN3A* gene is higher in AD patients, indicating that GluN3A variants may confer elevated risk of AD onset [59]. Expression of NMDAR subunits has been extensively characterized in human AD brain, and in various AD models. Downregulation of the GluN1 subunit is observed in AD patient brain at different stages of neurodegenerative onset [60]. GluN1 mRNA levels were also significantly decreased in AD patients, and expression of a GluN1 isoform containing a unique N-terminus was significantly lower in AD brain compared to controls [61]. GluN2A and GluN2B expression levels (mRNA and protein) were also found to decrease in vulnerable brain regions, including the hippocampus and cerebral cortex in AD [62].

NMDARs play a critical role in regulating synaptic dysfunction in AD. oAβ may directly interact with NMDAR, as NMDAR subunits can co-immunoprecipitate with oAβ [63]. Activation of NMDARs through the accumulation of Aβ likely occurs during early stages of disease progression [64]. Similar to NMDA stimulation, Aβ evoke immediate cellular Ca^2+^ influx through the activation of GluN2B-containing NMDARs in primary neurons. oAβ has been reported to impair NMDAR-dependent synaptic LTP within hippocampal CA1 and dentate gyrus regions [65]. In addition, both synthetic oAβ and AD brain-derived Aβ can enhance NMDAR-dependent LTD [66, 67]. These alterations may be a result of Aβ-induced enhancement of NMDAR endocytosis and reductions in NMDAR expression [68]. The relevance of NMDAR in AD lends support from studies showing that partial blockade of NMDAR overstimulation with NMDAR antagonists rescues Aβ-induced LTP impairment and cognitive function in various animal models [69]. Notably, a NMDA antagonist, memantine, has been used clinically to treat AD patients. The beneficial effects of memantine may be explained partial antagonism of NMDAR activity [70].

#### EphB2

The Eph family of receptor tyrosine kinases, as well as membrane-anchored ephrin ligands, play critical roles in developing and mature nervous system [71–73]. Eph receptors and B-class ephrin ligands mediate bidirectional signaling, leading to activating signals in both ligand- and receptor-bearing cells. Eph receptors in the brain regulate maturation of dendritic spines, synaptic plasticity and neuronal-glial communication [73]. Interestingly, Eph receptors and their role in synaptic plasticity have recently been implicated in pathologies of several neurological diseases including AD [74]. Exposure to oAβ has been shown to decrease membrane EphB2 levels in hippocampal neurons [75], potentially through cross-regulatory interactions between EphB2 and NMDAR. oAβ binds to the fibronectin repeat region of EphB2, thereby triggering EphB2 endocytosis and degradation. Remarkably, overexpression of EphB2 in the dentate gyrus region in an AD mouse model reversed impairments in LTP and cognitive memory [76]. In addition, EphB2 overexpression can restore reductions in AMPAR and NMDAR levels induced by oAβ. These protective effects may be related to the PDZ-binding motif within the cytoplasmic tail of EphB2 [76, 77].

#### EphA4

EphA and EphB have opposing roles with respect to synaptic function; EphA4 is expressed on dendritic spines in pyramidal neurons, and its activation results in reduced spine length as well as spine density in acute hippocampal slices [78]. Physiological EphA4 activation at postsynaptic densities through an astrocytic ephrinA3 ligand induces retraction of dendritic spines through CDK5 and ephexin1 during synaptic pruning. To this effect, EphA4 deletion in mouse brain results in more spines, which are longer and lack organization compared to wild-type [78]. Remarkably, recent studies have established a relationship between EphA4 with AD. Increased EphA4 mRNA levels are observed in synaptosomes from AD patients [79]. Moreover, deposition of EphA4 is observed in regions surrounding senile plaques in human hippocampus [79], and greater amounts of active EphA4 are evident in AD brain [80].

oAβ can bind to EphA4 and induce its activation, and inhibition or absence of EphA4 in hippocampal neurons prevents synaptic loss [81]. The inhibitory EphA4 peptide, KYL was found to protect neurons from the synaptotoxicity with exposure to oAβ [82]. In addition, a plant alkaloid rhynchophylline was shown to block EphA4 signaling, thereby preventing LTP impairment in an AD mice model [81]. Our recent work provides a new insight into EphA4-mediated Aβ toxicity; SORLA interactions with the EphA4 receptor can consequently attenuate EphA4 activation in response to Aβ exposure. An AD-associated mutation in SORLA impairs the interaction between EphA4 and SORLA. In addition, we found that EphA4 is activated in human AD brain, and EphA4 activation correlates with decreased EphA4/SORLA interaction [80]. These findings suggest that SORLA may affect AD pathogenesis at least partially through regulating EphA4-medaited Aβ toxicity.

#### PrPC

PrPC is a highly conserved protein, which can be found in vertebrates and at all stages of development [83]. PrPC is expressed in many brain regions, including cortex and hippocampus [84], and is localized in neuronal pre- and postsynaptic compartments [85]. PrPC can mediate various functions, including neurite outgrowth, neuronal differentiation and survival [86]. A genome-wide association study (GWAS) identified PrPC as a potential high-affinity receptor for oAβ [87]. Subsequent studies showed that oAβ, especially high-molecular weight oligomers [88], preferentially bind to PrPC within an N-terminal 95-111aa segment [87]. PrPC deletion functionally restored synaptic LTP deficits induced by oAβ in different AD mouse models, such as APP/PS1 (APPswe /PSEN1△E9) [89]. Interestingly, neurons lacking PrPC are refractory to dendritic spine loss triggered by oAβ. However, oAβ interactions with PrPC have little effect on Aβ plaque deposition and glial activation [89]. Antibodies targeting various regions in PrPC abolish LTP impairment triggered by exposure to human AD brain extracts [90], and peripheral injection of these antibodies displays protective effects in AD mouse models [91].

#### LilrB2

LilrB2 is an immune inhibitory receptor which plays a vital role in suppressing the immune system and sustaining the homeostasis of the immune system [92]. Many studies have focused on the role of LilrB2 in tumors. A recent study has linked LilrB2 to AD, and suggests that human LilrB2 and its murine homolog paired immunoglobulin-like receptor B (PirB) are potential oAβ receptors [93]. Deletion of PirB rescued hippocampal LTP deficits induced by Aβ42 oligomers. In addition, PirB deficiency can rescue cognitive deficits in an AD mouse model. Mechanistically, PirB interacts with cofilin, and levels of the inactive phosphorylated form of cofilin in human AD brains appear to be reduced. Therefore, binding between oAβ and PirB would recruit cofilin-signaling modules, which leads to actin depolymerization, resulting in synaptic dysfunction and cognitive deficits [93]. Compounds inhibiting Aβ/LilrB2 interactions in vitro have been identified, and potentially bioactive Aβ/LilrB2 inhibitors such as ALI6 can inhibit Aβ-mediated neurotoxicity in primary neurons [94].

### Aβ and mitochondrial dysfunction

Multiple lines of evidence suggest that mitochondrial dysfunction is involved in AD pathogenesis [95]. Aβ accumulates in mitochondria in AD brain, which is accompanied by altered mitochondrial structure, decreased mitochondrial respiratory function and ATP production, impaired mitochondrial dynamics, and elevated mitochondria-associated oxidative stress. Aβ has been observed in mitochondria in the brain of AD patients and AD mouse models. Mitochondrial Aβ levels correlate with abnormalities in mitochondrial structure and function. For instance, decreased mitochondria associated-energy metabolism was observed in brain regions associated with amyloid plaques. Aβ also triggers abnormalities in mitochondrial dynamics; aberrant changes are also observed with mitochondrial dynamics as a result of reduced energy production. Aβ-exposure also leads to the enrichment of proteins associated with increased mitochondrial fission and decreased mitochondrial fusion [96, 97].

Evidence also suggests that oxidative insults significantly contribute to AD pathogenesis [98]. Oxidative stress manifests early in AD, which supports the notion that oxidative stress may drive Aβ-induced AD pathogenesis [99]. Mitochondria are the primary source of intracellular ROS. Aβ peptides can induce ROS production from mitochondria, leading to release of cytochrome c and apoptosis-inducing factor, thereby driving mitochondrial dysfunction, cell apoptosis and neuronal loss [97, 100]. We have recently identified a mitochondrial protein, appoptosin, as an important regulator for Aβ-induced neuronal cell death. The expression of appoptosin is upregulated in AD, where it can activate the intrinsic caspase pathway. Notably, downregulation of appoptosin can protect against Aβ-induced neurotoxicity [101]. Other mitochondrial proteins such as amyloid-binding alcohol dehydrogenase, cyclophilin D also have been shown to play a role in mitochondrial dysfunction [102–104].

## Tau and AD pathogenesis

### Tau

Human tau is encoded by the *MAPT* gene on chromosome 17 which comprises 16 exons [105]. Alternative splice variation including exons 2, 3, and 10 generates up to six differing tau isoform variants in the human brain [106, 107]. These isoforms can be distinguished from each other through compositional inclusion or exclusion of zero (0 N), one (1 N) or two (2 N) 29 amino acid inserts at the N-terminus, and three (3R) or four (4R) microtubule-binding repeats [106, 107]. In normal adult human brain, 3R and 4R tau isoforms are maintained in a 1:1 ratio. Imbalanced 3R:4R tau ratios resulting from altered *MAPT* pre-mRNA splicing have been observed in various tauopathies. For example, increased 3R:4R ratios have been observed in Pick’s disease [108], while decreased 3R:4R ratios are found in progressive supranuclear palsy (PSP) and corticobasal degeneration [109–111]. Frontotemporal dementia with parkinsonism-17 (FTDP-17) generally exhibits increased levels of the 4R tau isoform, with several exceptions [112]. Expression of tau isoforms in AD brain is complicated; although it is still under debate that whether the overall 3R:4R tau ratio is altered, several studies support a notion that 4R tau expression is increased in vulnerable brain regions and NFT bearing neurons in AD brain [111, 113–118].

As a microtubule binding component, tau promotes the polymerization and stability of microtubules [119–121]. Since tau binds to microtubules through C-terminal repeats within the microtubule-binding domain, 4R tau isoforms show a higher propensity to promote microtubule assembly compared to 3R tau isoforms [122, 123]. Tau is highly expressed in neurons in the mammalian brain, and normally localizes predominantly to axons as an important regulator of axonal transport [124–126]. However, deletion of the tau gene fails to induce problems in axonal transport, suggesting that other proteins associated with microtubule binding or regulation, such as MAP1 and MAP2 may compensate for tau [127]. Recent studies demonstrate that tau is also present in dendrites and postsynaptic compartments [128–130]. Dendritic tau may also play a role in regulating synaptic plasticity, as synaptic activity can recruit tau to the postsynaptic densities, where tau deletion in various mouse models show deficits either in LTP or LTD [129, 131, 132]. Tau distribution in neurons and its role in synaptic function is regulated by post-translational modification, including phosphorylation and proteolytic cleavage, as discussed below. Tau is also moderately expressed in oligodendrocytes and astrocytes [133–135]. In oligodendrocytes, tau plays a role in process outgrowth and myelination [133, 136–138], however, it remains unclear at this point whether tau regulates physiological functions of astrocytes. Additional physiological functions for tau include regulation of iron export and insulin signaling [139, 140].

### Post-translational modifications of tau

Various forms of post-translational modifications (PTMs) on tau include phosphorylation, acetylation, glycosylation, nitration, glycation, methylation, ubiquitination, sumoylation, truncation and prolyl-isomerization. Multiple lines of evidence indicate that PTMs regulate tau function, as well as pathogenesis of tauopathies such as AD. Alterations of tau PTMs have been observed in AD and other tauopathies. Several key tau PTMs relevant to AD pathogenesis are reviewed below.

#### Phosphorylation

Eighty-five potential phosphorylation sites (45 Ser, 35 Thr, and 5 Tyr) are present in the longest tau isoform (2N4R) in human brain [141]. Among them, more than 47 phosphorylation sites have been identified by mass spectrometry, which primarily reside in the proline-rich domain and C-terminus [141]. Hyperphosphorylated tau is enriched in paired helical filaments (PHFs) from AD patient brain or AD mouse models. Tau hyperphosphorylation may be an early event during AD pathogenesis, since increased levels of phosphorylated tau are detected in the CSF from potential AD patients at early stages of disease onset, and correlate with cognitive impairment. Therefore, phosphorylated tau species in human CSF is proposed to be a biomarker in AD diagnostics. Tau phosphorylation/hyperphosphorylation can modulate physiological and pathological tau function. Phosphorylation affects tau microtubule binding, whereas concurrent tau hyperphosphorylation at numerus sites results in tau dissociation from microtubules and enhances tau aggregation. In addition, tau phosphorylation also modulates its distribution to dendritic spines to alter synaptic function. While phosphorylation at S396 plays a key role in the induction and maintenance of hippocampal LTD [142], mutant tau hyperphosphomimetic Ser/Thr isoforms promotes tau distribution to dendritic spines and impairs synaptic function [143]. Interestingly, tau phosphorylation is not exclusively deleterious to synaptic function. Tau phosphorylation at T205 has been shown to reduce the association of tau to postsynaptic density-95 (PSD95)/NMDAR complexes and therefore to limit Aβ-induced excitotoxicity [144]. Nevertheless, hyperphosphorylation of most tau residues characterized so far is thought to be pathogenic in AD and other tauopathies.

Tau phosphorylation is regulated by multiple protein kinases and phosphatases. Tau kinases can be classified to two categories: 1) Ser/Thr kinases such as CDK5, glycogen synthase kinase 3β (GSK3β), mitogen-activated protein kinase, Ca^2+^/calmodulin-dependent protein kinase II, microtubule-affinity regulating kinase, protein kinase A (PKA), protein kinase C, Akt, TTBK1/2, CK1, DYRK1A, and 2) tyrosine kinases including Fyn, Src, Syk and c-Abl [141]. Tau is dephosphorylated by protein phosphatase 1 (PP1), PP2A, PP2B and PP5 [145–147]. Tau hyperphosphorylation may result from imbalanced activity or expression of tau kinases and protein phosphatases. In support of this, increased GSK3β expression and CDK5 activity, decreased expression of PP1 and PP2A, and decreased PP2A activity has been observed in specific brain regions in AD patients [148–152]. Since hyperphosphorylated tau species are enriched in NFTs, strategies to suppress tau phosphorylation may be a viable therapeutic strategy in AD and other related tauopathies. Unfortunately, attempts to target hyperphosphorylated tau or inhibit tau kinases have not seen success so far.

#### Lysine-based PTMs

Forty-four Lys residues may be potentially modified by acetylation, ubiquitination, sumoylation, methylation or glycation in the 2N4R tau isoform. Tau can be acetylated by the histone acetyltransferases CREB-binding protein and P300, autoacetylated through catalytic Cys291 and Cys322 residues within the microtubule binding domain, and deacetylated by SIRT1 and HDAC6 [153–155]. Acetylation of tau at Lys174, Lys274, Lys280, and Lys281 have been well characterized due to their association with AD: tau acetylation at Lys280 can only be detected in AD and 4R tauopathies such as corticobasal degeneration and PSP. Additionally, increased levels of acetylated tau at Lys174, Lys 274, and Lys281 have been observed in the brain of AD patients at varying disease stages [156–159]. Acetylation may compromise normal tau function and confer toxic properties to tau. Specifically, acetylation of tau at Lys274, Lys280 and Lys281 residues within the microtubule binding domain impairs tau binding to microtubules. Lys280 acetylation enhances fibrillization, whereas Lys274 and Lys281 acetylation promotes tau distribution to the soma and dendrites, and resulting in synaptic and cognitive dysfunction [157, 158, 160]. Tau acetylation at Lys174 reduces tau turnover and induces cognitive deficits. Acetylation may affect other PTMs in tau: since both acetylation and ubiquitination are modifications on Lys residues, acetylation may decrease proteasome-mediated tau degradation by competitively attenuating tau ubiquitination [153, 161]. The effect of tau acetylation on tau phosphorylation is dependent on acetylation at specific Lys residues. Acetylation of Lys residues within the KXGS motif reduces tau phosphorylation, acetylation of Lys274 and Lys281 does not generally affect tau phosphorylation, and acetylation of Lys280 alters certain tau phosphoresidues [155, 157, 160, 162]. Therefore, inhibition of acetylation at specific but not all Lys residues of tau maybe beneficial for AD and other tauopathies. Ubiquitination is essential to maintaining intracellular protein homeostasis, and the ubiquitin proteasome system (UPS) and lysosomal degradation pathways are both linked to tau stability and turnover. Lys48-linked polyubiquitination directs protein to UPS-mediated degradation pathways, whereas proteins conjugated with Lys63-linked polyubiquitin chains are predominantly degraded through the auto-lysosomal pathway. Both Lys48-linked and Lys63-linked polyubiquitination species have been identified in tau [163–165]. Therefore, ubiquitination plays an important role in maintaining a pool of cellular tau under physiological conditions. Accumulation of ubiquitin-conjugated tau at Lys254, 257, 290, 311, 317 and 353 has been identified in PHF from AD brain and in an AD mouse model [161, 163, 164]. Tau in PHFs is primarily monoubiquitinated, rather than polyubiquitinated; since UPS mainly mediates the degradation of polyubiquitinated protein, tau monoubiquitination may preclude tau from UPS-mediated degradation. In addition, impaired proteasomal activity induced by pathological PHF binding to proteasomes enhances the accumulation of ubiquitinated tau in AD brain [166]. Tau can be ubiquitinated by several E3 ligases such as Hsc70-interacting protein (CHIP), TNF receptor-associated factor 6 (TRAF6) and axotrophin/MARCH7 [167–169]. The relationship between CHIP and tau has been extensively studied. CHIP can interact with heat shock protein 70 (Hsp70) and induces ubiquitination of tau [170]. Induction of Hsp70 by geldanamycin promotes tau degradation both in vitro and in vivo, whereas deletion of CHIP increases the accumulation of phosphorylated tau and caspase-3 cleaved tau [167, 171]. TRAF6 induces Lys63-linked tau polyubiquitination and 26S proteasome-mediated tau degradation [168]. Axotrophin can ubiquitinate tau in vitro and impair tau microtubule-binding activity [169]. The consequence of ubiquitination on tau degradation and pathogenesis to this point, remains controversial.

Sumoylation involves the conjugation of a small ubiquitin-like modifier (SUMO) moiety on targeted lysine residues [172]. Tau can be sumoylated at Lys340 by all three major SUMO isoforms, including SUMO1, SUMO2 and SUMO3, with preferential conjugation to SUMO1 [172]. Tau sumoylation may be pathogenic, as SUMO1 immunoreactivity was found to correlate with phosphorylated tau in AD patient brain. Sumoylation enhances tau phosphorylation, but reduces tau ubiquitination and UPS-mediated degradation [173]. Factors modulating tau sumoylation and SUMO deconjugation remain unclear and require further clarification.

Tau can also be methylated on Lys and Arg residues. Methylated Lys residues mainly distribute to the projection region and tau microtubule-binding domain [174–176]. Although both mono- and dimethylation tau isoforms were initially detected in brain tissue from non-dementia human and AD patients, a recent study suggests that Lys residues are predominately monomethylated in aging or AD cohorts [174–176]. Stoichiometric Lys methylation of recombinant tau protein at high levels reduces tau aggregation [174], suggesting that upregulation of Lys methylation may be a strategy to protect against pathogenic tau aggregation. It will be of interest to elucidate how tau methylation is regulated and whether Lys methylation directly affects tau pathogenesis in vivo in future studies. Although tau Arg methylation was identified in normal and AD mouse models [161], the role of Arg methylation on tau function and pathogenesis has not been characterized.

Tau is preferentially glycated at Lys residues within the microtubule-binding domain, where advanced glycation end products are generated [177–179]. Glycated tau is only detected in PHF-tau isolated from AD brain samples, but not in soluble tau from AD or normal brain samples [177, 179]. Glycation modulates many functional properties of tau, and is associated with cellular effects, including: decreased tau microtubule binding affinity, enhanced tau aggregation, stabilization of tau aggregates, induction of oxidative stress, activation of NF-kB mediated-inflammatory pathways, and increased Aβ production [177, 179–181]. Therefore, glycation is thought to be a pathogenic form of tau PTM.

#### Glycosylation

Both N- and O-linked glycosylation have been identified in tau [182, 183]. N-glycosylation has been detected in PHF-tau isolated from AD brains, but not in healthy brain [182]. The effect of N-glycosylation on tau pathobiology is not clear. Several studies suggest that N-glycosylated tau is prone to phosphorylation, but shows reduced aggregation [184, 185].

Six O-linked Ser and Thr GlcNAcylated sites in tau have been mapped [186]. O-GlcNAcylation negatively regulates tau phosphorylation, as Ser and Thr targets for O-GlcNAcylation and phosphorylation overlap to some extent. Downregulation of O-GlcNAc transferase leads to reduced O-GlcNAcylation and increased tau phosphorylation, whereas inhibition of O-GlcNAcase reduces tau phosphorylation [187, 188]. Decreased levels of O-GlcNAcylated tau have been observed in AD brain, and inversely correlates with tau phosphorylation at multiple sites, which may contribute to abnormal glucose metabolism in AD [187]. Overall, these observations imply that decreased tau O-GlcNAcylation contributes to AD pathogenesis.

#### Truncation

Truncated tau species are derived from proteolytic processing. To date, tau proteases include caspases, calpains, asparagine endopeptidase (AEP), thrombins, cathespins, human high-temperature requirement serine protease A1, puromycin-sensitive aminopeptidase, and ADAM10 [189]. Among them, caspases, calpains and AEP have been recently become of particular interest.

Although tau can be cleaved by caspase-1, 2, 3, 6 and 7 in vitro, only caspase-2, 3 and 6 cleaved tau products have been linked to AD [190–193]. Caspase-2 cleaves tau at Asp314 and increased levels of truncated tau-314 have been described in AD brain. Tau-314 dissociates from microtubules and promotes the distribution of full-length tau and tau-314 to dendritic spines to induce synaptic and cognitive dysfunction. Indeed, downregulation of caspase-2 partially rescues memory deficits in rTg4510 tau transgenic mice [190]. Caspase-3 primarily cleaves tau at Asp421, generating a tau-421 species [191]. Elevated levels of caspase-3 and tau-421 have been observed in AD, as well as PSP [130, 191, 192]. Tau-421 colocalizes with NFTs in human AD brain, and correlates with NFT formation and cognitive impairment in aged mice [191, 192, 194]. Caspase-3 cleavage leads to the dissociation of tau from microtubules, and enhanced tau aggregation [130, 191, 195]. In addition, tau-421 can be found in PSD fractions from primary neuronal cultures [130], implicating its role in synaptic function. Indeed, memory deficits were observed in a tau-421 transgenic mouse model [196]. Recently, our group has identified appoptosin as a positive regulator for caspase-3 mediated tau cleavage. Increased levels of appoptosin associate a SNP rs1768208(C/T) associated with AD, PSP and FTD-T. Appoptosin overexpression activates caspase-3 and enhances caspase-3 dependent tau cleavage, thereby enhancing motor dysfunction in JNPL3 tau transgenic mice [130]. Since caspase-3 activation precedes NFT formation [197], and appoptosin is an upstream regulator for caspase-3, appoptosin may contribute to tau pathogenesis at early stages in AD and other tauopathies. Tau can be cleaved by caspase-6 at Asp13 (tau-13) and Asp402 (tau-402) [193, 198]. Active caspase-6 and tau-402 were observed in NFTs and neuritic plaques in AD brain [193]. In addition, tau-402 levels in CSF correlate with cognitive performance in AD patients or aged individuals [199]. These findings indicate that tau-402 may be a pathological indicator and potential biomarker for AD. However, whether tau-402 affects tau conformation and function remains elusive. Therefore, it is unknown if caspase-6 cleavage of tau plays a causative role in AD pathogenesis. Nevertheless, it is clear that caspase-mediated tau cleavage is a pathological event in AD.

Calpains are calcium-dependent cysteine proteases, which are encoded by 15 genes in the human genome [200]. Calpain-1 and 2 are abundantly expressed in the CNS [201]. Calpain-1 cleaves tau at Lys44, Arg230, Arg242, Gly323, and Gly326, while calpain-2 cleaves tau at Arg230 [202–206]. Calpain-mediated tau cleavage generates several truncated tau isoforms, including 17 kDa tau45–230, and 24 kDa tau243–441 products [204, 207]. Increased calpain activity and levels of tau45–230 has been identified in brain samples from AD and several other tauopathies, while elevated tau243–441 levels are observed in the tau transgenic Tg601 mouse model [207, 208]. Although in vitro studies show contradictory effects of tau45–230 on neuronal cell death, a recent study utilizing tau45–230 transgenic mice indicates that tau45–230 is neurotoxic, and can induce synaptic and cognitive impairment [204, 205, 209]. While tau243–441 has no apparent effects on microtubule assembly, this isoform may be pathogenic as it is able to promote tau aggregation and propagation [207]. Phosphorylation negatively regulates calpain-mediated tau cleavage: phosphorylation of tau by PKA inhibits calpain-mediated tau proteolysis, and NFTs are resistant to calpain cleavage [210, 211]. Therefore, calpain cleavage of tau may occur at early stages in AD progression.

AEP, also known as δ-secretase, is an asparagine-specific cysteine protease. Tau can be cleaved by AEP at Asn167, Asn255, and Asn368 [212, 213]. The truncated tau 1–368 (tau-368) isoform generated by AEP cleavage exhibits impaired enhancement in microtubule assembly, and shows increased propensity to form PHFs in vitro*.* AEP activity was initially reported to be increased in aged mouse and human AD brain, and tau-368 was shown to increase during aging and in AD brain [212]. However, a recent study finds no change in soluble tau-368 in AD patient brain, and only trace amounts of tau-368 were observed in insoluble tau aggregates in AD brain [212, 214]. AEP is predominately expressed in microglia [135, 213], and AEP cleaves tau without inducing tau accumulation in microglia. It is therefore likely that AEP plays a role in regulating tau degradation, rather than enhancing tau aggregation [212, 213, 215]. Exact contribution of AEP and AEP-cleaved tau to AD pathogenesis requires further investigation.

Both full-length and truncated tau isoforms can be secreted. However, only the existence of truncated tau in CSF has been confirmed [216–218], whereas the presence of full-length tau in the CSF is questionable. In CSF, full-length tau can only be detected by western blot, but not by other methods such as ELISA and IP-MS [216–219]. Using an IP-MS method, a recent study demonstrates that truncated tau isoforms exclusively exist in human CSF, whereas a small fraction of full-length tau and a large portion of truncated tau are identified in medium from iPSC-induced neurons [218]. As tau secretion is a key step to pathological tau spreading (reviewed separately below), the predominant presence of truncated tau in the extracellular space suggests that cleaved tau isoforms may contribute to the spreading of tau pathology. This possibility is likely to be of interest in future studies related to proteolytic tau processing and function. In addition, CSF and serum tau cleavage products could be potential biomarkers for AD and other tauopathies. Further investigation correlating CSF and serum truncated tau during disease onset will clarify relationships between cleaved tau isoforms and neurodegenerative progression.

### Formation and  propagation of tau  pathology

#### Tau aggregation

Tau pathology is initiated and derived from the accumulation of tau aggregates. Monomeric tau is highly soluble and is biochemically disordered, lacking a well-defined secondary structure [220]. Under certain conditions, monomeric tau can aggregate into oligomers, fibrils, filaments, and eventually NFTs. Hexapeptide VQIINK motifs in the second repeat and VQIVYK in the third repeat within the tau microtubule-binding domain are crucial for the formation of β-sheet structures and consequent tau aggregation [221, 222]. Notably, inhibitors targeting VQIINK dramatically decrease tau aggregation.

Factors that contribute to tau aggregation include abnormal PTMs on tau such as hyperphosphorylation, mutations in the *MAPT* gene, liquid-liquid phase separation (LLPS), and the presence of pathological tau seeds. The role of PTMs on tau aggregation has been described above. Both exonic and intronic mutations in the *MAPT* gene have been identified in primary tauopathies. To date, many transgenic mouse models overexpressing tau mutants have been developed, and most exhibit tau pathology and behavioral abnormalities at certain ages, supporting a pathogenic role of *MAPT* mutations. These mutations generally promote tau aggregation through altering 3R:4R tau ratios, inducing tau fragmentation, enhancing tau hyperphosphorylation or other mechanisms. Increased 4R:3R tau ratios have been found to promote tau phosphorylation and oligomerization, and to induce behavioral abnormality in a mouse model expressing human tau [223]. Both 3R and 4R tau isoforms are able to aggregate, and it is not well understood that how imbalanced 3R:4R tau ratio favors tau pathogenesis. Mutations that cause tau truncation usually also alter 3R:4R tau ratios, such as ΔK280 and ΔN296. However, it should be noted that no *MAPT* mutations have been associated with AD so far. Therefore, mechanisms underlying tau aggregation in AD may be different from those involved in tauopathies caused by the *MAPT* mutations. LLPS is a newly characterized factor that modulates tau aggregation. Tau is able to form liquid droplets, which act as sites to recruit and nucleate tubulin into microtubule bundles [224]. Peptides containing 2N4R tau microtubule binding repeats or and full-length tau undergo LLPS in solution and cells, respectively [225, 226]. LLPS may initiate tau aggregation, and this process is enhanced by tau phosphorylation, and impaired by acetylation [225–227]. Whether tau LLPS occurs in vivo, and how this process is regulated need to be clarified in future studies. Normal intracellular tau can form aggregates in the presence of tau seeds, which supports the “tau propagation” hypothesis described below.

Various cell types in the CNS may also affect pathological tau aggregation. Tau aggregates are primarily found in neurons in AD, whereas accumulation of tau can be observed in neurons, astrocytes, and oligodendrocytes in primary tauopathies [228], suggesting that cell-specific effects may be involved in tau aggregation in different tauopathies. However, comparative studies investigating tau aggregation and accumulation have yet to define these features in AD.

#### Tau propagation

NFTs first appear in layer II of the entorhinal cortex (EC) during AD onset. NFTs subsequently appear in interconnected anatomical regions within the brain, including the hippocampus and neocortex during neurodegenerative progression [229–231]. Since the spatial-temporal distribution of tau pathology correlates tightly with cognitive decay in AD patients, the severity of AD onset is classified by Braak stages which are defined by pathological NFT staining. It was previously believed that differences in vulnerability to pathogenesis in various brain regions account for the spatial-temporal characteristics of tau deposition. However, multiple lines of recent evidence indicate that prion-like tau propagation may be causal to spatial-temporal pattern of tau accumulation in AD and other tauopathies.

The “tau propagation” hypothesis lends strong support from numerous studies using mouse models. In these studies, seeded synthetic tau fibrils, or brain extracts from tau transgenic mice or human patients with tauopathy injected into the brain of tau transgenic or WT mice was found to induce pathological tau spreading at sites distal to the injection site [232–235]. In transgenic mouse models exclusively expressing human tau P301L in the EC region, pathological human tau spreads to synaptically connected regions such as dentate gyrus of the hippocampus, and induces synaptic degeneration with aging [236, 237]. In support of these results from mouse models, cellular studies demonstrate that intracellular tau aggregation can be induced by brain extracts from patients with tauopathy, tau fibrils, or even monomeric tau, and tau aggregates can be transferred between cells [234, 238–240].

Pathological tau propagation is characterized by key events in the CNS: tau seeds released from donor cells are internalized by recipient cells, which then induce aggregation and accumulation of soluble tau in the recipient cells. Alternatively, tau seeds may be transferred through cell-to-cell contact [241, 242]. Tau can be secreted under both physiological and pathological conditions, as evidenced by the presence of extracellular tau in the media of neuronal cultures, and in the interstitial fluid (ISF) of WT and tau transgenic mouse brain [238, 243–247]. Although evidence indicates that exosomes, neuronal activity, and unconventional secretory pathways are involved in tau spreading, mechanisms underlying tau release are poorly understood [248]. Exosomes are extracellular vesicles derived from endosomal compartments of cells [249]. Tau can be detected in exosomes isolated from cultures of mature neurons or microglia, and CSF and blood of AD patients [248, 250–253]. Tau-containing exosomes derived from either neurons or microglia are able to promote tau propagation [250, 251]. Tau in exosomes can be phosphorylated, truncated, or assembled into oligomers [248, 254]. Levels of exosome-associated tau are higher in CSF and blood in AD patients compared to controls [252, 253], suggesting that exosomal tau may be a biomarker for AD. Validation of these results with larger cohorts will be required. Tau is present in both pre- and postsynaptic compartments and tau seeds are propagated via neural networks [128, 130, 235–237, 255, 256]. As expected, increased neuronal activity is shown to promote both physiological and pathological release of tau in vitro, and exacerbates tau pathology in vivo [257, 258]. Tau can also be directly released from plasma membrane. This process is mediated by heparan sulfate proteoglycans (HSPGs) on the cell membrane, and is enhanced by tau phosphorylation and oligomerization [259, 260]. In summary, both normal and pathological tau seeds can be secreted. The propagation of tau pathology may be primarily influenced by tau aggregation states, although the possibility that tau seeds are released in a different manner compared to non-pathogenic forms of tau cannot be excluded.

Following its release from donor cells, tau can enter recipient cells via micropinocytosis, endocytosis, or phagocytosis [251, 261–264]. Notably, a recent study shows that monomeric tau can enter neurons through rapid endocytosis and slow endocytosis, whereas aggregated tau enters neurons primarily via endocytosis, suggesting different internalization mechanisms for different tau forms are involved [264]. Tau uptake is regulated by HSPGs in neuronal cells, and by Chemokine CX3C receptor1 (CX3CR1) in microglia [262, 263, 265]. Downregulating genes involved in HSPG synthesis, or inhibiting HSPGs greatly reduces tau uptake and propagation [262, 265].

After internalization, intracellular compartments where exogenous tau seeds interact with endogenous tau, and how tau seeds induce endogenous tau aggregation is unknown. Limited information is currently available with respect to how tau aggregation may be templated. Some studies have shown that various distinct pathological patterns of tau aggregates can be induced by distinct tau strains from tau transgenic mice or patients with different tauopathies [234, 235, 266, 267]. It will be of interest to determine whether different species of tau aggregates differentially affect brain function in further studies.

### Tau and neurotoxicity

Neurotoxic effects related to tau have been extensively studied and reviewed [268–270]. Many tau species such as tauopathy-associated tau mutants, tau with aberrant PTMs, soluble tau oligomers and tau fibrils have been shown to be neurotoxic. However, whether tau tangles are toxic still remains under debate. Tau is primarily expressed in neurons, and its subcellular distribution is primarily localized to axons where it associates with microtubules. Pathological tau has been shown to distribute to pre- and postsynaptic compartments in synaptosomal fractions from AD brain [255, 271]. Thus, pathogenic tau may impair microtubule assembly, disrupt axonal transport, impair pre- and postsynaptic functions, and induce neuronal cell death.

As described above, some FTDP-17 linked tau mutations and aberrant tau PTMs such as hyperphosphorylation and truncation can impair tau binding to tubulin and destabilize microtubules, leading to impaired cytoskeletal integrity in cultured cells. In addition, microtubule destabilization impedes axonal transport [272]. Since mitochondria can be delivered via microtubule-associated proteins mediated-axonal transport into synapses [272], tau overexpression and hyperphosphorylation can damage mitochondrial axonal transport, dynamics and function to impair neuronal viability [273]. Indeed, disrupted mitochondrial distribution has been observed in neurons containing tau aggregates in the brain of AD mice and patients [274]. How pathogenic tau species lead to aberrant mitochondrial distribution is unclear, although mechanisms related to alterations in mitochondrial fission and fusion have been implicated in this phenomenon [274, 275]. Aberrant interactions between hyperphosphorylated tau and a mitochondrial fission component, dynamin-like protein 1 (Drp1), lead to excessive fission of mitochondria in AD mice. Similar results have been observed in AD brain [276]. In addition, reductions in Drp1 can rescue mitochondrial and synaptic impairment induced by hyperphosphorylated tau in tau transgenic mice [277].

Pathological tau can cause synaptic loss and dysfunction. For instance, reduced spine density and impaired LTP is observed in tau P301L transgenic mice rTg4510 [278]. Mechanisms underlying tau synaptotoxicity are not clear, although some components have been proposed to mediate tau toxicity. For instance, Fyn kinase at post synaptic densities can modulate tau-dependent synaptic and cognitive dysfunction. Tau binds to Fyn and enhances its interactions and stabilizing effects with NMDA receptors. Deletion of tau in mice altered Fyn localization in postsynaptic compartments, and reduced NMDAR-dependent excitatory toxicity in response to Aβ [128]. Inhibition of Fyn kinase reduces tau aggregation, suggesting that tau-Fyn interactions can exacerbate tau pathology in an AD mouse model [279, 280]. Tau can also interact with the presynaptic protein synaptogyrin-3, which mediates synaptic vesicle (SV) clustering induced by pathological hyperphosphorylated tau species. SV clustering reduced synaptic vesicle mobility and release rate, impaired neurotransmission, and disrupted presynaptic function. Reducing synaptogyrin-3 levels disrupts interactions between tau and synaptic vesicles, thereby rescuing presynaptic defects induced by tau. Together, these results suggest that synaptogyrin-3 is a key modulator for tau-induced presynaptic dysfunction [281].

Unlike most cell types, neurons are non-proliferative and are quiescent upon differentiation. However, studies suggest that numerous signaling pathways triggered by neurotrophic factor deprivation, neuronal inactivity, DNA damage, oxidative stress, or excitotoxicity can elicit cell cycle reactivation, which results in increased susceptibility to cell death [282]. Some evidence suggests that tau leads to cell cycle re-entry and arrest at late onset, and supports a model where cell cycle re-entry can impact AD pathogenesis. For example, Cdc2/cyclin B1 kinase is a key regulator required to maintain neuronal quiescence. Accumulation of Cdc2/cyclin B1 in NFT-positive neurons has been observed in AD brain [283]. In addition, other cell cycle proteins are abnormally expressed in NFT-bearing neurons, including BRCA-1 and other various cyclins and cyclin dependent kinases [284].

### Links between Aβ and tau pathogenesis

Unlike mutations in *APP* and *PS1/2* that affect Aβ generation in early onset familial AD [285, 286], mutations in *MAPT* have not been associated with AD [287, 288], suggesting that tau pathogenesis may occur downstream of Aβ accumulation [289]. Indeed, Aβ can induce tau pathology in multiple APP transgenic animal models, whereas tau does not induce amyloid pathology. For instance, mouse models with high plaque loads consistently display dystrophic neurites containing hyperphosphorylated tau surrounding amyloid plaques [290, 291]. Increased levels of p-tau and conformationally altered tau were observed in transgenic rat brain overexpressing AD-associated APP/PS1 mutations in a wild-type tau background [292, 293]. Aβ may induce tau hyperphosphorylation through the activation of tau kinases such as GSK3β [294]. In addition, Aβ-induced inflammation may also contribute to tau pathology. Aβ plays a primary role in activating several innate immune pathways, causing inflammatory response and releasing inflammatory cytokines, such as interleukin-1β (IL-1β) [295, 296]. Blocking downstream IL-1 signaling pathways through exposure to an IL-1-R antibody reduced tau pathology in triple transgenic AD mouse models bearing both APP and tau transgenes [297]. Conversely, increasing IL-1β signaling pathways was shown to exacerbate tau pathology [298].

Multiple lines of evidence indicate that Aβ-induced neurotoxicity occurs in a tau-dependent manner [299]. Tau deletion can prevent neuronal cell death induced by Aβ in vitro, and re-expression of mouse or human tau in tau knockout neurons can restore Aβ-induced neurotoxicity [300]. In addition, depletion of tau can prevent Aβ-induced defects in axonal transport [127]. Animal studies also support a role for tau in mediating Aβ-induced neurotoxicity: tau deletion protects against learning and memory impairment and excitotoxicity in several APP transgenic mouse models [301–303]. Tau deletion also decreases BACE1-mediated APP cleavage and subsequent amyloid deposition [304]. Importantly, clearance of pathological tau oligomers is sufficient to alleviate cognitive impairment and reduce amyloid deposition, suggesting that oligomeric tau is a critical mediator for Aβ-induced toxicity. Aβ may trigger the transition of tau from normal to toxic states [302, 305], where toxic tau isoforms can further enhance Aβ toxicity through a potential feedback loop [299].

Additionally, tau may amplify Aβ pathogenesis through excitotoxicity and Aβ processing pathways. Tau can bind to Fyn and induce Fyn phosphorylation in AD patient brain [128]. Phosphorylated Fyn promotes interactions between NMDAR and the postsynaptic scaffolding component, PSD95, which can enhance excitatory glutamate sensitivity, thereby aggravating Aβ excitotoxicity [128]. In addition, tau can regulate Aβ through GSK3, where reducing tau levels can inhibit GSK3β activity and consequent Aβ production [306].

Further, tau can directly bind Aβ to promote Aβ aggregation. Tau binds Aβ in a stable complex, which promotes tau phosphorylation through GSK3β activation and accelerates local Aβ formation and Aβ accumulation [307, 308]. In *Drosophila melanogaster*, Aβ and tau co-expression increases tau phosphorylation and enhances neurodegenerative alterations induced by Aβ [309]. Co-localization of Aβ and phosphorylated tau are also detected in neuronal terminal synapses in AD brain [310]. Taken together, Aβ- and tau-mediated pathogenesis acts synergistically in AD onset.

## Glial contributions to AD pathogenesis

Neuroinflammation is an additional hallmark for AD, which manifests in gliosis, characterized by proliferation and activation of microglia and astrocytes, two major glial cell types in the brain. Many newly-identified AD risk genes such as triggering receptor expressed on myeloid cells-2 (*TREM2*) are exclusively expressed, or highly enriched in glial cells. Therefore, the potential involvement of glia in AD pathogenesis has recently attracted much attention. Pathogenic Aβ and tau species can induce gliosis and neuroinflammation. Reciprocally, glial cells and inflammation can regulate Aβ and tau pathogenesis. Generally, it is believed that abnormal activation of microglia and astrocytes is a deleterious event during AD onset, and inhibition of malignant glial response to pathological Aβ and tau, as well as blockade of pro-inflammatory cytokine release may impede AD pathogenesis.

### Glia and Aβ pathogenesis

Abnormal Aβ accumulation may initiate the inflammatory cascade in AD. Microglia are resident immune cells that mediate brain homeostasis by regulating immune function, phagocytosis and tissue repair function; in this context, oAβ can stimulate microglial proliferation and activation [311]. In early AD, microglial activation may be protective as activated microglia actively phagocytose and degrade oAβ. In addition, microglial activation may help neuronal repair via secreting glial-derived neurotrophic factor (GDNF) and brain-derived neurotrophic factor (BDNF). For example, administration of exogenous microglia stimulated with interferon-γ significantly enhanced BDNF and GDNF expression in ischemic hippocampus, and improved learning behavior in ischemic mice [312]. However, activated microglia can also release proinflammatory cytokines including IL-1β, IL-6, as well as tumor necrosis factor-α (TNFα) in AD, and enhance oxidative stress through induced ROS generation [313, 314]. Further, hyperactive microglia may impair synaptic function by stimulating phagocytic synaptic pruning. Therefore, chronic microglial activation during AD onset may be deleterious due to potential adverse effects associated with inflammation, neurotoxicity and degeneration. In addition, neuroinflammation can aggravate Aβ accumulation through perturbations in phagocytic Aβ uptake and clearance. It has been shown that IL-1β, lipopolysaccharide (LPS), prostaglandin E2 and tert-butyl hydroperoxide can reduce the microglial phagocytosis, thereby enhancing Aβ aggregation [315].

Notably, not all the microglia in the brain behave similarly. Recent studies identified a new microglial subtype termed “disease-associated microglia” (DAM) in animal models of AD [316]. DAM features unique transcriptional and functional characteristics [317], and are associated with altered expression of several genetic AD risk factors: apolipoprotein E (APOE), TREM2, progranulin and TYROBP (DAP12) are upregulated in DAM, whereas CD33, BIN1, PICALM and PLCG2 are downregulated [317–321]. Deletion of mouse TREM2 or expression of human TREM2 (R47H) in 5XFAD mice impaired microglia function and exacerbated AD pathology, whereas overexpression of human TREM2 has been shown to protect against Aβ pathogenesis [322–324]. An AD-associated SNP variant within the CD33 promoter region (rs3865444C) leads to overexpression of CD33 [325]. Therefore, it is possible that DAM plays a protective role during AD pathogenesis, though the exact function of DAM has not been fully determined.

Astrocytes comprise the most prominent glial cell type within the brain, and define borders separating nerve tissue from non-nerve tissue along the vascular space and meninges. Astrocyte borders and scars form functional barriers that limit the entry of inflammatory cells into the CNS parenchyma. Therefore, astrocytes have crucial roles in regulating inflammation in the CNS [326]. During AD pathogenesis, accumulation of Aβ together with proinflammatory cytokines released by activated microglia leads to astrogliosis. Activated astrocytes have bi-directional effects on AD: on one hand, they can promote degradation and clearance of Aβ mainly through the generation of APOE, a key regulator for Aβ clearance [327–329]. Conversely, activated astrocytes can aggravate inflammation by producing proinflammatory cytokines and active nitrogen and oxygen species (RNS, ROS) which interfere with synaptic germination and axonal growth [330, 331]. Additionally, Aβ can indirectly induce glutamatergic toxicity by reducing distribution of the astrocytic glutamate transporter, GLT1 (EAAT2, SLC1A2) to the cell surface [332]. Microglia may play an important role in regulating astrocytic activation; recent studies characterized a specific reactive astrocyte subtype (A1 astrocytes) induced by IL-1α, TNF, C1q and fragmented mitochondria released from activated microglia [333, 334]. The abundance of A1 astrocytes increases in neurodegenerative diseases such as AD, where A1 astrocytes have been shown to exhibit impaired phagocytic ability and reduced neuroprotective activities including their ability to support neuronal survival, outgrowth and synaptogenesis. In addition, A1 astrocytes can induce cell death in neurons and oligodendrocytes [333, 334].

Although the mechanisms underlying Aβ and glia interactions are not yet clear, growing evidence indicates that several glial receptors play critical roles in mediating Aβ-induced glial responses and functions.

### Microglial receptors

#### TREM2

TREM2 is a cell surface receptor comprising an extracellular Ig-like domain, and is abundantly expressed in microglia and macrophages [335, 336]. After ligand binding, TREM2 transmits intracellular signals through the associated transmembrane adapters DAP12 and DAP10, which recruit the protein tyrosine kinase Syk and phosphatidylinositol 3-kinase, leading to the phosphorylation of downstream players, including PI-3 K, PLC-γ and Vav2/3 [337, 338]. Genome-wide sequencing and GWAS showed that some TREM2 variants can significantly increase AD risk by 2–4 fold [296]. The most common TREM2 variant known to increase AD risk is rs75932628, which encodes an arginine-histidine mutation at amino acid 47 (R47H) [339, 340]. We and other groups have recently shown that TREM2 acts as an Aβ receptor that mediates a variety of microglial responses to oAβ, where TREM2 binds, internalizes and degrades Aβ through proteasomal pathways. Additionally, TREM2 interaction with DAP12 is enhanced by Aβ, activating downstream phosphoregulatory SYK and GSK3β pathways. TREM2 deficiency impairs microglia-mediated Aβ degradation, and reduces Aβ clearance in mouse brain with oAβ injection [341, 342]. Consistently, TREM2 deficiency in 5 × FAD mice, a genetic AD mouse model, leads to increased amyloid plaques and an increased number of dystrophic neurites [323, 343], whereas increasing TREM2 levels can reduce plaque area and cognitive impairment in AD mice [324]. In addition, AD-associated TREM2 mutations reduce TREM2/Aβ interaction [324]. Taken together, these results suggest that TREM2 plays a key role in Aβ degradation/clearance in the brain, and mutations in TREM2 may contribute to AD pathogenesis through impeding microglia-mediated Aβ degradation. Interestingly, murine and human TREM2 R47H variants may not be comparable, as murine Trem2 R47H variant results in the activation of a cryptic splice acceptor site and thereby downregulating Trem2 expression in mouse, whereas these effects are not observed in human TREM2 R47H [344, 345].

Although it has been shown that TREM2 also regulates tau pathogenesis, these results remain controversial. TREM2 deficiency leads to aggravated tau pathology, changes in microglial reactivity, and marked signaling abnormalities in mouse models expressing all six isoforms of human tau at 6 months [346]. However, TREM2 deletion does not affect tau phosphorylation and aggregation in tau P301S transgenic PS19 mice, but alleviates gliosis and brain atrophy at 9 months of age [347]. In addition, TREM2 deficiency or a TREM2 R47H mutant can reduce microgliosis around Aβ plaques and promote seeding and transmission of tau aggregates in neuritic plaques [348]. Therefore, TREM2 may play different roles during different stages of AD progression.

#### LRP1

LRP1 is a type I transmembrane glycoprotein which mediates trafficking and degradation of a variety of ligands, including APOE and Aβ [349–352]. In the CNS, LRP1 is highly expressed in various cell types such as neurons [353, 354], astrocytes [355, 356] and microglia [357, 358]. In neurons, LRP1 can regulate APP trafficking and Aβ generation, though contrasting results have been obtained in different experimental models [359]. In addition, neuronal and astrocytic LRP1 regulates Aβ clearance via mediating Aβ uptake and degradation [355, 360]. However, LRP1-mediated internalization may not be responsible for soluble Aβ uptake in microglia, as blockade of LRP1 by an antagonist failed to impair microglial uptake of aggregated Aβ [355]. Expression of LRP1 in microglia is likely protective; LRP1 deletion or downregulation in microglia increased LPS-induced inflammatory response, including induction of ameboid morphology and release of pro-inflammatory cytokines [357, 361]. Mechanistically, LRP1 can suppress microglial activation by modulating c-jun N-terminal kinase, as well as NF-κB signaling pathways [357]. It remains unclear whether LRP1 affects microglial response to Aβ.

#### Other microglial Aβ related receptors

Microglia may express other putative Aβ receptors, including Toll-like receptor 2/4 (TLR2/4) [362], complement receptor 3 (CR3) [363], Fc γ receptors IIb (FcγRIIb) [364], CD36 [365, 366], advanced glycation end product receptor (RAGE) [367]. These receptors cooperatively bind, internalize and clear Aβ, in addition to modulating microglial activation.

TLR2 can interact with aggregated Aβ and reduce microglial neuroinflammatory response triggered by aggregated Aβ [368]. In addition, TLR2 deficiency can enhance microglia-dependent Aβ phagocytic uptake [368]. TLR4 can participate in AD pathogenesis and induce microglial inflammation phagocytosis through interactions with Aβ [369]. TLR4 activation induced NF-κB nuclear translocation, leading to the production of proinflammatory mediators [370]. Additionally, TLR4 may regulate Aβ accumulation, as AD mice carrying loss-of-function TLR4 mutants display more Aβ deposits compared with control AD mice at 9 months [371]. Dysregulation of the complement system may also contribute to AD pathogenesis: C1q can enhance proinflammatory cytokines production induced by Aβ42 [372]. In addition, C1q and a complement receptor CR3 has been shown to mediate early synaptic loss in an AD mouse model [373]. oAβ injection in WT mice increased synaptic loss and microglial phagocytic activity, while inhibition of CR3 activity could ameliorate synaptic loss and dysfunction caused by oAβ [373]. CD36 can bind to oAβ and contributes to AD pathogenesis by regulating cerebral inflammation in microglia [341]. Fibrillary Aβ (fAβ)-induced secretion of inflammatory factors and the recruitment of microglia /macrophages were significantly reduced in CD36 KO mice [374]. Furthermore, CD36 mediates fAβ-induced signal cascade which leads to the production of ROS and chemokines [366]. The RAGE receptor binds to multiple ligands and is a member within the immunoglobulin receptor superfamily. In addition to advanced glycation end products, RAGE can bind to a variety of ligands, such as Aβ, nerve axon growth factor, S100 protein, starch peptide and thyroxine transferase. In AD patient brain, RAGE binding to Aβ can promote microglia migration to amyloid plaques and NF-κB activation, consequently leading to neuroinflammatory activation [367]. In APP transgenic mice, overexpression of RAGE in microglia increases glial infiltration and Aβ accumulation, and exacerbates cognitive function [375]. Neuronal FcγRIIb can bind to Aβ42 with a high affinity, and mediate neurotoxicity and memory impairment triggered by Aβ [240, 376–380]. Since FcγRIIb is predominantly expressed in microglia, it is likely that FcγRIIb also plays a role in mediating Aβ-induced microglial response.

Although many receptors have been shown to mediate microglial response to Aβ, several critical questions remain open: (1) Which receptor plays a key role in Aβ-induced microglial activation? (2) What is the relationship between these receptors in AD context? (3) Is activation or inhibition of microglia beneficial to AD?

### Astrocytic receptors

#### α 7 subtype of nAChR (α7nAChRs)

Nicotinic acetylcholine receptor (nAChRs) is a classical neurotransmitter receptor which is widely distributed in the CNS, and participates in a variety of important physiological functions such as cognition [381]. In the CNS, nAChRs are expressed in neurons and glial cells, including microglia, oligodendrocytes and astrocytes, with highest expression in astrocytes among the glial cells [382, 383]. Previous studies have shown that cognitive deficits associated with AD may be partly caused by dysfunction of α 7 subtype of nAChR (α7nAChRs) in hippocampal neurons [384]. α7nAChRs activation results in Ca^2+^ influx and participate in the release of neurotransmitters; α7nAChRs also regulate neuronal excitability and LTP response, implicating a role for these receptors in neuronal function [385–387]. In addition, Aβ42 oligomers released from neurons can bind directly to α7nAChRs in adjacent astrocytes, thereby inducing astrocytic glutamate release [380]. Excreted glutamate can activate extrasynaptic NMDAR in neurons residing within neuron/astrocyte conjugates, resulting in Ca^2+^ efflux. This triggers multiple events, including mitochondrial dysfunction, caspase 3 activation, tau hyperphosphorylation, and excessive production of NO, ROS and VEG-F. These events result in damage to dendritic spines and neuronal synapses, disrupting neuronal/astrocytic communication [380, 388].

#### Calcium-sensing receptor (CaSR)

CaSR is a member of family C of G protein coupled receptors (GPCRs) [389, 390]. CaSR proteins predominantly form homodimers (CaSR/CaSR) or heterodimers (CaSR/mGluR), although CaSR also functions as monomers [391]. CaSR primarily mediates homeostasis of free calcium [392], and regulates intracellular signals resulting from Ca^2+^ influx. CaSR is expressed in all cell types within the CNS including astrocytes, and almost all brain regions with enriched expression in the hippocampus [393–395]. In the brain, CaSR plays an important role in axonal and dendritic development, cell proliferation and differentiation, the migration of neuronal and glial cells, and synaptic plasticity [396–398].

Growing evidence indicates that CaSR in astrocytes plays an important role in inflammation and degenerative brain diseases such as AD [399, 400]. Exogenous Aβ42 oligomers bind to CaSR in neurons and astrocytes, thereby activating intracellular signaling pathways that block proteolytic degradation of Aβ42 oligomers, leading to intracellular accumulation of Aβ [401]. Moreover, interactions between Aβ42 oligomers and CaSR can also induce NO production/secretion, and expression of nitric oxide synthase-2 as well as vascular endothelial growth factor-A through activation of MEK/ERK-dependent pathways, thereby aggravating neuroinflammation [394, 402]. The CaSR inhibitor NPS2143 can inhibit fibrillary Aβ25–35-induced Aβ42 production and inflammation/neurotoxicity [402]. In conclusion, the role of CaSR in Aβ production and tau phosphorylation may implicate its modulation as a promising target in AD therapeutics [403].

#### Other Aβ related receptors in astrocytes

In contrast to microglia, less studies describe a phagocytic role for astrocytes in AD [404–407]. Blocking receptors including CD36, CD47, and RAGE with neutralizing antibodies can attenuate astrocytic phagocytosis of Aβ, implicating they are putative Aβ receptors in astrocytes [407]. In addition, activation of RAGE may lead to pro-inflammatory changes with Aβ exposure in astrocytes [408]. RAGE co-localizes with intracellular APP/Aβ in neurons, and human tau in astrocytes in the CA1 region, and its expression increases in the 3xTg-AD mouse model, suggesting that RAGE may be involved in AD pathogenesis [409].

### Glia and glymphatic pathway

The glial-lymphoid pathway, or glymphoid pathway, is required for fluid homeostasis within the CNS [410]. This pathway comprises a periarterial CSF inflow channel, and a perivenous ISF outflow channel. These two channels are connected by Aquaporin-4 (AQP-4) on astrocytes [410], whereby CSF flows into the cerebral stroma from the periarterial space and mediates fluid exchange with ISF. Metabolites and tissue fluid enter the perivenous space during exchange, ultimately feeding into cerebrospinal fluid circulation, cervical lymphatic vessels, or meningeal lymphatics [410–412]. Exchange between CSF and ISF in the glymphatic system removes metabolic waste and maintains the normal physiological function in neurons and synapses [413]. Studies have demonstrated a close relationship between the glymphatic system and AD. AD patients show altered CSF dynamics, thereby inducing impairments in CSF-dependent Aβ clearance and consequent pathological Aβ accumulation [414]. Moreover, inhibition of glymphatic transport leads to a significant accumulation of Aβ in APP/PS1 mouse brain [415]. On the other hand, Aβ accumulation hinders glymphatic circulation to aggravate parenchymal Aβ deposition and neuronal death. Although mechanisms have yet to be fully defined, Aβ deposition may impair low-resistance fluidity in the perivascular space within the glymphatic circulation system [416].

In addition, perivascular AQP4 dysfunction is a potentially important factor in accelerating AD pathogenesis [417]. AQP4 deletion was found to increase Aβ accumulation and astrocytic atrophy in APP/PS1 mouse brain, with consequent effects on cognitive impairment [418]. Loss of polarized basal AQP4 distribution to endfeet in post-mortem AD patients was significantly lower than age-matched controls [417]. The glymphoid pathway is also affected by other factors such as sleep. Sleep can increase CSF circulation and accelerate transport and clearance of Aβ [419]. Chronic sleep deprivation was shown to enhance Aβ plaque deposition and pathological tau spreading in mice [420]. Together, these results suggest that defects in glymphoid function can promote pathogenesis of AD. Thus, restoring and enhancing glymphatic circulation may be potentially effective in AD prevention and treatment.

### Glia and tau pathogenesis

Given that gliosis is observed in many tau transgenic mouse models and tauopathy patients in the absence of Aβ pathology, pathogenic tau species can activate microglia and astrocytes independently of Aβ. Tau-dependent microglial activation can enhance secretion of pro-inflammatory cytokines such as IL-1β, IL-6, and TNF-α [421–423]. Although how tau activates microglia to induce inflammation is poorly understood, recent transcriptomic studies demonstrate a role for NF-κB activation and NLRP3-ASC in this process [424, 425]. Notably, Aβ also can activate NF-κB signaling and the NLRP3-ASC inflammasome [426, 427], suggesting that Aβ and tau share common mechanisms in microglia activation.

Microglia modulate tau pathogenesis through direct and indirect mechanisms. For example, microglia can directly promote tau clearance through internalizing and degrading pathological tau from AD brain [428], where mechanisms underlying this phenomenon remain elusive. Several studies demonstrate that CX3CR1 plays an important role in mediating microglial phagocytosis and tau degradation. CX3CR1 binds to tau, and to a lesser extent to hyperphosphorylated tau [263]. CX3CR1 deficiency impairs microglia-mediated tau internalization in vitro, and promotes the accumulation of hyperphosphorylated tau in vivo [263, 429]. In addition to effects on modulating tau clearance, microglia may also influence tau propagation through the formation of exosomes, as microglia depletion or inhibition of exosome synthesis can block the propagation of hyperphosphorylated tau [251]. Microglia can also indirectly modulate tau pathogenesis through inflammatory pathways; pro-inflammatory cytokines released by activated microglia can enhance tau pathology through activating tau kinases, such as p38 and CDK5 [430, 431]. Therefore, pathogenic tau and microglia activation may form cyclical pathogenic events during AD development.

Astrocytes are directly involved in tau pathogenesis. Although tau primarily accumulates in neurons, tau deposition can also be observed in the astrocyte nucleus in AD brain [432, 433]. Phosphorylation, fibrosis and asymmetric accumulation of tau in astrocytes increased with age in mice expressing P301L tau [434]. Similar to microglia, astrocytes can also phagocytose extracellular tau and contribute to tau spreading through transcription factor EB [435]. Glial tau has been shown to mediate toxicity through non-cell autonomous changes in neurons, and autonomous effects in glial cells. Accumulation of tau in astrocytes alters astrocytic function, induces neuronal degeneration and promotes cell death through a serial degenerative events, such as boosting blood-brain barrier (BBB) collapse, and inducing expression of heat shock proteins with low molecular weight [228, 434, 436, 437]. In addition, pathological tau may impair astrocyte-mediated glutamate transport, resulting in pathological glutamate accumulation in the brain and the consequent excitotoxicity [438, 439].

In conclusion, it is likely that tau pathogenesis is triggered by Aβ in AD, where pathogenic tau and Aβ synergistically contribute to gliosis and neuroinflammation. Reactive glial cells together with inflammatory components further promote Aβ and tau pathogenesis to aggravate the neurodegeneration.

## Factors that contribute to AD pathogenesis

### Genetic risk factors

Genetic susceptibility is a prevalent factor in determining AD onset and pathogenesis, where heritability of various genetic factors are estimated to contribute to ~ 60–80% of all AD cases [440]. Although APOE (APOE ε4 in particular) was previously implicated as the sole genetic risk factor for sporadic AD, recent whole-genome sequencing studies and GWAS analysis identified additional genetic factors associated with AD risk. These risk genes include *TREM2, CD33, CR1, ABCA7, SHIP1, BIN1*, *CD2AP*, *CLU*, *EPHA1*, *PICALM* and *MS4A* [441–445]. Meta-analysis of late onset AD (LOAD) datasets *identified other risk genes: CASS4*, *CELF1*, *DSG2*, *HLA, DRB5, DBR1*, *FERMT2, NPP5D*, *MEF2C*, *NME8*, *SLC24H4 RIN3*, *SORL1*, *ZCWPW1* [378]. Understanding the relationship between these AD risk genes and their role in modulating cellular and neuropathological features in AD will undoubtedly provide insight into mechanisms underlying AD onset. Here, we summarize the impact and current knowledge with respect to important genetic risk factors in AD pathogenesis, with emphasis on APOE, CD33, BIN1, SORLA and PU.1.

### APOE

APOE is a 299 amino acid glycoprotein mainly produced by liver, where liver-derived APOE accounts for more than 75% of the total APOE in the body. The brain is the second-highest source for APOE. In brain, APOE is highly expressed in astrocytes and microglia, and in neurons under stress [446–448]. The *APOE* gene locus on chromosome 19 comprises three allelic variants: APOE2 (ε2), APOE3 (ε3) and APOE4 (ε4) [449, 450]. ε2 encodes Cys residues at the position 112 and 158, ε3 encodes a Cys residue at position 112 and a Arg residue at position 158, and ε4 comprises Arg residues at both positions [447]. Structural and functional differences in APOE isoforms may be due to differential charge properties of the variant amino acid residues in APOE alleles. Compelling evidence demonstrates that the ε4 allele is potently associated with late AD onset [318, 443, 451]. The global frequency of the human ε4 allele is 13.7%, and the frequency of ε4 carriers is increased to 40% in AD patients [452, 453]. APOE ε4 affects AD risk and age of onset dose dependently [452, 454]. Clinical incidence and average age of AD onset was found to be 91% at 68 years of age in ε4 homozygous carriers, 47% at 76 years of age in ε4 heterozygous carriers, and 20% at 84 years of age in non-ε4 individuals [453].

In APP transgenic mouse models, genetic expression of human APOE4 was found to promote Aβ seeding, accelerate Aβ oligomerization and deposition in the brain [455, 456]. Lentiviral expression of human APOE4 in AD mouse brain also increased ISF -oAβ levels and aggravated plaque deposition, while APOE2 was observed to reduce Aβ accumulation [457, 458]. In AD patients, APOE4 enhances Aβ deposition, where 40.7% of middle-aged APOE ε4 carriers featured senile plaques, compared to 8.2% non-carriers identified with senile plaques [459]. In addition, APOE4 carriers display decreased Aβ42 levels in CSF compared to non-carriers [460, 461]. Furthermore, APOE4 is associated with enhanced memory impairment and memory loss. Studies showed that APOE4 carriers featured decreased cortical thickness and smaller hippocampal volume, and with an advanced mild cognitive impairment (MCI) age compared to non-carriers [462–464]. Additionally, recent studies indicate that APOE4 can impact neuronal synaptic activity and function, astrocyte-associated lipid metabolism, and immune reactivity of induced pluripotent stem cell-derived microglia models. Compared to isogenic APOE3, APOE4 variants featured an increased number of synapses and elevated Aβ42 secretion in neurons, while APOE4 astrocytes showed impaired Aβ uptake and cholesterol accumulation [465]. Of note, APOE4 also triggered inflammatory cascades, leading to neurovascular dysfunction, degeneration of the BBB, consequent penetration of toxic proteins from blood into the brain and reduced length of small blood vessels [466]. Thus, APOE4-related cerebrovascular injury may play a key role in AD pathogenesis. Interestingly, a potentially protective mutation in APOE3 (Christchurch, R136S) has been recently identified. One particular case was reported where a woman carrying a fully-penetrant familial early-onset PS1 E280A mutation featured normal cognitive function until seventies despite an abnormally high Aβ load, and showed limited tau pathology which correlated with two copies of the APOE3 R136S allele [467].

APOE also affects tau pathogenesis and tau-mediated neurodegeneration [468]. APOE4 significantly aggravated tau-mediated neurodegeneration in a tauopathy mouse model and induced tau aggregates in brain, while genetic ablation of APOE attenuated tau-induced neurodegeneration [469, 470]. In addition, APOE ε2 is also associated with increased pathological tau levels in the presence of amyloid [471, 472]. Studies have shown that hyperphosphorylated tau species, tau aggregates and behavioral abnormalities were observed in APOE ε2/ε2 mice [471]. However, the association between these findings and AD progression is unclear. Thus, further studies characterizing the pathobiology of APOE in the context of AD are required to identify the association of this risk factor and AD onset.

### CD33

CD33 is a type of sialic acid-binding immunoglobulin-type lectins which is mainly expressed in microglia in the brain [473]. In 2011, GWAS analysis linked the rs3865444 CD33 SNP to decreased AD risk [445, 474]. Generally, CD33 expression levels are elevated in AD, where deficiencies in CD33 promotes protective effects including enhancing microglial uptake of Aβ42, and reduces Aβ pathology in an AD mouse model. The rs3865444 variant was shown to reduce the expression of CD33, thereby promoting protecting effects potentially through CD33 downregulation [325]. It will be of interest in future studies to determine whether CD33 can directly bind Aβ and act as a bona-fide Aβ receptor.

### *BIN1*

Bridging integrator 1 (BIN1) has been identified as the most important genetic risk factor in LOAD after APOE [475]. BIN1 is expressed in all neural cell types, and is highly enriched in oligodendrocytes and microglia [476]. Some studies have shown that BIN1 expression is elevated in AD patients [376, 477]. Although results with respect to whether BIN1 can affect AD pathogenesis remain controversial, it seems that BIN1 may affect AD risk by regulating tau pathology. BIN1 overexpression has been shown to reverse memory deficits in tau transgenic mice, and neuronal BIN1 expression is inversely correlated with pathological tau propagation [478, 479]. However, deletion of BIN1 in microglia reduces tau secretion and spreading in PS19 tau transgenic mice, suggesting BIN1 may act differentially in neurons and microglia. In addition, the SNPs of BIN1, such as rs744373 and rs7561528, may contribute to AD susceptibility by impacting brain structure and function [480, 481].

### *SORLA*

SORLA is encoded by the *SORL1* gene.  SNPs in SORLA can either increase or reduce AD risk. For instance, rs668387, rs2070045, rs11218343 and rs3781834 appear to be protective [474, 482], whereas other variants of *SORL1*, such as rs143571823, aggravate AD pathogenesis [483]. SORLA is involved in APP processing, Aβ secretion and Aβ turnover [484]. Overexpression of SORLA in neuronal cells can block amyloidogenic processing and reduce Aβ production [485], whereas loss of SORLA increased extracellular Aβ levels and plaque deposition in several AD mouse models [486, 487]. In addition, we recently reported that SORLA can interact EphA4 and inhibit Aβ-induced EphA4 activation, thereby reducing oAβ-induced synaptotoxicity [488]. Thus, SORLA may protect against AD pathogenesis via multiple mechanisms. As various AD-associated coding mutations in addition to G511R and Y1816C have been identified for SORLA, it will be of interest to determine how mutations in SORLA affect AD pathology and brain function.

### *PU.1*

PU.1, encoded by *SPI1*, is an important myeloid transcription factor [489]. PU.1 is specifically expressed in microglia in the CNS, and is fundamental to microglial development [490]. Studies have shown that PU.1 can regulate microglia and macrophage function such as phagocytosis and inflammatory response. SPI1 depletion down-regulates expression of phagocytosis related genes, thereby impairs microglia-mediated phagocytosis. Reducing PU.1 levels through miR124 overexpression can decrease expression of TNF-α, iNOS and MHC-II, thereby suppressing neuroinflammatory response in macrophages [491]. In addition, PU.1 can regulate expression of genes associated with AD risk or onset, including *ABCA7*, *CD33*, *TREM2*, *MS4A4A*, *MS4A6A*, *TYROBP*, *Aif1*, and *MYBPC3* [492, 493]. GWAS studies suggest that reduced PU.1 expression associates with delayed AD onset [492]. Although these findings suggest that PU.1 can modulate AD pathogenesis, mechanisms underlying PU.1-dependent pathogenic events require further study.

### Aging

Aging is the greatest risk factor for sporadic AD. In the USA, the prevalence of AD in individuals over 65 years of age is ~ 10%, over 85 years is ~ 32%, and over 95 years is ~ 50% [494]. Neurons in AD brain feature aging hallmarks including genomic destabilization, decreased telomere length, alteration in epigenetic signatures, and mitochondrial dysfunction [495]. Increased DNA oxidation was observed in post mortem AD brain [496], MCI [497], and preclinical AD [498], and may exacerbate AD progression [499, 500]. Increased DNA damage in AD patients may result from deficiencies in base excision repair [501]. AD mouse models with Polβ heterozygosity feature defective DNA repair and showed increased synaptic and cognitive deficits, neuronal dysfunction and cell death [502].

Telomeres comprise DNA sequence repeats at chromosome ends. Characteristic telomere shortening is observed during cellular aging, and has been linked to increased risk of dementia in AD [503]. For example, an allele on chromosome 10p12–14 (40 centimorgan from the telomere) has been associated with increased AD risk [504]. Telomere shortening may be accelerated by oxidative insults, inflammatory elements, excessive stress, and many other risk factors related to the AD onset [505].

Epigenetic mechanisms contribute various aspects of age-related events in neurodegenerative disorders such as AD. Diverse neurological phenotypes and biological processes are regulated by epigenetic mechanisms, including learning, memory and behavior [506, 507]. For example, H4K12 histone acetylation is decreased in aged mice, leading to deficits in the expression of genes associated with learning and memory [170]. Histone acetylation is regulated by histone acetyl transferases and histone deacetylases (HDACs). Expression of HDAC2 increases with aging in AD mouse models and patients [508]. HDAC2 overexpression reduces dendritic spine density, and impairs synaptic plasticity and memory by blocking expression of genes related to neuroplasticity, whereas downregulation or inhibition of HDACs has been proven successful in restoring synaptic and cognitive function in AD animal models [508, 509]. DNA methylation is associated with development and aging, and can be used as an epigenetic clock to predict the age of various cell and tissue types [510]. During aging, global DNA hypomethylation is observed in many species including rat, mouse, and human [511]. Hypomethylated enhancer regions have been recently identified in AD neurons, where they may affect the expression of AD-relevant genes including tau kinases and BACE1 [512]. However, increased levels of DNA methylation in AD brain has been reported in another study, where changes in methylation of AD risk genes such as *SORL1*, *ABCA7* and *BIN1* are associated with AD pathology [513, 514]. Contrasting results from these studies may be due to different experimental contexts (neurons vs. brains) and differing experimental methods used in the studies. Nevertheless, changes in DNA methylation and its functional consequence in AD warrant further investigation.

Reductions in metabolic pathways related to glucose consumption have been well-characterized in AD, and is likely caused by mitochondrial dysfunction [515]. Aging typically leads to decreased ROS clearance and elevated ROS activity. Excessive ROS accumulation can further aggravate oxidative stress and mitochondrial DNA damage and dysfunction [516]. The mitochondrial cascade hypothesis proposes that mitochondrial dysfunction is the primary trigger for events leading to sporadic late AD onset [517]. In addition, defects in autophagy/lysosome pathways that remove damaged mitochondria are also impaired in AD, thereby enhancing the accumulation of dysfunctional mitochondria [518]. A recent study reports that impairment of mitophagic activity can induce synaptic dysfunction and trigger cognitive deficits by enhancing Aβ and tau accumulation, and stimulation of mitophagy reverses memory loss in nematode and mouse models of AD [519].

Blood and blood-derived factors may be involved in aging-induced cognitive impairment. A study using a parabiosis mouse model comprising young and old mice indicates that blood from young mice may rejuvenate organs, where old mice showed improvements in synaptic plasticity and cognitive behavior [520]. Interestingly, parabiotic conjugation of AD mice with young WT mice was found to restore synaptic and neuronal protein levels in AD mouse brain, reversed aberrant ERK signaling, and improved spatial and associative memory in AD mice [521]. This has given way for clinical trials (Clinical Trials.gov identifier: NCT02256306). Although how young blood reversed aging-induced cognitive defects is not clear, a recent study suggests that tissue inhibitor of metalloproteinases 2 (TIMP2) plays a critical role in mediating human cord plasma-induced beneficial effects on synaptic and cognitive function in aged mice [522].

Aged glial cells may also contribute to AD pathogenesis. Recent transcriptomic analysis of human cortical microglia implicates that genes involved in actin assembly normally required to mediate morphogenic changes in microglia are downregulated during aging. As dynamic filamentous actin (F-actin) assembly is essential to microglia morphogenesis, migration, and Aβ uptake/clearance, these results suggest that fundamental microglial functions are impaired with age [523]. Other studies have also shown that young microglia can restore defects in Aβ clearance in aged microglia, where aged microglia exposed to conditioned media from young microglia, or granulocyte-macrophage colony-stimulating factor treatment could reduce amyloid plaque size in a mouse model ex vivo [524]. In addition, aged microglia may promote the conversion of astrocytes to a neuroinflammatory A1-state through microglia-derived cytokines. Indeed, A1-astrocytes are abundantly prominent in aged brain under both normal and LPS stimulated conditions [525]. It will be of great interest to explore the relationship between aged microglia, astrocytes and neurons in future studies.

### Environmental factors

#### Viral and bacterial infection

A potential role for microbes and antimicrobial defense in AD pathogenesis was initially hypothesized in 1952 [526]. Since the 1980’s, several groups proposed that AD onset bears similarity to subacute sclerosing panencephalitis, caused by the lentiviral form of herpes simplex virus (HSV) [527, 528]. Many studies have linked AD to a diverse variety of bacterial and viral pathogens [529–531]. Related pathogens include Helicobacter pylori, various bacteria of the liver, gut, lungs (pneumonia) and mouth, as well as viruses to include Epstein Barr virus, CMV, HIV, oral herpes HSV-1, genital herpes HSV-2, human herpesvirus (HHV)-6A/HHV-7 (recently reviewed in [530]). These pathogens can infiltrate the CNS and dysregulate AD-associated neurological function.

The role of HSV-1 has attracted much recent attention in AD pathogenesis. HSV-1 is a neurotropic DNA virus, and normally manifests latent infections in the trigeminal ganglion with periodic reactivation. Meta-analysis from various literature databases indicate that AD risk increased 1.3 times with HSV in the brain, and risk increased 2.7 times in concurrent HSV-1/APOE4 carriers compared to controls [532]. In APOE4 carriers, latent HSV-1 is intermittently reactivated by immunosuppression, peripheral infection and inflammation, followed by neurological damage and AD onset [533]. Epidemiological studies indicate that HSV-positive individuals feature markedly higher risk in developing AD compared to seronegative subjects, and antiviral therapy reduced AD onset [533]. In mice with recurrent HSV-1 infection, HSV-1 was shown to spread and proliferate in different brain regions following reactivation by thermal stress. This was accompanied by the occurrence of pathological events associated with AD including Aβ deposition, tau hyperphosphorylation and neuroinflammation [534]. Additionally, chronic HSV-1 infection induced persistent microglial activation, consequently inducing antiviral IFN-β expression, while also generating neurotoxic factors, such as ROS, TNF and NO. ATP and MMP3 released from damaged neurons then acts to further activate microglia. Together, chronic activation of microglia mediated by HSV-1 infection triggers a vicious cycle of CNS inflammation [535]. Of note, recent studies showed that Aβ is an antimicrobial peptide that protects the body from fungal and bacterial infections. oAβ binds to herpesvirus surface glycoprotein and protects 5 × FAD mice from HSV-1 by accelerating Aβ deposition [536]. A recent multi-omic study identified an enrichment of HHV-6A and HHV-7 in AD patients compared to controls [529–531]. Significant overlap exists between the expression of AD-associated genes and genomic viral load in the CNS; for example, viral abundance may determine AD progression through the regulation of genes associated with APP processing [529–531].

Periodontal bacterial infection by pathogens such as *Porphyromonas gingivalis* may also play a role in AD. Specific proteins and DNAs from *P. gingivalis* have been identified in AD brain. Oral *P. gingivalis* infection increases Aβ42 generation, where Aβ42 can also be toxic to *P. gingivalis* [537]. In addition, inhibition of Gingipain, a virulence factor produced by *P. gingivalis* can effectively reduce *P. gingivalis* brain infection and the consequent toxic effects in the hippocampus [537]. Although this study implies that *P. gingivalis* may contribute to AD pathogenesis, further evidence may be required to confirm the association of *P. gingivalis* and AD.

#### Metal ions

Post-mortem analysis in AD patients reveals the accumulation of metal ions such as copper, iron and zinc (5.7, 2.8 and 3.1 times, respectively) over levels observed in normal brain, demonstrating a close correlation between AD and redox metal dysregulation [538]. The distribution of these metals is closely related to Aβ and tau metabolism. Copper, iron and zinc deposits are observed within the core and periphery of senile plaques, and co-localize with Aβ [539]. Copper overload increases APP expression and Aβ generation, while overexpression of CUTA  (the mammalian CutA divalent cation tolerance homolog of* Escherichia coli*), a BACE1 trafficking regulator, attenuates Aβ production without affecting APP expression [540]. Use of Cu^2+^ chelators can inhibit ROS production triggered by Cu-Aβ, and reversed episodic memory impairment in non-transgenic AD mice [538, 541]. Accumulation of copper is also observed in the NFTs [506]. Copper can bind to tau in vitro [542, 543], and enhance tau phosphorylation by activating CDK5/P25 in AD transgenic mice (APPswe, PS1, P301Ltau) [544, 545]. Iron can affect lipid peroxidation through interactions with iron-dependent oxidases such as lipoxygenase, subsequently activating ferroptosis to accelerate AD progression [541, 546, 547]. Zinc may aggravate AD pathogenesis, as zinc can bind Aβ and promote Aβ accumulation. In addition, the accumulation of zinc can cause synaptic and memory defects [548]. Mechanistically, high concentrations of Zinc released into the synaptic cleft can induce neurotoxicity through NMDAR and AMPAR inhibition [548]. Of note, the presence of magnesium ions in vivo may have protective effects in AD; studies have shown that AD is associated with deficiencies in magnesium (Mg^2+^) in serum or brain [549, 550]. Reduced Mg^2+^ levels can decrease Ca^2+^ influx mediated by NMDAR and damage-associated learning and long-term memory deficits in *Drosophila* [482]. In addition, Mg^2+^ treatment can reduce soluble Aβ by stabilizing BACE-1 expression, thus reversing cognitive impairment and synaptic loss in AD mice [551]. Together, restoration of metal ion balance in the brain may be beneficial to AD.

#### Stress

Growing evidence suggests that long-term exposure to stress is a risk factor for AD which may accelerate disease progression. Vulnerability to stress and higher levels of anxiety are significantly associated with the incidence of dementia [552]. Environmental and external stress can lead to psychological stress and the subsequent cellular stress exacerbated by inflammation and oxidative damage [553–555]. Psychological stress activates the hypothalamic-pituitary-adrenocortical (HPA) axis, eventually leading to the secretion of glucocorticoid into the bloodstream, where blood glucocorticoid enters the brain through the BBB to activate the glucocorticoid receptor (GR in human) and mineral corticosteroid receptor (MR in mice) [556, 557]. Chronic stress causes long-term activation of the HPA axis [558], accompanied by permanent depletion of receptors and loss of hippocampal neurons [559]. The glucocorticoid cascade hypothesis suggests that HPA axis dysfunction may be a sensitizing factor in the pathogenesis of AD and other neurodegenerative diseases.

### Lifestyle habits

#### Sleep

Sleep deprivation (SD) is a common health concern in older people. Mounting evidence suggests that sleep disorders increase AD risk [560–562], and about 15% of AD cases may be attributed to sleep problems [560]. Sleep disorders may manifest at early stages of AD onset, but seem to correlate more severely with cognitive decline [563, 564]. The origin of SD in AD is unclear but is considered to involve multiple factors.

SD may exacerbate cognitive deficits in AD through impairment of sleep-dependent memory consolidation [565, 566]. In addition, SD can affect both Aβ and tau metabolism. SD is associated with fluctuations in CSF Aβ, as well as Aβ deposition in the brain [567]. In humans, SD increases CSF levels of Aβ38, Aβ40, and Aβ42 through the enhancement of Aβ production [568, 569]. In animal models of AD, chronic mild sleep restriction aggravates contextual memory impairment, cortical Aβ accumulation and tau hyperphosphorylation [570, 571] A recent study shows that tau levels in mouse ISF and human CSF are higher during normal wakefulness versus sleep. Chronic SD increases pathological tau spreading in mice. In addition, chemogenetic induction of wake states in mice significantly increases both ISF Aβ and tau [420]. Aβ clearance is thought to be enhanced during sleep [572], although how SD regulates Aβ and tau metabolism remains unclear [419]. Using advanced neuroimaging, a recent study reveals that waves of CSF flow appear during sleep in human brain. It is possible that tau and Aβ clearance is enhanced by CSF circulation, which may be impaired by SD [573]. In contrast to SD, enhancing normal sleep patterns may alleviate AD pathogenesis, as extension of sleep duration decreases plaque deposition in animal models [574]. Therefore, establishing and maintaining normal sleep patterns and remediating SD may reduce AD risk.

#### The gut microbiota

The human gut microbiota comprises approximately 10^14^ microbes [575], which is 10 times greater than the number of non-microbial cells that make up the human body [576]. Gut microbiota play a crucial role in maintaining human health. Specifically, gut microbes synthesize and release a number of functional co-enzymes and nutrients including folates, biotin, B vitamins, amino acids and other factors [577]. Human gut microbiota can also form a protective barrier which inhibits colonization of pathogenic bacteria and inhibits pathogens from adhering to intestinal cells [578]. Recent studies have investigated the impact of the gut microbiota on brain function and neurodegenerative diseases such as AD and PD.

5-HT (serotonin) levels in blood from Germ-free (GF) mice are decreased compared to mice with normal gut microbiota; where serotonin levels are restored with reconstitution of the gut microbiome [579]. As serotonin can reduce Aβ plaque formation and AD risk [580], GF conditions may modulate Aβ pathology through alterations in serotonin. In addition, expression of NMDAR and BDNF is significantly reduced in GF mice [572, 581, 582], suggesting that microbiota may affect brain function through these components in the CNS.

Broad-spectrum antibiotics can reduce the abundance and diversity of gut microbiota, leading to an imbalanced microbiome (dysbiosis). Dysbiosis in weaned rats leads to impaired spatial memory and reduced NMDAR and BDNF levels in the brain [582, 583]. In addition, long-term use of antibiotics in adult mice reduces neuronal regeneration in the hippocampus and impairs cognitive function [584]. However, antibiotics can reduce Aβ deposition and plaque-associated glial reactivity in an AD mouse model [585]. Therefore, further study may be required to elucidate the effect of different antibiotics on AD.

Intestinal bacteria can regulate brain function through the production of toxins and metabolites. For instance, cyanobacteria can produce neurotoxic-N-methylamino-L-alanine, saxitoxin and anatoxin. These toxins have been shown to exacerbate AD pathology [586, 587]. However, not all intestinal bacteria are deleterious; probiotic microbial species such as *Lactobacillus brevis* and *Bifidobacterium dentium* can produce GABA (the major inhibitory neurotransmitter) to maintain normal brain function [588].

Abnormal gut microbiota has been reported in several AD mouse models [589, 590]. For example, gut microbiota from APP/PS1 mice differs significantly compared to wild-type mice. The abundance of microbes such as *Helicobacteraceae*, *Desulfovibrionaceae, Odoribacter* and *Helicobacter* is elevated in APP/PS1 transgenic (Tg) animals, whereas microbes such as *Firmicutes*, *Verrucomicrobia*, *Proteobacteria*, *Actinobacteria* are reduced in Tg animals [591]. Interestingly, learning and memory in Tg animals can be improved through probiotic transplantation of microbiota from young control wild-type mice to Tg animals. Microbial transplantation also restores deficits in synaptic plasticity in Tg mice and decreases levels of phosphorylated tau, Aβ40 and Aβ42 [592]. In ADLP^APT^ transgenic mice which develop both amyloid and tau pathologies, transplantation of fecal microbes from WT mice into ADLP^APT^ mice ameliorates Aβ plaque and neurofibrillary tangle formation, glial reactivity and cognitive impairment [593]. Thus, fecal microbial transplantation may be a potential therapeutic strategy for AD.

#### High dietary fat and sodium

Obesity and AD both have an alarmingly high prevalence in Western society [594]. High fat diet is thought to contribute directly to several key aspects of AD, including increased accumulation of Aβ, tau hyperphosphorylation, and inflammation of peripheral organs and brain [595–597]. High dietary salt is a risk factor for dementia [598, 599]. A very recent study shows that high dietary salt leads to cognitive dysfunction of mice in a manner dependent on tau. Mechanistically, high salt intake induces tau hyperphosphorylation through the activation of calpain and CDK5, which may be a result of nitric oxide deficiency [600]. Therefore, high salt diet may increase risk for the onset of AD and other tauopathies.

### CAA/stroke/vascular defects

Cerebral amyloid angiopathy(CAA)is a common cerebrovascular disorder which is characterized by the deposition of amyloid proteins such as Aβ in cerebral vessels. CAA is not only a factor leading to stroke (namely cerebral hemorrhage and ischemic brain lesions), but also an important risk factor for dementia [601]. In AD, the prevalence of CAA can reach as high as 80–90% [602], likely as a result in elevated pathological levels of Aβ. Advanced stage CAA in AD patients aggravates cognitive decline and enhances odds of triggering dementia onset [603]. Cerebrovascular dysfunction is one of the earliest abnormalities detected in CAA, which also manifests in early stages of AD onset [604]. In addition, brain atrophy is a pathological feature common to both disorders [605]. It has been suggested that vascular damage associated with CAA disrupts vascular drainage and homeostasis in the CNS, thereby impairing Aβ clearance and aggravating AD pathogenesis [606]. Furthermore, dysregulation of astrocytic water channels such as AQP4 in CAA may contribute to AD pathogenesis [607]. AQP4 levels are reduced in CAA; since AQP4 plays a vital role in glymphatic Aβ clearance in AD brain parenchyma, this consequently impairs Aβ clearance [606, 608]. Indeed, dysfunction of astrocytic water and potassium channels is observed in AD patient brain and AD mouse models.

In addition, some genetic risk factors are common to both CAA and AD. For example, increased *APP* gene dosage is associated with CAA and Down syndrome-AD [609]. Several *PSEN1* mutations are associated with AD and correlates with pathological CAA severity [610]. Furthermore, APOE is the most potent risk factor for sporadic CAA and AD onset [611, 612].

### Others factors

In addition, there are other factors that affect the incidence of AD, including non-coding RNAs, blood brain barrier, high systolic blood pressure, education and gender. Non-coding RNAs including microRNAs (miRNAs), long non-coding RNAs (lncRNAs), and circular RNAs (circRNAs) may be involved in AD pathogenesis. miRNAs are required to regulate gene expression and enact phenotypic changes in human diseases. Most miRNAs characterized so far are derived from human brain. Many of these miRNAs are responsible for maintaining normal synaptic formation and function, neurotransmitter release, and neurite growth. Alterations in miRNA levels have been observed in AD and other neurodegenerative disorders [613]. miRNAs may participate AD pathogenesis by modulating amyloidogenic pathways: for example, miR-346 can up-regulate APP translation and Aβ production [614]. In addition, miRNAs can activate PPAR-γ, thereby stimulating NF-κB pathways to induce cytokine release and consequent Aβ production [615]. However, how a particular neurodegenerative environment within pathological contexts can alter miRNA levels, and consequential effects in modulating the miRNA milieu requires further elucidation. Levels of several lncRNAs species are increased in the brain of LOAD patients [616]. LncRNAs may accelerate AD progression through various mechanisms. The lncRNA BC1 can induce spatial learning and memory impairment by enhancing APP translation in AD mouse brain [617]. The lncRNA EBF3-AS is upregulated in the brain of AD mice, and downregulation of EBF3-AS can reduce Aβ-induced neuronal apoptosis [618]. In addition, the lncRNA SOX21-AS1 is able to induce oxidative stress injury in neurons by up-regulating Wnt signaling in an AD mouse model [619]. CircRNAs primarily act as endogenous anti-complementary miRNA “sponges”. A very recent study has established an association between circRNA expression and AD [620]. AD-associated circRNAs correlate with the expression of several AD-related genes, and may regulate AD-relevant pathways through binding to miRNAs. For instance, *circCDR1-AS* comprises multiple miRNA-7 binding sites. As decreased *circCDR1-AS* levels lead to downregulation of mRNAs targeted by miR-7 [621], and elevated miRNA-7 levels can downregulate the expression of genes involved in Aβ clearance [622], *circCDR1-AS* may affect Aβ metabolism. Nevertheless, the exact roles for circRNAs in AD progression are almost unknown and this is an interesting area that should be explored in future.

The BBB prevents neurotoxic plasma components, pathogens, and blood cells from entering the brain, and regulates the molecular transport of components into and out of the CNS to homeostatically regulate the neuronal extracellular environment [623, 624]. Growing evidence indicates that BBB damage and early cerebrovascular disease increase risk of dementia and age-related disorders such as AD [625–627]. Damage to the BBB can lead to dysfunctional P-glycoprotein-1-mediated efflux, leading to the accumulation of toxic exogenous substances in the brain. Decreased cerebral blood flow, coupled with increased Aβ levels induced by BBB deterioration can also aggravate tau pathology [628, 629].

Hypertension is an additional AD risk factor: epidemiological studies have shown that hypertension in middle aged individuals (rather than seniors) is associated with an increased risk of AD and dementia. Interestingly, higher education seems to be associated with a protective role of preventing AD onset [630]. Meta-analyses indicate that people with a higher education, occupation of high societal status, and increased intelligence or IQ feature a decreased risk of AD onset [631]. This suggests that education and intellectual function may possibly confer resistance to pathological changes associated with AD [632]. Women are at a higher risk for developing AD compared to men. The effects of gender on AD risk have been attributed to various factors including hormone levels, gene expression, and brain development [633]. Some evidence suggests that early menopause induced by oophorectomy are AD risk factors specific to women [513]. In addition, a higher life expectancy in women may also contribute to higher AD incidence observed in females.

## Lessons from the clinic

So far, the FDA has only approved five drugs for AD. Significantly, these drugs merely modulate AD symptoms; no drugs have been shown to effectively prevent or stop AD progression. Moreover, effects of these drugs gradually dissipate over time, ultimately losing their efficacy. No new drugs have been approved by the FDA for AD since 2003. Recent efforts during the last few years have seen a surge in the development in new AD drugs in both academia and industry. While recent failures in phase 3 clinical trials by Merck, Pfizer, J&J, Eli Lilly and Roche have been rather discouraging, the most probable explanation for these failures may be derived from the inadequacy of animal models used, initiation of treatment at late/irreversible stages during the course of AD development, complications arising from drug dosage, and targeting ineffective targets. These factors are due in large part to an incomplete understanding of complexities in AD pathophysiology [634].

Most AD models comprise transgenic mutants associated with familial early-onset AD, which may not be ideal for sporadic AD research. Given that more than 95% of all AD cases are sporadic [635], it is necessary, albeit difficult, to develop non-familial AD models may be more relevant to human AD pathogenesis. Recent studies have indicated that adult rhesus monkeys can effectively model AD-related neurodegeneration in primate brain, and may represent a more appropriate model for AD [636, 637].

Clinical trials targeting Aβ production have seen very little success. For example, clinical trials for drugs targeting γ-secretase, including γ-secretase inhibitors such as Semagacestat (Eli Lilly) and Avagacesta (Bristol-Myers Squibb) programs have been terminated due to a lack in efficacy and/or appearance of severe side effects. Since the γ-secretase complex cleaves physiological substrates other than APP such as Notch, γ-secretase inhibitors may affect other physiological functions in addition to APP processing. Similarly, inadequate efficacy and/or severe side effects in clinical trials with BACE1 inhibitors such as Verubecestat and Atabecestat [638–640] have also been reported. Although inhibition of β-secretase activity is predicted to reduce Aβ production, BACE1 inhibitors were seen to increase cleavage of APP by other secretases, thereby enhancing the production of pathogenic APP metabolites [36]. In addition, BACE1 KO mice exhibit a variety of neurological defects [641–643], indicating that BACE1 mediates many fundamental functions in brain. In addition, conditional BACE1 deletion in neurons feature axon guidance defects [644], suggesting that BACE1 deletion may have additional non-autonomous roles in neurons. Immunotherapeutic approaches targeting Aβ have also been subjected to clinical testing. Aducanumab (Biogen/Eisai) is a human monoclonal antibody targeting amyloid β fibrils and soluble oligomers [645]. Recently, Biogen has announced that higher dosages of Aducanumab show 23% improvement in AD patients in one of two phase 3 trials. BAN2401 is a monoclonal antibody targeting large and soluble Aβ protofibrils. Phase 2 clinical results for BAN2401 indicate that it can remove Aβ and slow cognitive decline. Phase 3 clinical trials are currently ongoing to evaluate the efficacy of BAN2401 in patients with mild AD. In these trials, BAN2401 is being tested in asymptomatic individuals with Aβ plaques in very early stages of disease onset [646, 647]. Considering failures in previous clinical trials, treatment at this stage may also be too late to show adequate efficacy, as neurodegenerative synaptic damage and neuronal loss are irreversible pathogenic events [648]. Treatments that can be administered at earlier stages of onset should be considered in future studies.

Tau is also a critical therapeutic target in AD. Methylene blue and its derivatives have been tested for its ability to inhibit tau accumulation, but have yet to produce positive effects in clinical trials. A possible explanation for this may be derived from effects of methylene blue on increasing granular tau oligomers which may trigger neuronal death, despite concurrent effects in reducing tau fibril formation [649]. Moreover, use of tau kinase inhibitors such as the GSK-3 inhibitor Tideglusib to attenuate pathological tau hyperphosphorylation also showed little or no efficacy in Phase 2 trials, probably due to the critical role of GSK-3 in multifunctional signaling pathways [650, 651]. Despite these failures, hope lingers in targeting tau in the clinic, with trials currently testing the efficacy of tau-targeting monoclonal antibodies in clinical trials [652].

## Conclusions

**Fig. 1:**
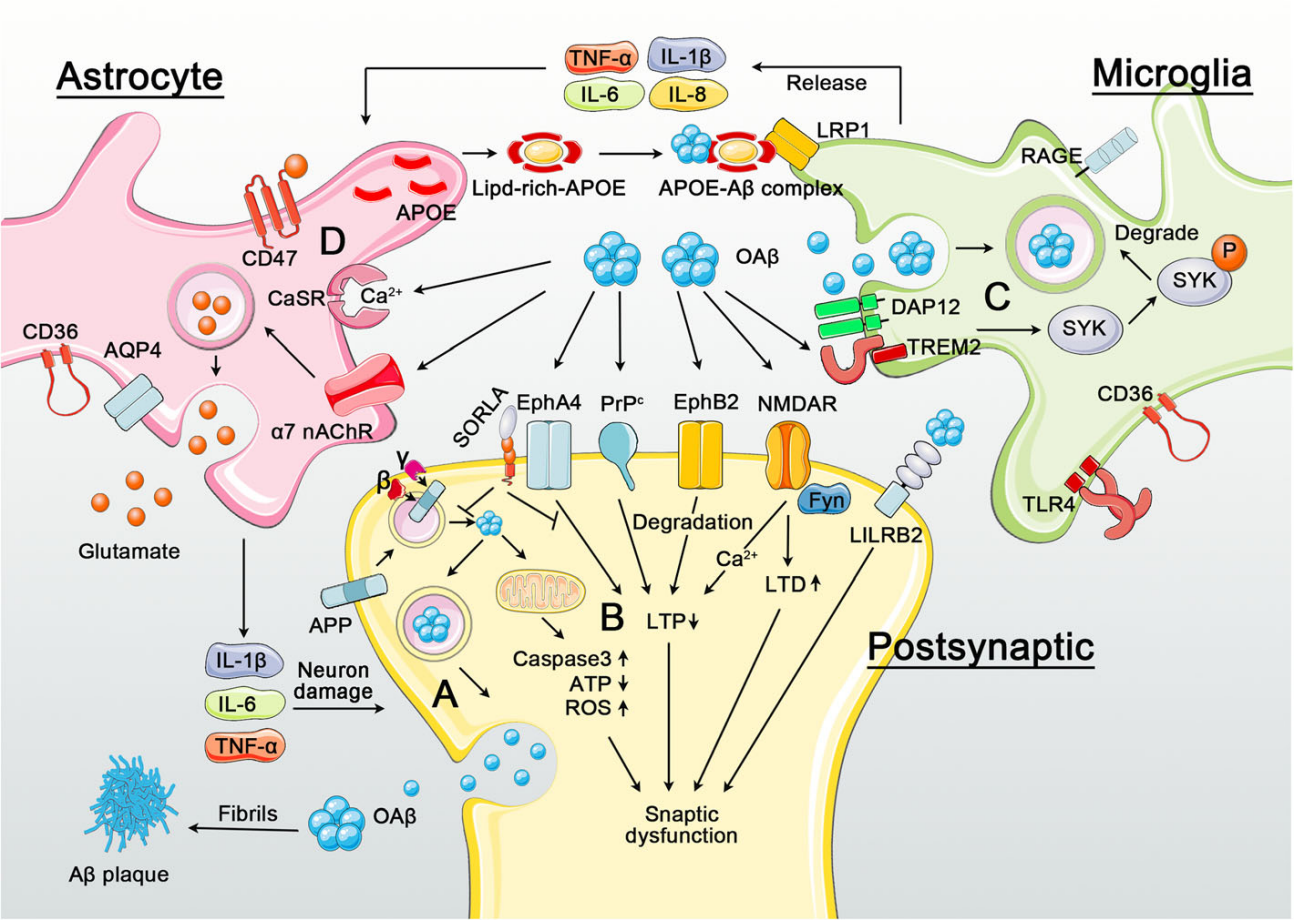
A model for Aβ-induced neurotoxicity and glial response in AD. **a** APP processing and Aβ generation. Aβ is generated by APP cleavage in acidified compartments such as late endosomes, and subsequently released from neurons. Extracellular Aβ sequentially assemble into Aβ oligomer aggregates (oAβ), fibrils, and ultimately amyloid plaques. **b** Aβ-mediated neuronal dysfunction. oAβ can disrupt synaptic function through LTP impairment and LTD enhancement. A variety of potential neuronal Aβ receptors such as EphA4, PrPc, EphB2, NMDAR, and LiLRB2 have been shown to bind Aβ and transduce synaptotoxicity. SORLA can inhibit EphA4-mediated synaptic and cognitive dysfunction with oAβ exposure. Fyn kinase is an important regulator for NMDAR-mediated oAβ neurotoxicity. oAβ also can alter mitochondria function to induce caspase-3 activation, ATP reduction and ROS upregulation, thereby aggravating synaptic dysfunction. **c** Effects of Aβ on microglia. oAβ may activate microglia through binding to the putative Aβ receptors such as TREM2, LRP1, RAGE, TLR4 and CD36. Specifically, the binding of Aβ to TREM2 activates SYK pathway through DAP12, an adaptor protein for TREM2, and leads to the degradation of Aβ. **d** Aβ-dependent microglia/astrocyte interactions, and Aβ-mediated astrocyte dysfunction. APOE released from the astrocytes binds Aβ, which enhances Aβ/APOE interactions with LRP1. Activated microglia release proinflammatory such as TNF-α, IL-1β, IL-6 and IL-8, which can activate astrocytes. In addition, oAβ can potentially activate astrocytes directly through α7-nAchR, CaSR, CD36, CD47 and AQP4. Activated astrocytes may damage neurons through extracellular glutamate dyshomeostasis/excitotoxicity, TNF-α, IL-1β and IL-6

**Fig. 2:**
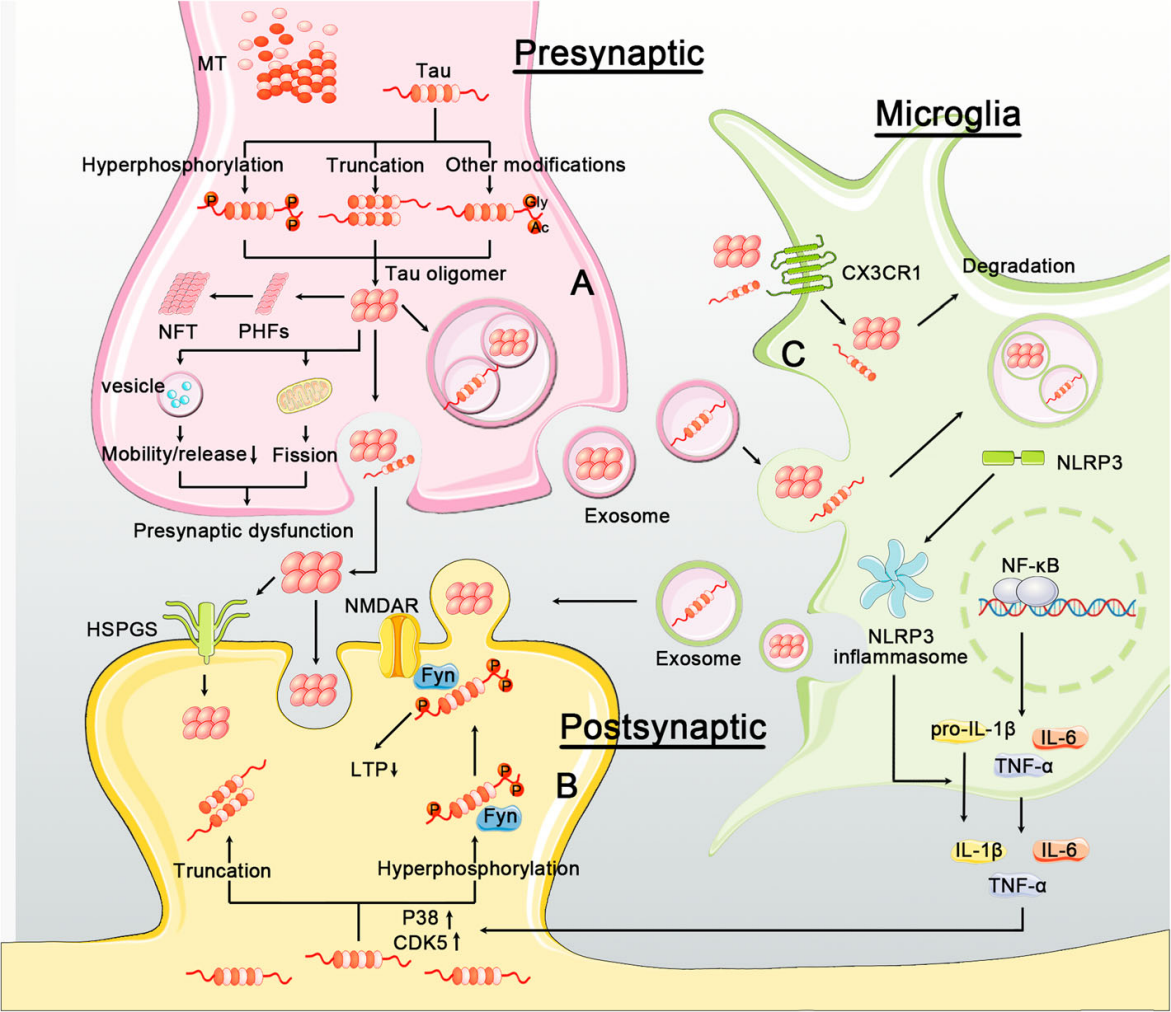
A model for tau pathogenesis. **a** Tau is a microtubule-binding protein, which can undergo various types of post-translational modifications (PTMs), such as phosphorylation and truncation. Under disease conditions, aberrant PTMs induces tau dissociation from microtubules, leading to tau aggregation and oligomer formation. Tau oligomers can further aggregate to form PHFs and NFTs in neurons. Tau aggregates can induce mitochondria fragmentation, impair synaptic vesicle mobility and release, thereby leading to presynaptic dysfunction. In addition, pathological tau species such as truncated tau and tau oligomers can be released to the extracellular environment via exosomes or directly from the plasma membrane. **b** Tau is normally distributed to compartments other than postsynaptic densities. Hyperphosphorylated and truncated tau species may enter postsynaptic compartments to consequently impair LTP by modulating Fyn/NMDAR complexes. Extracellular pathogenic tau species may be internalized in neurons through a HSPGs-mediated pathway to induce the aggregation of intracellular tau. **c** Extracellular tau can bind CX3CR1 receptors, and subsequently internalized by microglia for degradation. Alternatively, tau released from neurons can enter microglia through unknown mechanisms. Internalized tau may be modified and re-released from microglia to the extracellular space via exosomes, and then taken up by adjacent neurons to induce tau propagation. In addition, pathological tau species can activate microglial NF-κB and NLRP3 inflammasome pathways, leading to pro-inflammatory cytokine release. Excessive pro-inflammatory cytokines can increase the activity of tau kinases such as CDK5 and P38, thereby exacerbating tau hyperphosphorylation

AD pathogenesis involves pathogenic contributions from multiple components and alterations in behavior of various cell types within the CNS. Aβ is generated in neurons and then released to the extracellular space, where it can be degraded or cleared by microglia and astrocytes. Increased Aβ production or impaired Aβ degradation/clearance leads to Aβ accumulation. Tau is mainly expressed in neurons, and highly modulated through various PTMs. Abnormal PTMs, LLPS, and pathogenic tau seeds can cause tau aggregation and accumulation through different mechanisms. Tau pathology may be propagated during disease progression, and glial cells play an important role in the process of seeding and dispersion. oAβ and other forms of Aβ aggregates, together with tau accumulation can cause neuronal dysfunction and glial activation and the subsequent neuroinflammation; these events are regulated by various receptors expressed in neurons, microglia and astrocytes (Figs. 1 and 2).

Genetic factors can cause or affect AD pathogenesis. Early-onset AD is mainly due to mutations in APP and PS1/2, which are involved in Aβ generation, while late-onset AD is largely associated with a group of genes enriched in glial cells, such as APOE and TREM2, which are important for Aβ clearance and glial function. Therefore, differential mechanisms may be involved in different forms of AD. In addition, other factors such as aging, metal ion, virus, and microbiota may also contribute to AD pathogenesis via various mechanisms. Despite much knowledge that we have gained, no effective treatment strategies for AD have been successfully developed. Intervention for early-onset AD may require treatment at a young age, as Aβ aggregation and accumulation manifests early onset forms of the disease. Importantly, there are no drugs targeting Aβ that have been proven safe for clinical treatment for youths. Mechanisms for late-onset/sporadic AD are complex and subtypes of late-onset AD may exist. However, most of the available AD animal models carrying early-onset AD-associated mutations can only mimic early-onset AD. Development of animal models to recapitulate pathogenesis of late-onset AD may be beneficial to compare early and late stage forms of AD. This may uncover mechanisms specific to late-onset AD which represents over 90% of AD cases, and potentially provide new insights to therapeutic targets for treatment.

## Data Availability

Not applicable.

## Abbreviations

AD: Alzheimer’s disease
APOE: Apolipoprotein E
Aβ: β-Amyloid
APP: Amyloid precursor protein
KCC2: K-Cl cotransporter 2
GABA: γ-aminobutyric acid
sAPP: Soluble amyloid precursor proteins
CNS: Central nervous system
PS1/2: Presenilins 1/2
CSF: Cerebral spinal fluid
LRP1: Low-density lipoprotein receptor-related protein 1
SorLA: Sortilin-Related Receptor Containing LDLR A Repeats
SNX: Sorting nexin
GGA3: Golgi-localized, γ-ear-containing clathrin adaptor ARF binding protein 3
LTP: Long-term potentiation
LTD: Long-term depression
ROS: Reactive oxygen species
NMDAR: N-methyl-D-aspartic acid receptor
EphB2: Ephrin type-B receptor 2 B2
EphA4: Ephrin type-A receptor 4
PrPc: Cellular prion protein
Lilrb2: Leukocyte immunoglobulin-like receptor B2
PirB: Paired immunoglobulin-like receptor B
PTMs: Post-translational modifications
PHFs: Paired helical filaments
CDK5: Cyclin-dependent kinase 5
GSK3β: Glycogen synthase kinase 3β
PKA: Protein kinase A
CHIP: Hsc70-interacting protein
TRAF6: TNF receptor-associated factor 6
SUMO: Small ubiquitin-like modifier
AEP: Asparagine endopeptidase
PSP: Progressive supranuclear palsy
LLPS: Liquid-liquid phase separation
EC: Entorhinal cortex
HSPGs: Heparan sulfate proteoglycans
PSD95: Postsynaptic density-95
TREM2: Triggering receptor expressed on myeloid cells-2
IL-1β: Interleukin-1β
TNFα: Tumor necrosis factor-α
DAM: Disease-associated microglia
LPS: Lipopolysaccharide
TLR: Toll-like receptor
RAGE: Advanced glycation end product receptor
nAChRs: Nicotinic acetylcholine receptor
α7nAChRs: α 7 subtype of nAChR
CaSR: Calcium-sensing receptor
AQP4: Aquaporin-4
ISF: Interstitial fluid
CX3CR1: Chemokine CX3C receptor1
MCI: Mild cognitive impairment
HDACs: Histone deacetylases
HSV: Herpes simplex virus
HHV: Human herpesvirus
HPA: Hypothalamic-pituitary-adrenocortical
GF: Germ-free
miRNAs: MicroRNAs
LncRNAs: Long non-coding RNAs
CircRNAs: Circular RNAs
LOAD: Late-onset Alzheimer’s disease
BBB: Blood-brain barrier
oAβ: oligomeric Aβ
SV: Synaptic vesicle
SD: Sleep deprivation
CAA: Cerebral amyloid angiopathy

## References

[1] Alzheimer's A (2016). 2016 Alzheimer’s disease facts and figures. Alzheimers Dement.

[2] C., P., World Alzheimer Report 2018 (2018). The state of the art of dementia research: new frontiers.

[3] Prince MJ, WA, Guerchet M, Ali G-C, Wu Y-T, Prina M (2015). World Alzheimer report 2015: the global impact of dementia: an analysis of prevalence, Incidence, Cost and Trends.

[4] Tarawneh R, Holtzman DM (2012). The clinical problem of symptomatic Alzheimer disease and mild cognitive impairment. Cold Spring Harb Perspect Med.

[5] Zubenko GS (2003). A collaborative study of the emergence and clinical features of the major depressive syndrome of Alzheimer's disease. Am J Psychiatry.

[6] Kalia M (2003). Dysphagia and aspiration pneumonia in patients with Alzheimer's disease. Metabolism.

[7] Lauter H (1968). On the clinical study and psychopathology of Alzheimer's disease. Demonstration of 203 pathologically-anatomically verified cases. Psychiatr Clin (Basel).

[8] Terry RD (1991). Physical basis of cognitive alterations in Alzheimer's disease: synapse loss is the major correlate of cognitive impairment. Ann Neurol.

[9] Iqbal K, Liu F, Gong CX (2016). Tau and neurodegenerative disease: the story so far. Nat Rev Neurol.

[10] Itagaki S (1989). Relationship of microglia and astrocytes to amyloid deposits of Alzheimer disease. J Neuroimmunol.

[11] Iqbal K, Grundke-Iqbal I (2002). Neurofibrillary pathology leads to synaptic loss and not the other way around in Alzheimer disease. J Alzheimers Dis.

[12] Petrella C (2019). Neuropeptides in Alzheimer's disease: an update. Curr Alzheimer Res.

[13] Katsumoto A (2018). Microglia in Alzheimer’s disease: risk factors and inflammation. Front Neurol.

[14] Dansokho C, Heneka MT (2018). Neuroinflammatory responses in Alzheimer’s disease. J Neural Transm (Vienna).

[15] Tonnies E, Trushina E (2017). Oxidative stress, synaptic dysfunction, and Alzheimer’s disease. J Alzheimers Dis.

[16] Simunkova M (2019). Management of oxidative stress and other pathologies in Alzheimer’s disease. Arch Toxicol.

[17] O'Brien RJ, Wong PC (2011). Amyloid Precursor Protein Processing and Alzheimer’s Disease. Annu Rev Neurosci.

[18] Matsui T (2007). Expression of APP pathway mRNAs and proteins in Alzheimer’s disease. Brain Res.

[19] Ludewig S, Korte M (2016). Novel insights into the physiological function of the APP (gene) family and its proteolytic fragments in synaptic plasticity. Front Mol Neurosci.

[20] Chen M, et al. APP modulates KCC2 expression and function in hippocampal GABAergic inhibition. Elife. 2017;6:e20142. 10.7554/eLife.20142 PMC5224924 28054918

[21] Tackenberg C, Nitsch RM (2019). The secreted APP ectodomain sAPPalpha, but not sAPPbeta, protects neurons against Abeta oligomer-induced dendritic spine loss and increased tau phosphorylation. Mol Brain.

[22] Caille I (2004). Soluble form of amyloid precursor protein regulates proliferation of progenitors in the adult subventricular zone. Development.

[23] Ohsawa I (1999). Amino-terminal region of secreted form of amyloid precursor protein stimulates proliferation of neural stem cells. Eur J Neurosci.

[24] Han P (2005). Suppression of cyclin-dependent kinase 5 activation by amyloid precursor protein: a novel excitoprotective mechanism involving modulation of tau phosphorylation. J Neurosci.

[25] Ring S (2007). The secreted beta-amyloid precursor protein ectodomain APPs alpha is sufficient to rescue the anatomical, behavioral, and electrophysiological abnormalities of APP-deficient mice. J Neurosci.

[26] Nikolaev A (2009). APP binds DR6 to trigger axon pruning and neuron death via distinct caspases. Nature.

[27] Zhang YW (2011). APP processing in Alzheimer’s disease. Mol Brain.

[28] Bibl M (2006). CSF amyloid-beta-peptides in Alzheimer’s disease, dementia with Lewy bodies and Parkinson's disease dementia. Brain.

[29] Bibl M (2007). Validation of amyloid-beta peptides in CSF diagnosis of neurodegenerative dementias. Mol Psychiatry.

[30] Iwatsubo T (1994). Visualization of A beta 42(43) and A beta 40 in senile plaques with end-specific A beta monoclonals: evidence that an initially deposited species is A beta 42(43). Neuron.

[31] Jan A (2008). The ratio of monomeric to aggregated forms of Abeta40 and Abeta42 is an important determinant of amyloid-beta aggregation, fibrillogenesis, and toxicity. J Biol Chem.

[32] Welge V (2009). Combined CSF tau, p-tau181 and amyloid-beta 38/40/42 for diagnosing Alzheimer’s disease. J Neural Transm (Vienna).

[33] Tang W (2014). Assessment of CSF Abeta42 as an aid to discriminating Alzheimer’s disease from other dementias and mild cognitive impairment: a meta-analysis of 50 studies. J Neurol Sci.

[34] Mulugeta E (2011). CSF amyloid beta38 as a novel diagnostic marker for dementia with Lewy bodies. J Neurol Neurosurg Psychiatry.

[35] Sekine-Aizawa Y (2001). Matrix metalloproteinase (MMP) system in brain: identification and characterization of brain-specific MMP highly expressed in cerebellum. Eur J Neurosci.

[36] Willem M (2015). Eta-secretase processing of APP inhibits neuronal activity in the hippocampus. Nature.

[37] Lanoiselee HM (2017). APP, PSEN1, and PSEN2 mutations in early-onset Alzheimer disease: A genetic screening study of familial and sporadic cases. PLoS Med.

[38] De Jonghe C (2001). Pathogenic APP mutations near the gamma-secretase cleavage site differentially affect Abeta secretion and APP C-terminal fragment stability. Hum Mol Genet.

[39] Haass C (1995). The Swedish mutation causes early-onset Alzheimer’s disease by beta-secretase cleavage within the secretory pathway. Nat Med.

[40] Jonsson T (2012). A mutation in APP protects against Alzheimer’s disease and age-related cognitive decline. Nature.

[41] Eggert S (2018). Trafficking in Alzheimer’s disease: modulation of APP transport and processing by the transmembrane proteins LRP1, SorLA, SorCS1c, Sortilin, and Calsyntenin. Mol Neurobiol.

[42] Hou HY (2017). Low-density lipoprotein receptor-related Protein-1 (LRP1) C4408R mutant promotes amyloid precursor protein (APP) alpha-cleavage in vitro. NeuroMolecular Med.

[43] Kang DE (2000). Modulation of amyloid beta-protein clearance and Alzheimer’s disease susceptibility by the LDL receptor-related protein pathway. J Clin Invest.

[44] Andersen OM (2006). Molecular dissection of the interaction between amyloid precursor protein and its neuronal trafficking receptor SorLA/LR11. Biochemistry.

[45] Okada H (2010). Proteomic identification of sorting nexin 6 as a negative regulator of BACE1-mediated APP processing. FASEB J.

[46] Zhao Y (2012). Sorting nexin 12 interacts with BACE1 and regulates BACE1-mediated APP processing. Mol Neurodegener.

[47] Huang TY (2016). SNX27 and SORLA interact to reduce Amyloidogenic subcellular distribution and processing of amyloid precursor protein. J Neurosci.

[48] Wang X (2014). Sorting nexin 27 regulates Abeta production through modulating gamma-secretase activity. Cell Rep.

[49] Kim W (2018). BACE1 elevation engendered by GGA3 deletion increases beta-amyloid pathology in association with APP elevation and decreased CHL1 processing in 5XFAD mice. Mol Neurodegener.

[50] Kang EL (2010). Ubiquitin regulates GGA3-mediated degradation of BACE1. J Biol Chem.

[51] Tesco G (2007). Depletion of GGA3 stabilizes BACE and enhances beta-secretase activity. Neuron.

[52] Walsh DM, Selkoe DJ (2007). A beta oligomers - a decade of discovery. J Neurochem.

[53] Aleksis R (2017). Structural studies of amyloid-beta peptides: unlocking the mechanism of aggregation and the associated toxicity. Biochimie.

[54] Sengupta U, Nilson AN, Kayed R (2016). The role of amyloid-beta oligomers in toxicity, propagation, and immunotherapy. Ebiomedicine.

[55] Benilova I, De Strooper B (2013). Neuroscience. Promiscuous Alzheimer’s amyloid: yet another partner. Science.

[56] Cull-Candy S, Brickley S, Farrant M (2001). NMDA receptor subunits: diversity, development and disease. Curr Opin Neurobiol.

[57] Kohr G (2006). NMDA receptor function: subunit composition versus spatial distribution. Cell Tissue Res.

[58] Andreoli V (2014). Potential involvement of GRIN2B encoding the NMDA receptor subunit NR2B in the spectrum of Alzheimer’s disease. J Neural Transm (Vienna).

[59] Liu HP (2009). Genetic variation in N-methyl-D-aspartate receptor subunit NR3A but not NR3B influences susceptibility to Alzheimer’s disease. Dement Geriatr Cogn Disord.

[60] Jacob CP (2007). Alterations in expression of glutamatergic transporters and receptors in sporadic Alzheimer’s disease. J Alzheimers Dis.

[61] Hynd MR, Scott HL, Dodd PR (2001). Glutamate(NMDA) receptor NR1 subunit mRNA expression in Alzheimer’s disease. J Neurochem.

[62] Mishizen-Eberz AJ (2004). Biochemical and molecular studies of NMDA receptor subunits NR1/2A/2B in hippocampal subregions throughout progression of Alzheimer’s disease pathology. Neurobiol Dis.

[63] De Felice FG (2007). Abeta oligomers induce neuronal oxidative stress through an N-methyl-D-aspartate receptor-dependent mechanism that is blocked by the Alzheimer drug memantine. J Biol Chem.

[64] Parameshwaran K, Dhanasekaran M, Suppiramaniam V (2008). Amyloid beta peptides and glutamatergic synaptic dysregulation. Exp Neurol.

[65] Danysz W, Parsons CG (2012). Alzheimer’s disease, beta-amyloid, glutamate, NMDA receptors and memantine--searching for the connections. Br J Pharmacol.

[66] Li S (2009). Soluble oligomers of amyloid Beta protein facilitate hippocampal long-term depression by disrupting neuronal glutamate uptake. Neuron.

[67] Shankar GM (2008). Amyloid-beta protein dimers isolated directly from Alzheimer’s brains impair synaptic plasticity and memory. Nat Med.

[68] Snyder EM (2005). Regulation of NMDA receptor trafficking by amyloid-beta. Nat Neurosci.

[69] Zhang Y (2016). Dysfunction of NMDA receptors in Alzheimer’s disease. Neurol Sci.

[70] Danysz W, Parsons CG (2003). The NMDA receptor antagonist memantine as a symptomatological and neuroprotective treatment for Alzheimer’s disease: preclinical evidence. Int J Geriatr Psychiatry.

[71] Kullander K, Klein R (2002). Mechanisms and functions of Eph and ephrin signalling. Nat Rev Mol Cell Biol.

[72] Poliakov A, Cotrina M, Wilkinson DG (2004). Diverse roles of eph receptors and ephrins in the regulation of cell migration and tissue assembly. Dev Cell.

[73] Yamaguchi Y, Pasquale EB (2004). Eph receptors in the adult brain. Curr Opin Neurobiol.

[74] Attwood BK, Patel S, Pawlak R (2012). Ephs and ephrins: emerging therapeutic targets in neuropathology. Int J Biochem Cell Biol.

[75] Lacor PN (2007). Abeta oligomer-induced aberrations in synapse composition, shape, and density provide a molecular basis for loss of connectivity in Alzheimer’s disease. J Neurosci.

[76] Cisse M (2011). Reversing EphB2 depletion rescues cognitive functions in Alzheimer model. Nature.

[77] Miyamoto T (2016). Increasing the receptor tyrosine kinase EphB2 prevents amyloid-beta-induced depletion of cell surface glutamate receptors by a mechanism that requires the PDZ-binding motif of EphB2 and neuronal activity. J Biol Chem.

[78] Murai KK (2003). Control of hippocampal dendritic spine morphology through ephrin-A3/EphA4 signaling. Nat Neurosci.

[79] Rosenberger AF (2014). Altered distribution of the EphA4 kinase in hippocampal brain tissue of patients with Alzheimer’s disease correlates with pathology. Acta Neuropathol Commun.

[80] Huang TY (2017). SORLA attenuates EphA4 signaling and amyloid beta-induced neurodegeneration. J Exp Med.

[81] Fu AK (2014). Blockade of EphA4 signaling ameliorates hippocampal synaptic dysfunctions in mouse models of Alzheimer’s disease. Proc Natl Acad Sci U S A.

[82] Lamberto I (2012). Distinctive binding of three antagonistic peptides to the ephrin-binding pocket of the EphA4 receptor. Biochem J.

[83] Le Pichon CE (2009). Olfactory behavior and physiology are disrupted in prion protein knockout mice. Nat Neurosci.

[84] Rial D (2012). Overexpression of cellular prion protein (PrP(C)) prevents cognitive dysfunction and apoptotic neuronal cell death induced by amyloid-beta (Abeta(1)(−)(4)(0)) administration in mice. Neuroscience.

[85] Zou WQ (2011). Insoluble cellular prion protein and its association with prion and Alzheimer diseases. Prion.

[86] Linden R (2008). Physiology of the prion protein. Physiol Rev.

[87] Lauren J (2009). Cellular prion protein mediates impairment of synaptic plasticity by amyloid-beta oligomers. Nature.

[88] Kostylev MA (2015). Prion-protein-interacting amyloid-beta oligomers of high molecular weight are tightly correlated with memory impairment in multiple Alzheimer mouse models. J Biol Chem.

[89] Gimbel DA (2010). Memory impairment in transgenic Alzheimer mice requires cellular prion protein. J Neurosci.

[90] Nicoll AJ (2013). Amyloid-beta nanotubes are associated with prion protein-dependent synaptotoxicity. Nat Commun.

[91] Klyubin I (2014). Peripheral administration of a humanized anti-PrP antibody blocks Alzheimer’s disease Abeta synaptotoxicity. J Neurosci.

[92] Zheng J (2012). Inhibitory receptors bind ANGPTLs and support blood stem cells and leukaemia development. Nature.

[93] Kim T (2013). Human LilrB2 is a beta-amyloid receptor and its murine homolog PirB regulates synaptic plasticity in an Alzheimer’s model. Science.

[94] Cao Q (2018). Inhibiting amyloid-beta cytotoxicity through its interaction with the cell surface receptor LilrB2 by structure-based design. Nat Chem.

[95] Abolhassani N (2017). Molecular pathophysiology of impaired glucose metabolism, mitochondrial dysfunction, and oxidative DNA damage in Alzheimer’s disease brain. Mech Ageing Dev.

[96] Ebenezer PJ (2010). Neuron specific toxicity of oligomeric amyloid-beta: role for JUN-kinase and oxidative stress. J Alzheimers Dis.

[97] Wang X (2009). Impaired balance of mitochondrial fission and fusion in Alzheimer’s disease. J Neurosci.

[98] Butterfield DA, Bader Lange ML, Sultana R (2010). Involvements of the lipid peroxidation product, HNE, in the pathogenesis and progression of Alzheimer’s disease. Biochim Biophys Acta.

[99] Wang X (2014). Oxidative stress and mitochondrial dysfunction in Alzheimer’s disease. Biochim Biophys Acta.

[100] Moreira PI (2010). Mitochondrial dysfunction is a trigger of Alzheimer’s disease pathophysiology. Biochim Biophys Acta.

[101] Zhang H (2012). Appoptosin is a novel pro-apoptotic protein and mediates cell death in neurodegeneration. J Neurosci.

[102] Lustbader JW (2004). ABAD directly links Abeta to mitochondrial toxicity in Alzheimer’s disease. Science.

[103] Du H (2008). Cyclophilin D deficiency attenuates mitochondrial and neuronal perturbation and ameliorates learning and memory in Alzheimer’s disease. Nat Med.

[104] Du H (2011). Cyclophilin D deficiency improves mitochondrial function and learning/memory in aging Alzheimer disease mouse model. Neurobiol Aging.

[105] Neve RL (1986). Identification of cDNA clones for the human microtubule-associated protein tau and chromosomal localization of the genes for tau and microtubule-associated protein 2. Brain Res.

[106] Goedert M (1989). Multiple isoforms of human microtubule-associated protein tau: sequences and localization in neurofibrillary tangles of Alzheimer’s disease. Neuron.

[107] Goedert M (1989). Cloning and sequencing of the cDNA encoding an isoform of microtubule-associated protein tau containing four tandem repeats: differential expression of tau protein mRNAs in human brain. EMBO J.

[108] Sergeant N (1997). Different distribution of phosphorylated tau protein isoforms in Alzheimer’s and Pick's diseases. FEBS Lett.

[109] Stanford PM (2000). Progressive supranuclear palsy pathology caused by a novel silent mutation in exon 10 of the tau gene: expansion of the disease phenotype caused by tau gene mutations. Brain.

[110] Stamelou M (2010). Rational therapeutic approaches to progressive supranuclear palsy. Brain.

[111] Chambers CB (1999). Overexpression of four-repeat tau mRNA isoforms in progressive supranuclear palsy but not in Alzheimer’s disease. Ann Neurol.

[112] Liu F, Gong CX (2008). Tau exon 10 alternative splicing and tauopathies. Mol Neurodegener.

[113] Connell JW (2005). Quantitative analysis of tau isoform transcripts in sporadic tauopathies. Brain Res Mol Brain Res.

[114] Umeda Y (2004). Alterations in human tau transcripts correlate with those of neurofilament in sporadic tauopathies. Neurosci Lett.

[115] Yasojima K, McGeer EG, McGeer PL (1999). Tangled areas of Alzheimer brain have upregulated levels of exon 10 containing tau mRNA. Brain Res.

[116] Hyman BT, Augustinack JC, Ingelsson M (2005). Transcriptional and conformational changes of the tau molecule in Alzheimer’s disease. Biochim Biophys Acta.

[117] Conrad C (2007). Single molecule profiling of tau gene expression in Alzheimer’s disease. J Neurochem.

[118] Ginsberg SD (2006). Shift in the ratio of three-repeat tau and four-repeat tau mRNAs in individual cholinergic basal forebrain neurons in mild cognitive impairment and Alzheimer’s disease. J Neurochem.

[119] Weingarten MD (1975). A protein factor essential for microtubule assembly. Proc Natl Acad Sci U S A.

[120] Kadavath H (2015). Tau stabilizes microtubules by binding at the interface between tubulin heterodimers. Proc Natl Acad Sci U S A.

[121] Kellogg EH (2018). Near-atomic model of microtubule-tau interactions. Science.

[122] Lee G, Neve RL, Kosik KS (1989). The microtubule binding domain of tau protein. Neuron.

[123] Goedert M, Jakes R (1990). Expression of separate isoforms of human tau protein: correlation with the tau pattern in brain and effects on tubulin polymerization. EMBO J.

[124] Dixit R (2008). Differential regulation of dynein and kinesin motor proteins by tau. Science.

[125] Kanai Y, Hirokawa N (1995). Sorting mechanisms of tau and MAP2 in neurons: suppressed axonal transit of MAP2 and locally regulated microtubule binding. Neuron.

[126] Hirokawa N (1996). Selective stabilization of tau in axons and microtubule-associated protein 2C in cell bodies and dendrites contributes to polarized localization of cytoskeletal proteins in mature neurons. J Cell Biol.

[127] Vossel KA (2010). Tau reduction prevents Abeta-induced defects in axonal transport. Science.

[128] Ittner LM (2010). Dendritic function of tau mediates amyloid-beta toxicity in Alzheimer’s disease mouse models. Cell.

[129] Frandemiche ML (2014). Activity-dependent tau protein translocation to excitatory synapse is disrupted by exposure to amyloid-beta oligomers. J Neurosci.

[130] Zhao Y (2015). Appoptosin-mediated caspase cleavage of tau contributes to progressive Supranuclear palsy pathogenesis. Neuron.

[131] Ahmed T (2014). Cognition and hippocampal synaptic plasticity in mice with a homozygous tau deletion. Neurobiol Aging.

[132] Kimura T (2014). Microtubule-associated protein tau is essential for long-term depression in the hippocampus. Philos Trans R Soc Lond Ser B Biol Sci.

[133] LoPresti P (1995). Functional implications for the microtubule-associated protein tau: localization in oligodendrocytes. Proc Natl Acad Sci U S A.

[134] Papasozomenos SC, Binder LI (1987). Phosphorylation determines two distinct species of tau in the central nervous system. Cell Motil Cytoskeleton.

[135] Zhang Y (2014). An RNA-sequencing transcriptome and splicing database of glia, neurons, and vascular cells of the cerebral cortex. J Neurosci.

[136] LoPresti P (2002). Regulation and differential expression of tau mRNA isoforms as oligodendrocytes mature in vivo: implications for myelination. Glia.

[137] Seiberlich V (2015). Downregulation of the microtubule associated protein tau impairs process outgrowth and myelin basic protein mRNA transport in oligodendrocytes. Glia.

[138] Klein C (2002). Process outgrowth of oligodendrocytes is promoted by interaction of fyn kinase with the cytoskeletal protein tau. J Neurosci.

[139] Lei P (2012). Tau deficiency induces parkinsonism with dementia by impairing APP-mediated iron export. Nat Med.

[140] Marciniak E (2017). Tau deletion promotes brain insulin resistance. J Exp Med.

[141] Tapia-Rojas C (2019). It’s all about tau. Prog Neurobiol.

[142] Regan P (2015). Tau phosphorylation at serine 396 residue is required for hippocampal LTD. J Neurosci.

[143] Hoover BR (2010). Tau mislocalization to dendritic spines mediates synaptic dysfunction independently of neurodegeneration. Neuron.

[144] Ittner A (2016). Site-specific phosphorylation of tau inhibits amyloid-beta toxicity in Alzheimer’s mice. Science.

[145] Sontag E (1996). Regulation of the phosphorylation state and microtubule-binding activity of tau by protein phosphatase 2A. Neuron.

[146] Drewes G (1993). Dephosphorylation of tau protein and Alzheimer paired helical filaments by calcineurin and phosphatase-2A. FEBS Lett.

[147] Liu F (2005). Contributions of protein phosphatases PP1, PP2A, PP2B and PP5 to the regulation of tau phosphorylation. Eur J Neurosci.

[148] Pei JJ (1997). Distribution, levels, and activity of glycogen synthase kinase-3 in the Alzheimer disease brain. J Neuropathol Exp Neurol.

[149] Tseng HC (2002). A survey of Cdk5 activator p35 and p25 levels in Alzheimer’s disease brains. FEBS Lett.

[150] Vogelsberg-Ragaglia V (2001). PP2A mRNA expression is quantitatively decreased in Alzheimer’s disease hippocampus. Exp Neurol.

[151] Sontag E (2004). Altered expression levels of the protein phosphatase 2A ABalphaC enzyme are associated with Alzheimer disease pathology. J Neuropathol Exp Neurol.

[152] Gong CX (1993). Phosphoprotein phosphatase activities in Alzheimer disease brain. J Neurochem.

[153] Min SW (2010). Acetylation of tau inhibits its degradation and contributes to tauopathy. Neuron.

[154] Cohen TJ (2013). The microtubule-associated tau protein has intrinsic acetyltransferase activity. Nat Struct Mol Biol.

[155] Cook C (2014). Acetylation of the KXGS motifs in tau is a critical determinant in modulation of tau aggregation and clearance. Hum Mol Genet.

[156] Min SW (2015). Critical role of acetylation in tau-mediated neurodegeneration and cognitive deficits. Nat Med.

[157] Tracy TE (2016). Acetylated tau obstructs KIBRA-mediated signaling in synaptic plasticity and promotes Tauopathy-related memory loss. Neuron.

[158] Cohen TJ (2011). The acetylation of tau inhibits its function and promotes pathological tau aggregation. Nat Commun.

[159] Irwin DJ (2012). Acetylated tau, a novel pathological signature in Alzheimer’s disease and other tauopathies. Brain.

[160] Sohn PD (2016). Acetylated tau destabilizes the cytoskeleton in the axon initial segment and is mislocalized to the somatodendritic compartment. Mol Neurodegener.

[161] Morris M (2015). Tau post-translational modifications in wild-type and human amyloid precursor protein transgenic mice. Nat Neurosci.

[162] Gorsky MK (2016). Acetylation mimic of lysine 280 exacerbates human tau neurotoxicity in vivo. Sci Rep.

[163] Morishima-Kawashima M (1993). Ubiquitin is conjugated with amino-terminally processed tau in paired helical filaments. Neuron.

[164] Cripps D (2006). Alzheimer disease-specific conformation of hyperphosphorylated paired helical filament-tau is polyubiquitinated through Lys-48, Lys-11, and Lys-6 ubiquitin conjugation. J Biol Chem.

[165] Paine S (2009). Immunoreactivity to Lys63-linked polyubiquitin is a feature of neurodegeneration. Neurosci Lett.

[166] Keck S (2003). Proteasome inhibition by paired helical filament-tau in brains of patients with Alzheimer’s disease. J Neurochem.

[167] Petrucelli L (2004). CHIP and Hsp70 regulate tau ubiquitination, degradation and aggregation. Hum Mol Genet.

[168] Babu JR, Geetha T, Wooten MW (2005). Sequestosome 1/p62 shuttles polyubiquitinated tau for proteasomal degradation. J Neurochem.

[169] Flach K (2014). Axotrophin/MARCH7 acts as an E3 ubiquitin ligase and ubiquitinates tau protein in vitro impairing microtubule binding. Biochim Biophys Acta.

[170] Peleg S (2010). Altered histone acetylation is associated with age-dependent memory impairment in mice. Science.

[171] Dickey CA (2006). Deletion of the ubiquitin ligase CHIP leads to the accumulation, but not the aggregation, of both endogenous phospho- and caspase-3-cleaved tau species. J Neurosci.

[172] Dorval V, Fraser PE (2006). Small ubiquitin-like modifier (SUMO) modification of natively unfolded proteins tau and alpha-synuclein. J Biol Chem.

[173] Luo HB (2014). SUMOylation at K340 inhibits tau degradation through deregulating its phosphorylation and ubiquitination. Proc Natl Acad Sci U S A.

[174] Funk KE (2014). Lysine methylation is an endogenous post-translational modification of tau protein in human brain and a modulator of aggregation propensity. Biochem J.

[175] Thomas SN (2012). Dual modification of Alzheimer’s disease PHF-tau protein by lysine methylation and ubiquitylation: a mass spectrometry approach. Acta Neuropathol.

[176] Huseby CJ, et al. Quantification of tau protein lysine methylation in aging and Alzheimer’s disease. J Alzheimers Dis. 2019;71(3):979–91. 10.3233/JAD-190604 PMC6844542 31450505

[177] Ledesma MD (1994). Analysis of microtubule-associated protein tau glycation in paired helical filaments. J Biol Chem.

[178] Ledesma MD, Bonay P, Avila J (1995). Tau protein from Alzheimer’s disease patients is glycated at its tubulin-binding domain. J Neurochem.

[179] Yan SD (1994). Glycated tau protein in Alzheimer disease: a mechanism for induction of oxidant stress. Proc Natl Acad Sci U S A.

[180] Yan SD (1995). Non-enzymatically glycated tau in Alzheimer’s disease induces neuronal oxidant stress resulting in cytokine gene expression and release of amyloid beta-peptide. Nat Med.

[181] Necula M, Kuret J (2004). Pseudophosphorylation and glycation of tau protein enhance but do not trigger fibrillization in vitro. J Biol Chem.

[182] Wang JZ, Grundke-Iqbal I, Iqbal K (1996). Glycosylation of microtubule-associated protein tau: an abnormal posttranslational modification in Alzheimer’s disease. Nat Med.

[183] Arnold CS (1996). The microtubule-associated protein tau is extensively modified with O-linked N-acetylglucosamine. J Biol Chem.

[184] Liu F (2002). Role of glycosylation in hyperphosphorylation of tau in Alzheimer’s disease. FEBS Lett.

[185] Losev Y (2019). Novel model of secreted human tau protein reveals the impact of the abnormal N-glycosylation of tau on its aggregation propensity. Sci Rep.

[186] Yuzwa SA (2011). Mapping O-GlcNAc modification sites on tau and generation of a site-specific O-GlcNAc tau antibody. Amino Acids.

[187] Liu F (2009). Reduced O-GlcNAcylation links lower brain glucose metabolism and tau pathology in Alzheimer’s disease. Brain.

[188] Yuzwa SA (2008). A potent mechanism-inspired O-GlcNAcase inhibitor that blocks phosphorylation of tau in vivo. Nat Chem Biol.

[189] Quinn JP (2018). Tau proteolysis in the pathogenesis of Tauopathies: neurotoxic fragments and novel biomarkers. J Alzheimers Dis.

[190] Zhao X (2016). Caspase-2 cleavage of tau reversibly impairs memory. Nat Med.

[191] Gamblin TC (2003). Caspase cleavage of tau: linking amyloid and neurofibrillary tangles in Alzheimer’s disease. Proc Natl Acad Sci U S A.

[192] Rissman RA (2004). Caspase-cleavage of tau is an early event in Alzheimer disease tangle pathology. J Clin Invest.

[193] Guo H (2004). Active caspase-6 and caspase-6-cleaved tau in neuropil threads, neuritic plaques, and neurofibrillary tangles of Alzheimer’s disease. Am J Pathol.

[194] Means JC (2016). Caspase-3-dependent proteolytic cleavage of tau causes neurofibrillary tangles and results in cognitive impairment during Normal aging. Neurochem Res.

[195] Barini E (2016). Metformin promotes tau aggregation and exacerbates abnormal behavior in a mouse model of tauopathy. Mol Neurodegener.

[196] Kim Y (2016). Caspase-cleaved tau exhibits rapid memory impairment associated with tau oligomers in a transgenic mouse model. Neurobiol Dis.

[197] de Calignon A (2010). Caspase activation precedes and leads to tangles. Nature.

[198] Horowitz PM (2004). Early N-terminal changes and caspase-6 cleavage of tau in Alzheimer’s disease. J Neurosci.

[199] Ramcharitar J (2013). Cerebrospinal fluid tau cleaved by caspase-6 reflects brain levels and cognition in aging and Alzheimer disease. J Neuropathol Exp Neurol.

[200] Ono Y, Saido TC, Sorimachi H (2016). Calpain research for drug discovery: challenges and potential. Nat Rev Drug Discov.

[201] Liu J, Liu MC, Wang KK (2008). Calpain in the CNS: from synaptic function to neurotoxicity. Sci Signal.

[202] Johnson GV, Jope RS, Binder LI (1989). Proteolysis of tau by calpain. Biochem Biophys Res Commun.

[203] Yang LS, Ksiezak-Reding H (1995). Calpain-induced proteolysis of normal human tau and tau associated with paired helical filaments. Eur J Biochem.

[204] Park SY, Ferreira A (2005). The generation of a 17 kDa neurotoxic fragment: an alternative mechanism by which tau mediates beta-amyloid-induced neurodegeneration. J Neurosci.

[205] Garg S (2011). Cleavage of tau by calpain in Alzheimer’s disease: the quest for the toxic 17 kD fragment. Neurobiol Aging.

[206] Chen HH (2018). Calpain-mediated tau fragmentation is altered in Alzheimer’s disease progression. Sci Rep.

[207] Matsumoto SE (2015). The twenty-four KDa C-terminal tau fragment increases with aging in tauopathy mice: implications of prion-like properties. Hum Mol Genet.

[208] Ferreira A, Bigio EH (2011). Calpain-mediated tau cleavage: a mechanism leading to neurodegeneration shared by multiple tauopathies. Mol Med.

[209] Lang AE, Riherd Methner DN, Ferreira A (2014). Neuronal degeneration, synaptic defects, and behavioral abnormalities in tau(4)(5)(−)(2)(3)(0) transgenic mice. Neuroscience.

[210] Litersky JM, Johnson GV (1992). Phosphorylation by cAMP-dependent protein kinase inhibits the degradation of tau by calpain. J Biol Chem.

[211] Liu MC (2011). Dual vulnerability of tau to calpains and caspase-3 proteolysis under neurotoxic and neurodegenerative conditions. ASN Neuro.

[212] Zhang Z (2014). Cleavage of tau by asparagine endopeptidase mediates the neurofibrillary pathology in Alzheimer’s disease. Nat Med.

[213] Behrendt A (2019). Asparagine endopeptidase cleaves tau at N167 after uptake into microglia. Neurobiol Dis.

[214] Schlegel K (2019). N368-tau fragments generated by legumain are detected only in trace amount in the insoluble tau aggregates isolated from AD brain. Acta Neuropathol Commun.

[215] Zhang Z (2017). Inhibition of delta-secretase improves cognitive functions in mouse models of Alzheimer’s disease. Nat Commun.

[216] Johnson GV (1997). The tau protein in human cerebrospinal fluid in Alzheimer’s disease consists of proteolytically derived fragments. J Neurochem.

[217] Meredith JE (2013). Characterization of novel CSF tau and ptau biomarkers for Alzheimer’s disease. PLoS One.

[218] Sato C (2018). Tau kinetics in neurons and the human central nervous system. Neuron.

[219] Hanisch K (2010). Analysis of human tau in cerebrospinal fluid. J Proteome Res.

[220] Avila J (2016). Tau Structures. Front Aging Neurosci.

[221] Mukrasch MD (2005). Sites of tau important for aggregation populate {beta}-structure and bind to microtubules and polyanions. J Biol Chem.

[222] von Bergen M (2000). Assembly of tau protein into Alzheimer paired helical filaments depends on a local sequence motif ((306)VQIVYK(311)) forming beta structure. Proc Natl Acad Sci U S A.

[223] Schoch KM (2016). Increased 4R-tau induces pathological changes in a human-tau mouse model. Neuron.

[224] Hernandez-Vega A (2017). Local nucleation of microtubule bundles through tubulin concentration into a condensed tau phase. Cell Rep.

[225] Ambadipudi S (2017). Liquid-liquid phase separation of the microtubule-binding repeats of the Alzheimer-related protein tau. Nat Commun.

[226] Wegmann S, et al. Tau protein liquid-liquid phase separation can initiate tau aggregation. EMBO J. 2018:37(7):e98049. 10.15252/embj.201798049 PMC5881631 29472250

[227] Ferreon JC, et al. Acetylation disfavors Tau phase separation. Int J Mol Sci. 2018;19(5):1360. 10.3390/ijms19051360 PMC5983838 29734651

[228] Kahlson MA, Colodner KJ (2015). Glial tau pathology in Tauopathies: functional consequences. J Exp Neurosci.

[229] Hyman BT (1984). Alzheimer’s disease: cell-specific pathology isolates the hippocampal formation. Science.

[230] Braak H, Braak E (1991). Neuropathological stageing of Alzheimer-related changes. Acta Neuropathol.

[231] Braak H (2011). Stages of the pathologic process in Alzheimer disease: age categories from 1 to 100 years. J Neuropathol Exp Neurol.

[232] Clavaguera F (2009). Transmission and spreading of tauopathy in transgenic mouse brain. Nat Cell Biol.

[233] Iba M (2013). Synthetic tau fibrils mediate transmission of neurofibrillary tangles in a transgenic mouse model of Alzheimer’s-like tauopathy. J Neurosci.

[234] Sanders DW (2014). Distinct tau prion strains propagate in cells and mice and define different tauopathies. Neuron.

[235] Narasimhan S (2017). Pathological tau strains from human brains recapitulate the diversity of Tauopathies in nontransgenic mouse brain. J Neurosci.

[236] de Calignon A (2012). Propagation of tau pathology in a model of early Alzheimer’s disease. Neuron.

[237] Liu L (2012). Trans-synaptic spread of tau pathology in vivo. PLoS One.

[238] Frost B, Jacks RL, Diamond MI (2009). Propagation of tau misfolding from the outside to the inside of a cell. J Biol Chem.

[239] Santa-Maria I (2012). Paired helical filaments from Alzheimer disease brain induce intracellular accumulation of tau protein in aggresomes. J Biol Chem.

[240] Michel CH (2014). Extracellular monomeric tau protein is sufficient to initiate the spread of tau protein pathology. J Biol Chem.

[241] Abounit S (2016). Tunneling nanotubes: A possible highway in the spreading of tau and other prion-like proteins in neurodegenerative diseases. Prion.

[242] Tardivel M (2016). Tunneling nanotube (TNT)-mediated neuron-to neuron transfer of pathological tau protein assemblies. Acta Neuropathol Commun.

[243] Kim W (2010). Interneuronal transfer of human tau between lamprey central neurons in situ. J Alzheimers Dis.

[244] Kim W, Lee S, Hall GF (2010). Secretion of human tau fragments resembling CSF-tau in Alzheimer’s disease is modulated by the presence of the exon 2 insert. FEBS Lett.

[245] Yamada K (2014). Neuronal activity regulates extracellular tau in vivo. J Exp Med.

[246] Yamada K (2011). In vivo microdialysis reveals age-dependent decrease of brain interstitial fluid tau levels in P301S human tau transgenic mice. J Neurosci.

[247] Yamada K (2015). Analysis of in vivo turnover of tau in a mouse model of tauopathy. Mol Neurodegener.

[248] Saman S (2012). Exosome-associated tau is secreted in tauopathy models and is selectively phosphorylated in cerebrospinal fluid in early Alzheimer disease. J Biol Chem.

[249] Edgar JR (2016). Q&A: what are exosomes, exactly?. BMC Biol.

[250] Wang Y (2017). The release and trans-synaptic transmission of tau via exosomes. Mol Neurodegener.

[251] Asai H (2015). Depletion of microglia and inhibition of exosome synthesis halt tau propagation. Nat Neurosci.

[252] Jia L (2019). Concordance between the assessment of Abeta42, T-tau, and P-T181-tau in peripheral blood neuronal-derived exosomes and cerebrospinal fluid. Alzheimers Dement.

[253] Fiandaca MS (2015). Identification of preclinical Alzheimer’s disease by a profile of pathogenic proteins in neurally derived blood exosomes: A case-control study. Alzheimers Dement.

[254] Guix FX, et al. Detection of aggregation-competent Tau in neuron-derived extracellular vesicles. Int J Mol Sci. 2018;19(3):663. 10.3390/ijms19030663 PMC5877524 29495441

[255] Sokolow S (2015). Pre-synaptic C-terminal truncated tau is released from cortical synapses in Alzheimer’s disease. J Neurochem.

[256] Zhou L (2017). Tau association with synaptic vesicles causes presynaptic dysfunction. Nat Commun.

[257] Pooler AM (2013). Physiological release of endogenous tau is stimulated by neuronal activity. EMBO Rep.

[258] Wu JW (2016). Neuronal activity enhances tau propagation and tau pathology in vivo. Nat Neurosci.

[259] Merezhko M (2018). Secretion of tau via an unconventional non-vesicular mechanism. Cell Rep.

[260] Katsinelos T (2018). Unconventional secretion mediates the trans-cellular spreading of tau. Cell Rep.

[261] Wu JW (2013). Small misfolded tau species are internalized via bulk endocytosis and anterogradely and retrogradely transported in neurons. J Biol Chem.

[262] Holmes BB (2013). Heparan sulfate proteoglycans mediate internalization and propagation of specific proteopathic seeds. Proc Natl Acad Sci U S A.

[263] Bolos M (2017). Absence of CX3CR1 impairs the internalization of tau by microglia. Mol Neurodegener.

[264] Evans LD (2018). Extracellular monomeric and aggregated tau efficiently enter human neurons through overlapping but distinct pathways. Cell Rep.

[265] Rauch JN (2018). Tau internalization is regulated by 6-O Sulfation on Heparan sulfate proteoglycans (HSPGs). Sci Rep.

[266] Kaufman SK (2017). Characterization of tau prion seeding activity and strains from formaldehyde-fixed tissue. Acta Neuropathol Commun.

[267] Kaufman SK (2016). Tau prion strains dictate patterns of cell pathology, progression rate, and regional vulnerability in vivo. Neuron.

[268] Kopeikina KJ, Hyman BT, Spires-Jones TL (2012). Soluble forms of tau are toxic in Alzheimer’s disease. Transl Neurosci.

[269] Ward SM (2012). Tau oligomers and tau toxicity in neurodegenerative disease. Biochem Soc Trans.

[270] Tracy TE, Gan L (2018). Tau-mediated synaptic and neuronal dysfunction in neurodegenerative disease. Curr Opin Neurobiol.

[271] Akwa Y (2018). Synaptic activity protects against AD and FTD-like pathology via autophagic-lysosomal degradation. Mol Psychiatry.

[272] Wang ZX, Tan L, Yu JT (2015). Axonal transport defects in Alzheimer’s disease. Mol Neurobiol.

[273] Eckert A (2014). March separate, strike together--role of phosphorylated TAU in mitochondrial dysfunction in Alzheimer’s disease. Biochim Biophys Acta.

[274] Kopeikina KJ (2011). Tau accumulation causes mitochondrial distribution deficits in neurons in a mouse model of tauopathy and in human Alzheimer’s disease brain. Am J Pathol.

[275] Cheng Y, Bai F (2018). The Association of tau with Mitochondrial Dysfunction in Alzheimer’s disease. Front Neurosci.

[276] Manczak M, Reddy PH (2012). Abnormal interaction between the mitochondrial fission protein Drp1 and hyperphosphorylated tau in Alzheimers disease neurons: implications for mitochondrial dysfunction and neuronal damage. Hum Mol Genet.

[277] Kandimalla R (2016). Reduced dynamin-related protein 1 protects against phosphorylated tau-induced mitochondrial dysfunction and synaptic damage in Alzheimer’s disease. Hum Mol Genet.

[278] Santacruz K (2005). Tau suppression in a neurodegenerative mouse model improves memory function. Science.

[279] Kaufman AC (2015). Fyn inhibition rescues established memory and synapse loss in Alzheimer mice. Ann Neurol.

[280] Tintori C (2015). Studies on the ATP binding site of Fyn kinase for the identification of new inhibitors and their evaluation as potential agents against Tauopathies and tumors. J Med Chem.

[281] McInnes J (2018). Synaptogyrin-3 mediates presynaptic dysfunction induced by tau. Neuron.

[282] Frade JM, Ovejero-Benito MC (2015). Neuronal cell cycle: the neuron itself and its circumstances. Cell Cycle.

[283] Vincent I (1997). Aberrant expression of mitotic cdc2/cyclin B1 kinase in degenerating neurons of Alzheimer’s disease brain. J Neurosci.

[284] Hradek AC (2015). Distinct chronology of neuronal cell cycle re-entry and tau pathology in the 3xTg-AD mouse model and Alzheimer’s disease patients. J Alzheimers Dis.

[285] Levin OS, Vasenina EE (2016). Twenty-five years of the amyloid hypothesis of alzheimer disease: advances, failures and new perspectives. Zh Nevrol Psikhiatr Im S S Korsakova.

[286] Tanzi RE. The genetics of Alzheimer disease. Cold Spring Harb Perspect Med. 2012;2(10):a006296. 10.1101/cshperspect.a006296 PMC3475404 23028126

[287] Hutton M (1998). Association of missense and 5′-splice-site mutations in tau with the inherited dementia FTDP-17. Nature.

[288] Brunden KR, Trojanowski JQ, Lee VM (2009). Advances in tau-focused drug discovery for Alzheimer’s disease and related tauopathies. Nat Rev Drug Discov.

[289] Karran E, Hardy J (2014). A critique of the drug discovery and phase 3 clinical programs targeting the amyloid hypothesis for Alzheimer disease. Ann Neurol.

[290] Holcomb L (1998). Accelerated Alzheimer-type phenotype in transgenic mice carrying both mutant amyloid precursor protein and presenilin 1 transgenes. Nat Med.

[291] Duyckaerts C, Potier MC, Delatour B (2008). Alzheimer disease models and human neuropathology: similarities and differences. Acta Neuropathol.

[292] Echeverria V (2004). Altered mitogen-activated protein kinase signaling, tau hyperphosphorylation and mild spatial learning dysfunction in transgenic rats expressing the beta-amyloid peptide intracellularly in hippocampal and cortical neurons. Neuroscience.

[293] Cohen RM (2013). A transgenic Alzheimer rat with plaques, tau pathology, behavioral impairment, oligomeric abeta, and frank neuronal loss. J Neurosci.

[294] Vossel KA (2015). Tau reduction prevents Abeta-induced axonal transport deficits by blocking activation of GSK3beta. J Cell Biol.

[295] Terrill-Usery SE, Mohan MJ, Nichols MR (2014). Amyloid-beta(1-42) protofibrils stimulate a quantum of secreted IL-1beta despite significant intracellular IL-1beta accumulation in microglia. Biochim Biophys Acta.

[296] Gratuze M, Leyns CEG, Holtzman DM (2018). New insights into the role of TREM2 in Alzheimer’s disease. Mol Neurodegener.

[297] Kitazawa M (2011). Blocking IL-1 signaling rescues cognition, attenuates tau pathology, and restores neuronal beta-catenin pathway function in an Alzheimer’s disease model. J Immunol.

[298] Matousek SB (2012). Chronic IL-1beta-mediated neuroinflammation mitigates amyloid pathology in a mouse model of Alzheimer’s disease without inducing overt neurodegeneration. J NeuroImmune Pharmacol.

[299] Bloom GS (2014). Amyloid-beta and tau: the trigger and bullet in Alzheimer disease pathogenesis. JAMA Neurol.

[300] Rapoport M (2002). Tau is essential to beta -amyloid-induced neurotoxicity. Proc Natl Acad Sci U S A.

[301] Roberson ED (2007). Reducing endogenous tau ameliorates amyloid beta-induced deficits in an Alzheimer’s disease mouse model. Science.

[302] Leroy K (2012). Lack of tau proteins rescues neuronal cell death and decreases amyloidogenic processing of APP in APP/PS1 mice. Am J Pathol.

[303] Castillo-Carranza DL (2015). Tau immunotherapy modulates both pathological tau and upstream amyloid pathology in an Alzheimer’s disease mouse model. J Neurosci.

[304] Zhang Z, et al. delta-Secretase-cleaved Tau stimulates Abeta production via upregulating STAT1-BACE1 signaling in Alzheimer’s disease. Mol Psychiatry. 2018. 10.1038/s41380-018-0286-z. 10.1038/s41380-018-0286-z PMC6684859 30382187

[305] Oddo S (2006). Temporal profile of amyloid-beta (Abeta) oligomerization in an in vivo model of Alzheimer disease. A link between Abeta and tau pathology. J Biol Chem.

[306] Miao Y (2010). Deletion of tau attenuates heat shock-induced injury in cultured cortical neurons. J Neurosci Res.

[307] Miller Y, Ma B, Nussinov R (2011). Synergistic interactions between repeats in tau protein and Abeta amyloids may be responsible for accelerated aggregation via polymorphic states. Biochemistry.

[308] Guo JP (2006). Abeta and tau form soluble complexes that may promote self aggregation of both into the insoluble forms observed in Alzheimer’s disease. Proc Natl Acad Sci U S A.

[309] Iijima K, Gatt A, Iijima-Ando K (2010). Tau Ser262 phosphorylation is critical for Abeta42-induced tau toxicity in a transgenic Drosophila model of Alzheimer’s disease. Hum Mol Genet.

[310] Fein JA (2008). Co-localization of amyloid beta and tau pathology in Alzheimer’s disease synaptosomes. Am J Pathol.

[311] Jin X, Yamashita T (2016). Microglia in central nervous system repair after injury. J Biochem.

[312] Imai F (2007). Neuroprotective effect of exogenous microglia in global brain ischemia. J Cereb Blood Flow Metab.

[313] Molina-Holgado E (2000). Induction of COX-2 and PGE(2) biosynthesis by IL-1beta is mediated by PKC and mitogen-activated protein kinases in murine astrocytes. Br J Pharmacol.

[314] Li R (2004). Tumor necrosis factor death receptor signaling cascade is required for amyloid-beta protein-induced neuron death. J Neurosci.

[315] Pan XD (2011). Microglial phagocytosis induced by fibrillar beta-amyloid is attenuated by oligomeric beta-amyloid: implications for Alzheimer’s disease. Mol Neurodegener.

[316] Deczkowska A (2018). Disease-associated microglia: A universal immune sensor of neurodegeneration. Cell.

[317] Keren-Shaul H (2017). A unique microglia type associated with restricting development of Alzheimer’s disease. Cell.

[318] Liu CC (2013). Apolipoprotein E and Alzheimer disease: risk, mechanisms and therapy (vol 9, pg 106, 2013). Nat Rev Neurol.

[319] Baker M (2006). Mutations in progranulin cause tau-negative frontotemporal dementia linked to chromosome 17. Nature.

[320] Paloneva J (2000). Loss-of-function mutations in TYROBP (DAP12) result in a presenile dementia with bone cysts. Nat Genet.

[321] Rangaraju S (2018). Identification and therapeutic modulation of a pro-inflammatory subset of disease-associated-microglia in Alzheimer’s disease. Mol Neurodegener.

[322] Song WM (2018). Humanized TREM2 mice reveal microglia-intrinsic and -extrinsic effects of R47H polymorphism. J Exp Med.

[323] Wang Y (2015). TREM2 lipid sensing sustains the microglial response in an Alzheimer’s disease model. Cell.

[324] Lee CYD (2018). Elevated TREM2 gene dosage reprograms microglia responsivity and ameliorates pathological phenotypes in Alzheimer’s disease models. Neuron.

[325] Griciuc A (2013). Alzheimer’s disease risk gene CD33 inhibits microglial uptake of amyloid beta. Neuron.

[326] Sofroniew MV (2015). Astrocyte barriers to neurotoxic inflammation. Nat Rev Neurosci.

[327] Serrano-Pozo A (2011). Reactive glia not only associates with plaques but also parallels tangles in Alzheimer’s disease. Am J Pathol.

[328] Thal DR (2012). The role of astrocytes in amyloid beta-protein toxicity and clearance. Exp Neurol.

[329] Mathur R (2015). A reduced astrocyte response to beta-amyloid plaques in the ageing brain associates with cognitive impairment. PLoS One.

[330] Cregg JM (2014). Functional regeneration beyond the glial scar. Exp Neurol.

[331] Parpura V (2012). Glial cells in (patho)physiology. J Neurochem.

[332] Scimemi A (2013). Amyloid-beta1-42 slows clearance of synaptically released glutamate by mislocalizing astrocytic GLT-1. J Neurosci.

[333] Liddelow SA (2017). Neurotoxic reactive astrocytes are induced by activated microglia. Nature.

[334] Joshi AU (2019). Fragmented mitochondria released from microglia trigger A1 astrocytic response and propagate inflammatory neurodegeneration. Nat Neurosci.

[335] Schmid CD (2002). Heterogeneous expression of the triggering receptor expressed on myeloid cells-2 on adult murine microglia. J Neurochem.

[336] Thrash JC, Torbett BE, Carson MJ (2009). Developmental regulation of TREM2 and DAP12 expression in the murine CNS: implications for Nasu-Hakola disease. Neurochem Res.

[337] Xing J, Titus AR, Humphrey MB (2015). The TREM2-DAP12 signaling pathway in Nasu-Hakola disease: a molecular genetics perspective. Res Rep Biochem.

[338] Peng Q (2010). TREM2- and DAP12-dependent activation of PI3K requires DAP10 and is inhibited by SHIP1. Sci Signal.

[339] Korvatska O (2015). R47H variant of TREM2 associated with Alzheimer disease in a large late-onset family: clinical, genetic, and neuropathological study. JAMA Neurol.

[340] Lill CM (2015). The role of TREM2 R47H as a risk factor for Alzheimer’s disease, frontotemporal lobar degeneration, amyotrophic lateral sclerosis, and Parkinson's disease. Alzheimers Dement.

[341] Zhao Y (2018). TREM2 is a receptor for beta-amyloid that mediates microglial function. Neuron.

[342] Zhong L (2018). Amyloid-beta modulates microglial responses by binding to the triggering receptor expressed on myeloid cells 2 (TREM2). Mol Neurodegener.

[343] Yuan P (2016). TREM2 Haplodeficiency in mice and humans impairs the microglia barrier function leading to decreased amyloid compaction and severe axonal dystrophy. Neuron.

[344] Xiang X (2018). The Trem2 R47H Alzheimer’s risk variant impairs splicing and reduces Trem2 mRNA and protein in mice but not in humans. Mol Neurodegener.

[345] Del-Aguila JL (2019). TREM2 brain transcript-specific studies in AD and TREM2 mutation carriers. Mol Neurodegener.

[346] Bemiller SM, et al. TREM2 deficiency exacerbates tau pathology through dysregulated kinase signaling in a mouse model of tauopathy. Mol Neurodegener. 2017;12(1):74. 10.1186/s13024-017-0216-6 PMC5644120 29037207

[347] Leyns CEG (2017). TREM2 deficiency attenuates neuroinflammation and protects against neurodegeneration in a mouse model of tauopathy. Proc Natl Acad Sci U S A.

[348] Leyns CEG (2019). TREM2 function impedes tau seeding in neuritic plaques. Nat Neurosci.

[349] Deane R, Wu Z, Zlokovic BV (2004). RAGE (yin) versus LRP (yang) balance regulates alzheimer amyloid beta-peptide clearance through transport across the blood-brain barrier. Stroke.

[350] Beisiegel U (1989). The LDL-receptor-related protein, LRP, is an apolipoprotein E-binding protein. Nature.

[351] Myklebost O (1989). The gene for the human putative apoE receptor is on chromosome 12 in the segment q13-14. Genomics.

[352] Kowal RC (1989). Low density lipoprotein receptor-related protein mediates uptake of cholesteryl esters derived from apoprotein E-enriched lipoproteins. Proc Natl Acad Sci U S A.

[353] Herz J (1988). Surface location and high affinity for calcium of a 500-kd liver membrane protein closely related to the LDL-receptor suggest a physiological role as lipoprotein receptor. EMBO J.

[354] May P (2004). Neuronal LRP1 functionally associates with postsynaptic proteins and is required for normal motor function in mice. Mol Cell Biol.

[355] Liu CC (2017). Astrocytic LRP1 mediates brain Abeta clearance and impacts amyloid deposition. J Neurosci.

[356] Wyss-Coray T (2003). Adult mouse astrocytes degrade amyloid-beta in vitro and in situ. Nat Med.

[357] Yang L (2016). LRP1 modulates the microglial immune response via regulation of JNK and NF-kappaB signaling pathways. J Neuroinflammation.

[358] Marzolo MP (2000). Expression of alpha(2)-macroglobulin receptor/low density lipoprotein receptor-related protein (LRP) in rat microglial cells. J Neurosci Res.

[359] Shinohara M (2017). Role of LRP1 in the pathogenesis of Alzheimer’s disease: evidence from clinical and preclinical studies. J Lipid Res.

[360] Kanekiyo T (2013). Neuronal clearance of amyloid-beta by endocytic receptor LRP1. J Neurosci.

[361] Chuang TY (2016). LRP1 expression in microglia is protective during CNS autoimmunity. Acta Neuropathol Commun.

[362] Kielian T (2006). Toll-like receptors in central nervous system glial inflammation and homeostasis. J Neurosci Res.

[363] Olson JK, Miller SD (2004). Microglia initiate central nervous system innate and adaptive immune responses through multiple TLRs. J Immunol.

[364] Fuller JP, Stavenhagen JB, Teeling JL. New roles for fc receptors in neurodegeneration-the impact on lmmunotherapy for Alzheimer’s disease. Front Neurosci. 2014;8:235. 10.3389/fnins.2014.00235 PMC4139653 25191216

[365] Husemann J (2001). Scavenger receptor class B type I (SR-BI) mediates adhesion of neonatal murine microglia to fibrillar beta-amyloid. J Neuroimmunol.

[366] Moore KJ (2002). A CD36-initiated signaling cascade mediates inflammatory effects of beta-amyloid. J Biol Chem.

[367] Lue LF (2001). Involvement of microglial receptor for advanced glycation endproducts (RAGE) in Alzheimer’s disease: identification of a cellular activation mechanism. Exp Neurol.

[368] Liu S (2012). TLR2 is a primary receptor for Alzheimer’s amyloid beta peptide to trigger neuroinflammatory activation. J Immunol.

[369] Landreth GE, Reed-Geaghan EG (2009). Toll-like receptors in Alzheimer’s disease. Curr Top Microbiol Immunol.

[370] Fassbender K (2004). The LPS receptor (CD14) links innate immunity with Alzheimer’s disease. FASEB J.

[371] Song M (2011). TLR4 mutation reduces microglial activation, increases Abeta deposits and exacerbates cognitive deficits in a mouse model of Alzheimer’s disease. J Neuroinflammation.

[372] Veerhuis R (2003). Amyloid beta plaque-associated proteins C1q and SAP enhance the Abeta1-42 peptide-induced cytokine secretion by adult human microglia in vitro. Acta Neuropathol.

[373] Hong S (2016). Complement and microglia mediate early synapse loss in Alzheimer mouse models. Science.

[374] El Khoury JB (2003). CD36 mediates the innate host response to beta-amyloid. J Exp Med.

[375] Fang F (2010). RAGE-dependent signaling in microglia contributes to neuroinflammation, Abeta accumulation, and impaired learning/memory in a mouse model of Alzheimer’s disease. FASEB J.

[376] Chapuis J (2013). Increased expression of BIN1 mediates Alzheimer genetic risk by modulating tau pathology. Mol Psychiatry.

[377] Kam TI (2013). FcgammaRIIb mediates amyloid-beta neurotoxicity and memory impairment in Alzheimer’s disease. J Clin Invest.

[378] Lambert JC (2013). Meta-analysis of 74,046 individuals identifies 11 new susceptibility loci for Alzheimer’s disease. Nat Genet.

[379] Miyashita A (2013). SORL1 is genetically associated with late-onset Alzheimer’s disease in Japanese, Koreans and Caucasians. PLoS One.

[380] Talantova M (2013). Abeta induces astrocytic glutamate release, extrasynaptic NMDA receptor activation, and synaptic loss. Proc Natl Acad Sci U S A.

[381] Yang KC, Jin GZ, Wu J (2009). Mysterious alpha6-containing nAChRs: function, pharmacology, and pathophysiology. Acta Pharmacol Sin.

[382] Velez-Fort M, Audinat E, Angulo MC (2009). Functional alpha 7-containing nicotinic receptors of NG2-expressing cells in the hippocampus. Glia.

[383] Takarada T (2012). Possible neuroprotective property of nicotinic acetylcholine receptors in association with predominant upregulation of glial cell line-derived neurotrophic factor in astrocytes. J Neurosci Res.

[384] Jonnala RR, Buccafusco JJ (2001). Relationship between the increased cell surface alpha7 nicotinic receptor expression and neuroprotection induced by several nicotinic receptor agonists. J Neurosci Res.

[385] Gray R (1996). Hippocampal synaptic transmission enhanced by low concentrations of nicotine. Nature.

[386] Ji D, Lape R, Dani JA (2001). Timing and location of nicotinic activity enhances or depresses hippocampal synaptic plasticity. Neuron.

[387] Dani JA (2001). Nicotinic receptor activity alters synaptic plasticity. ScientificWorldJournal.

[388] Pirttimaki TM (2013). alpha7 Nicotinic receptor-mediated astrocytic gliotransmitter release: Abeta effects in a preclinical Alzheimer’s mouse model. PLoS One.

[389] Brown EM, MacLeod RJ (2001). Extracellular calcium sensing and extracellular calcium signaling. Physiol Rev.

[390] Hendy GN, Guarnieri V, Canaff L (2009). Calcium-sensing receptor and associated diseases. Prog Mol Biol Transl Sci.

[391] Msaouel P (2004). Extracellular calcium sensing receptor: an overview of physiology, pathophysiology and clinical perspectives. In Vivo.

[392] Hofer AM, Brown EM (2003). Extracellular calcium sensing and signalling. Nat Rev Mol Cell Biol.

[393] Yano S, Brown EM, Chattopadhyay N (2004). Calcium-sensing receptor in the brain. Cell Calcium.

[394] Dal Pra I (2005). Roles of Ca2+ and the Ca2+−sensing receptor (CASR) in the expression of inducible NOS (nitric oxide synthase)-2 and its BH4 (tetrahydrobiopterin)-dependent activation in cytokine-stimulated adult human astrocytes. J Cell Biochem.

[395] Chattopadhyay N (2000). Regulation of secretion of PTHrP by ca(2+)-sensing receptor in human astrocytes, astrocytomas, and meningiomas. Am J Physiol Cell Physiol.

[396] Riccardi D, Kemp PJ (2012). The Calcium-Sensing Receptor Beyond Extracellular Calcium Homeostasis: Conception, Development, Adult Physiology, and Disease. Annu Rev Physiol.

[397] Ruat M, Traiffort E (2013). Roles of the calcium sensing receptor in the central nervous system. Best Pract Res Clin Endocrinol Metab.

[398] Bandyopadhyay S, Tfelt-Hansen J, Chattopadhyay N (2010). Diverse roles of extracellular calcium-sensing receptor in the central nervous system. J Neurosci Res.

[399] Bandyopadhyay S (2007). Calcium-sensing receptor stimulates secretion of an interferon-gamma-induced monokine (CXCL10) and monocyte chemoattractant protein-3 in immortalized GnRH neurons. J Neurosci Res.

[400] Chiarini A (2009). Calcium-sensing receptor (CaSR) in human Brain's pathophysiology: roles in late-onset Alzheimer’s disease (LOAD). Curr Pharm Biotechnol.

[401] Chiarini A (2016). Calcium-sensing receptors of human neural cells play crucial roles in Alzheimer’s disease. Front Physiol.

[402] Armato U (2013). Calcium-sensing receptor antagonist (calcilytic) NPS 2143 specifically blocks the increased secretion of endogenous Abeta42 prompted by exogenous fibrillary or soluble Abeta25-35 in human cortical astrocytes and neurons-therapeutic relevance to Alzheimer’s disease. Biochim Biophys Acta.

[403] Zhang X (2019). Potential astrocytic receptors and transporters in the pathogenesis of Alzheimer’s disease. J Alzheimers Dis.

[404] Bao Y (2012). CD36 is involved in astrocyte activation and astroglial scar formation. J Cereb Blood Flow Metab.

[405] Sick E (2011). Activation of CD47 receptors causes proliferation of human astrocytoma but not normal astrocytes via an Akt-dependent pathway. Glia.

[406] Askarova S (2011). Role of Abeta-receptor for advanced glycation endproducts interaction in oxidative stress and cytosolic phospholipase A(2) activation in astrocytes and cerebral endothelial cells. Neuroscience.

[407] Jones RS, AMM, Connor TJ, Lynch MA (2013). Amyloid-β-induced astrocytic phagocytosis is mediated by CD36, CD47 and RAGE. J NeuroImmune Pharmacol.

[408] Gonzalez-Reyes RE, Rubiano MG (2018). Astrocyte s RAGE: more than just a question of mood. Cent Nerv Syst Agents Med Chem.

[409] Choi BR (2014). Increased expression of the receptor for advanced glycation end products in neurons and astrocytes in a triple transgenic mouse model of Alzheimer’s disease. Exp Mol Med.

[410] Iliff JJ (2012). A paravascular pathway facilitates CSF flow through the brain parenchyma and the clearance of interstitial solutes, including amyloid beta. Sci Transl Med.

[411] Albargothy NJ (2018). Convective influx/glymphatic system: tracers injected into the CSF enter and leave the brain along separate periarterial basement membrane pathways. Acta Neuropathol.

[412] Louveau A (2015). Structural and functional features of central nervous system lymphatic vessels. Nature.

[413] Plog BA, Nedergaard M (2018). The Glymphatic system in central nervous system health and disease: past, present, and future. Annu Rev Pathol.

[414] Schubert JJ (2019). Dynamic (11)C-PiB PET shows cerebrospinal fluid flow alterations in Alzheimer disease and multiple sclerosis. J Nucl Med.

[415] Peng W (2016). Suppression of glymphatic fluid transport in a mouse model of Alzheimer’s disease. Neurobiol Dis.

[416] Hughes TM (2013). Pulse wave velocity is associated with beta-amyloid deposition in the brains of very elderly adults. Neurology.

[417] Zeppenfeld DM (2017). Association of Perivascular Localization of Aquaporin-4 with cognition and Alzheimer disease in aging brains. JAMA Neurol.

[418] Xu Z (2015). Deletion of aquaporin-4 in APP/PS1 mice exacerbates brain Abeta accumulation and memory deficits. Mol Neurodegener.

[419] Xie L (2013). Sleep drives metabolite clearance from the adult brain. Science.

[420] Holth JK (2019). The sleep-wake cycle regulates brain interstitial fluid tau in mice and CSF tau in humans. Science.

[421] Wang L (2013). Expression of Tau40 induces activation of cultured rat microglial cells. PLoS One.

[422] Ma L (2017). Proinflammatory effects of S100A8/A9 via TLR4 and RAGE signaling pathways in BV-2 microglial cells. Int J Mol Med.

[423] Wang WY (2015). Role of pro-inflammatory cytokines released from microglia in Alzheimer’s disease. Ann Transl Med.

[424] Wang H (2018). Genome-wide RNAseq study of the molecular mechanisms underlying microglia activation in response to pathological tau perturbation in the rTg4510 tau transgenic animal model. Mol Neurodegener.

[425] Stancu IC (2019). Aggregated tau activates NLRP3-ASC inflammasome exacerbating exogenously seeded and non-exogenously seeded tau pathology in vivo. Acta Neuropathol.

[426] Halle A (2008). The NALP3 inflammasome is involved in the innate immune response to amyloid-beta. Nat Immunol.

[427] Heneka MT (2013). NLRP3 is activated in Alzheimer’s disease and contributes to pathology in APP/PS1 mice. Nature.

[428] Luo W (2015). Microglial internalization and degradation of pathological tau is enhanced by an anti-tau monoclonal antibody. Sci Rep.

[429] Maphis N (2015). Reactive microglia drive tau pathology and contribute to the spreading of pathological tau in the brain. Brain.

[430] Laurent C, Buee L, Blum D (2018). Tau and neuroinflammation: what impact for Alzheimer’s disease and Tauopathies?. Biom J.

[431] Kitazawa M (2005). Lipopolysaccharide-induced inflammation exacerbates tau pathology by a cyclin-dependent kinase 5-mediated pathway in a transgenic model of Alzheimer’s disease. J Neurosci.

[432] Ikeda M (2005). Accumulation of filamentous tau in the cerebral cortex of human tau R406W transgenic mice. Am J Pathol.

[433] Ikeda K (1995). Thorn-shaped astrocytes: possibly secondarily induced tau-positive glial fibrillary tangles. Acta Neuropathol.

[434] Forman MS (2005). Transgenic mouse model of tau pathology in astrocytes leading to nervous system degeneration. J Neurosci.

[435] Martini-Stoica H (2018). TFEB enhances astroglial uptake of extracellular tau species and reduces tau spreading. J Exp Med.

[436] Ghoshal N (2001). Tau-66: evidence for a novel tau conformation in Alzheimer’s disease. J Neurochem.

[437] Barres BA (2008). The mystery and magic of glia: a perspective on their roles in health and disease. Neuron.

[438] Pita-Almenar JD (2012). Relationship between increase in astrocytic GLT-1 glutamate transport and late-LTP. Learn Mem.

[439] Maragakis NJ, Rothstein JD (2001). Glutamate transporters in neurologic disease. Arch Neurol.

[440] Gatz M (2006). Role of genes and environments for explaining Alzheimer disease. Arch Gen Psychiatry.

[441] Malik M (2015). Genetics ignite focus on microglial inflammation in Alzheimer’s disease. Mol Neurodegener.

[442] Zhang ZG (2015). Inflammation in Alzheimer’s disease and molecular genetics: recent update. Arch Immunol Ther Exp.

[443] Harold D (2009). Genome-wide association study identifies variants at CLU and PICALM associated with Alzheimer’s disease. Nat Genet.

[444] Naj AC (2011). Common variants at MS4A4/MS4A6E, CD2AP, CD33 and EPHA1 are associated with late-onset Alzheimer’s disease. Nat Genet.

[445] Hollingworth P (2011). Common variants at ABCA7, MS4A6A/MS4A4E, EPHA1, CD33 and CD2AP are associated with Alzheimer’s disease. Nat Genet.

[446] Elshourbagy NA (1985). Apolipoprotein E mRNA is abundant in the brain and adrenals, as well as in the liver, and is present in other peripheral tissues of rats and marmosets. Proc Natl Acad Sci U S A.

[447] Hatters DM, Peters-Libeu CA, Weisgraber KH (2006). Apolipoprotein E structure: insights into function. Trends Biochem Sci.

[448] Fernandez CG (2019). The role of APOE4 in disrupting the homeostatic functions of astrocytes and microglia in aging and Alzheimer’s disease. Front Aging Neurosci.

[449] Coon KD (2007). A high-density whole-genome association study reveals that APOE is the major susceptibility gene for sporadic late-onset Alzheimer’s disease. J Clin Psychiatry.

[450] Phillips MC (2014). Apolipoprotein E isoforms and lipoprotein metabolism. IUBMB Life.

[451] Liu CC (2013). Apolipoprotein E and Alzheimer disease: risk, mechanisms and therapy. Nat Rev Neurol.

[452] Corder EH (1993). Gene dose of apolipoprotein E type 4 allele and the risk of Alzheimer’s disease in late onset families. Science.

[453] Farrer LA (1997). Effects of age, sex, and ethnicity on the association between apolipoprotein E genotype and Alzheimer disease. A meta-analysis. APOE and Alzheimer disease Meta analysis consortium. JAMA.

[454] Verghese PB, Castellano JM, Holtzman DM (2011). Apolipoprotein E in Alzheimer’s disease and other neurological disorders. Lancet Neurol.

[455] Tai LM (2011). Introducing human APOE into Abeta transgenic mouse models. Int J Alzheimers Dis.

[456] Liu CC (2017). ApoE4 accelerates early seeding of amyloid pathology. Neuron.

[457] Zhao W (2014). Human APOE genotype affects intraneuronal Abeta1-42 accumulation in a lentiviral gene transfer model. Hum Mol Genet.

[458] Dodart JC (2005). Gene delivery of human apolipoprotein E alters brain Abeta burden in a mouse model of Alzheimer’s disease. Proc Natl Acad Sci U S A.

[459] Kok E (2009). Apolipoprotein E-dependent accumulation of Alzheimer disease-related lesions begins in middle age. Ann Neurol.

[460] Prince JA (2004). APOE epsilon4 allele is associated with reduced cerebrospinal fluid levels of Abeta42. Neurology.

[461] Head D (2012). Exercise engagement as a moderator of the effects of APOE genotype on amyloid deposition. Arch Neurol.

[462] Vemuri P (2010). Effect of apolipoprotein E on biomarkers of amyloid load and neuronal pathology in Alzheimer disease. Ann Neurol.

[463] Espeseth T (2012). Apolipoprotein E epsilon4-related thickening of the cerebral cortex modulates selective attention. Neurobiol Aging.

[464] Reiman EM (1998). Hippocampal volumes in cognitively normal persons at genetic risk for Alzheimer’s disease. Ann Neurol.

[465] Lin YT (2018). APOE4 causes widespread molecular and cellular alterations associated with Alzheimer’s disease phenotypes in human iPSC-derived brain cell types. Neuron.

[466] Bell RD (2012). Apolipoprotein E controls cerebrovascular integrity via cyclophilin A. Nature.

[467] Arboleda-Velasquez JF, et al. Resistance to autosomal dominant Alzheimer’s disease in an APOE3 Christchurch homozygote: a case report. Nat Med. 2019;25(11):1680–3. 10.1038/s41591-019-0611-3 PMC6898984 31686034

[468] Yamazaki Y (2019). Apolipoprotein E and Alzheimer disease: pathobiology and targeting strategies. Nat Rev Neurol.

[469] Shi Y (2017). ApoE4 markedly exacerbates tau-mediated neurodegeneration in a mouse model of tauopathy. Nature.

[470] Crary JF (2014). Primary age-related tauopathy (PART): a common pathology associated with human aging. Acta Neuropathol.

[471] Zhao N, et al. APOE epsilon 2 is associated with increased tau pathology in primary tauopathy. Nat Commun. 2018;9(1):4388. 10.1038/s41467-018-06783-0 PMC6197187 30348994

[472] Ikeda K (1997). A subset of senile dementia with high incidence of the apolipoprotein E epsilon2 allele. Ann Neurol.

[473] Olah M (2018). A transcriptomic atlas of aged human microglia. Nat Commun.

[474] Reitz C (2011). Meta-analysis of the association between variants in SORL1 and Alzheimer disease. Arch Neurol.

[475] Bertram L (2007). Systematic meta-analyses of Alzheimer disease genetic association studies: the AlzGene database. Nat Genet.

[476] De Rossi P (2016). Predominant expression of Alzheimer’s disease-associated BIN1 in mature oligodendrocytes and localization to white matter tracts. Mol Neurodegener.

[477] Karch CM (2012). Expression of novel Alzheimer’s disease risk genes in control and Alzheimer’s disease brains. PLoS One.

[478] Sartori M (2019). BIN1 recovers tauopathy-induced long-term memory deficits in mice and interacts with tau through Thr(348) phosphorylation. Acta Neuropathol.

[479] Calafate S (2016). Loss of Bin1 promotes the propagation of tau pathology. Cell Rep.

[480] Biffi A (2010). Genetic variation and neuroimaging measures in Alzheimer disease. Arch Neurol.

[481] Franzmeier N (2019). The BIN1 rs744373 SNP is associated with increased tau-PET levels and impaired memory. Nat Commun.

[482] Miyashita T (2012). Mg(2+) block of Drosophila NMDA receptors is required for long-term memory formation and CREB-dependent gene expression. Neuron.

[483] Vardarajan BN (2015). Coding mutations in SORL1 and Alzheimer disease. Ann Neurol.

[484] Yin RH, Yu JT, Tan L (2015). The role of SORL1 in Alzheimer’s disease. Mol Neurobiol.

[485] Rogaeva E (2007). The neuronal sortilin-related receptor SORL1 is genetically associated with Alzheimer disease. Nat Genet.

[486] Andersen OM (2005). Neuronal sorting protein-related receptor sorLA/LR11 regulates processing of the amyloid precursor protein. Proc Natl Acad Sci U S A.

[487] Dodson SE (2008). Loss of LR11/SORLA enhances early pathology in a mouse model of amyloidosis: evidence for a proximal role in Alzheimer’s disease. J Neurosci.

[488] Hu T (2017). Real-time analysis of binding events between different Abeta1-42 species and human Lilrb2 by dual polarization interferometry. Anal Chem.

[489] Klemsz MJ (1990). The macrophage and B cell-specific transcription factor PU.1 is related to the ets oncogene. Cell.

[490] Walton MR (2000). PU.1 expression in microglia. J Neuroimmunol.

[491] Ponomarev ED (2011). MicroRNA-124 promotes microglia quiescence and suppresses EAE by deactivating macrophages via the C/EBP-alpha-PU.1 pathway. Nat Med.

[492] Huang KL (2017). A common haplotype lowers PU.1 expression in myeloid cells and delays onset of Alzheimer’s disease. Nat Neurosci.

[493] Rustenhoven J (2018). PU.1 regulates Alzheimer’s disease-associated genes in primary human microglia. Mol Neurodegener.

[494] Assoc As (2018). 2018 Alzheimer’s disease facts and figures (vol 14, pg 367, 2018). Alzheimers Dement.

[495] Carmona JJ, Michan S (2016). Biology of healthy aging and longevity. Rev Investig Clin.

[496] Bradley-Whitman MA (2014). Nucleic acid oxidation: an early feature of Alzheimer’s disease. J Neurochem.

[497] Lovell MA, Markesbery WR (2007). Oxidative DNA damage in mild cognitive impairment and late-stage Alzheimer’s disease. Nucleic Acids Res.

[498] Lovell MA, Soman S, Bradley MA (2011). Oxidatively modified nucleic acids in preclinical Alzheimer’s disease (PCAD) brain. Mech Ageing Dev.

[499] Mecocci P (1998). Oxidative damage to DNA in lymphocytes from AD patients. Neurology.

[500] Migliore L (2005). Oxidative DNA damage in peripheral leukocytes of mild cognitive impairment and AD patients. Neurobiol Aging.

[501] Weissman L (2007). Defective DNA base excision repair in brain from individuals with Alzheimer’s disease and amnestic mild cognitive impairment. Nucleic Acids Res.

[502] Hou Y (2018). NAD(+) supplementation normalizes key Alzheimer’s features and DNA damage responses in a new AD mouse model with introduced DNA repair deficiency. Proc Natl Acad Sci U S A.

[503] Grodstein F (2008). Shorter telomeres may mark early risk of dementia: preliminary analysis of 62 participants from the nurses’ health study. PLoS One.

[504] Majores M (2000). Allelic association between the D10S1423 marker and Alzheimer’s disease in a German population. Neurosci Lett.

[505] D'Introno A (2006). Current knowledge of chromosome 12 susceptibility genes for late-onset Alzheimer’s disease. Neurobiol Aging.

[506] Lopez-Otin C (2013). The hallmarks of aging. Cell.

[507] Wang HZ (2016). Validating GWAS-identified risk loci for Alzheimer’s disease in Han Chinese populations. Mol Neurobiol.

[508] Graff J (2012). An epigenetic blockade of cognitive functions in the neurodegenerating brain. Nature.

[509] Guan JS (2009). HDAC2 negatively regulates memory formation and synaptic plasticity. Nature.

[510] Horvath S (2013). DNA methylation age of human tissues and cell types. Genome Biol.

[511] Unnikrishnan A (2018). Revisiting the genomic hypomethylation hypothesis of aging. Ann N Y Acad Sci.

[512] Li P (2019). Epigenetic dysregulation of enhancers in neurons is associated with Alzheimer’s disease pathology and cognitive symptoms. Nat Commun.

[513] Bove R (2014). Age at surgical menopause influences cognitive decline and Alzheimer pathology in older women. Neurology.

[514] De Jager PL (2014). Alzheimer’s disease: early alterations in brain DNA methylation at ANK1, BIN1, RHBDF2 and other loci. Nat Neurosci.

[515] Gibson GE, Shi Q (2010). A mitocentric view of Alzheimer’s disease suggests multi-faceted treatments. J Alzheimers Dis.

[516] Shen Y (2018). Cognitive Decline, Dementia, Alzheimer’s Disease and Presbycusis: Examination of the Possible Molecular Mechanism. Front Neurosci.

[517] Swerdlow RH, Burns JM, Khan SM (2014). The Alzheimer’s disease mitochondrial cascade hypothesis: progress and perspectives. Biochim Biophys Acta.

[518] Kerr JS (2017). Mitophagy and Alzheimer’s disease: cellular and molecular mechanisms. Trends Neurosci.

[519] Fang EF (2019). Mitophagy inhibits amyloid-beta and tau pathology and reverses cognitive deficits in models of Alzheimer’s disease. Nat Neurosci.

[520] Villeda SA (2014). Young blood reverses age-related impairments in cognitive function and synaptic plasticity in mice. Nat Med.

[521] Middeldorp J (2016). Preclinical assessment of young blood plasma for Alzheimer disease. JAMA Neurol.

[522] Castellano JM (2017). Human umbilical cord plasma proteins revitalize hippocampal function in aged mice. Nature.

[523] Galatro TF (2017). Transcriptomic analysis of purified human cortical microglia reveals age-associated changes. Nat Neurosci.

[524] Daria A (2017). Young microglia restore amyloid plaque clearance of aged microglia. EMBO J.

[525] Clarke LE (2018). Normal aging induces A1-like astrocyte reactivity. Proc Natl Acad Sci U S A.

[526] Sjogren T, Sjogren H, Lindgren AG (1952). Morbus Alzheimer and morbus pick; a genetic, clinical and patho-anatomical study. Acta Psychiatr Neurol Scand Suppl.

[527] Middleton PJ (1980). Herpes-simplex viral genome and senile and presenile dementias of Alzheimer and pick. Lancet.

[528] Itzhaki RF (2014). Herpes simplex virus type 1 and Alzheimer’s disease: increasing evidence for a major role of the virus. Front Aging Neurosci.

[529] Readhead B (2018). Multiscale analysis of independent Alzheimer’s cohorts finds disruption of molecular, genetic, and clinical networks by human herpesvirus. Neuron.

[530] Li H (2018). Amyloid, tau, pathogen infection and antimicrobial protection in Alzheimer’s disease -conformist, nonconformist, and realistic prospects for AD pathogenesis. Transl Neurodegener.

[531] Singhrao SK (2015). Porphyromonas gingivalis periodontal infection and its putative links with Alzheimer’s disease. Mediat Inflamm.

[532] Steel AJ, Eslick GD (2015). Herpes viruses increase the risk of Alzheimer’s disease: A Meta-analysis. J Alzheimers Dis.

[533] Itzhaki RF (2018). Corroboration of a major role for herpes simplex virus type 1 in Alzheimer’s disease. Front Aging Neurosci.

[534] De Chiara G (2019). Recurrent herpes simplex virus-1 infection induces hallmarks of neurodegeneration and cognitive deficits in mice. PLoS Pathog.

[535] Wang Y, et al. Roles of HSV-1 infection-induced microglial immune responses in CNS diseases: friends or foes? Crit Rev Microbiol. 2019;45(5–6):581–94. 10.1080/1040841X.2019.1660615 31512533

[536] Eimer WA (2018). Alzheimer’s Disease-Associated β-Amyloid Is Rapidly Seeded by Herpesviridae to Protect against Brain Infection. Neuron.

[537] Dominy SS (2019). Porphyromonas gingivalis in Alzheimer’s disease brains: Evidence for disease causation and treatment with small-molecule inhibitors. Sci Adv.

[538] Liu Y (2019). Metal ions in Alzheimer’s disease: A key role or not?. Acc Chem Res.

[539] Zatta P (2009). Alzheimer’s disease, metal ions and metal homeostatic therapy. Trends Pharmacol Sci.

[540] Zhao Y (2012). CutA divalent cation tolerance homolog (Escherichia coli) (CUTA) regulates beta-cleavage of beta-amyloid precursor protein (APP) through interacting with beta-site APP cleaving protein 1 (BACE1). J Biol Chem.

[541] Hambright WS (2017). Ablation of ferroptosis regulator glutathione peroxidase 4 in forebrain neurons promotes cognitive impairment and neurodegeneration. Redox Biol.

[542] Ma Q (2006). Copper binding properties of a tau peptide associated with Alzheimer’s disease studied by CD, NMR, and MALDI-TOF MS. Peptides.

[543] Martic S, Rains MK, Kraatz HB (2013). Probing copper/tau protein interactions electrochemically. Anal Biochem.

[544] Kitazawa M, Cheng D, Laferla FM (2009). Chronic copper exposure exacerbates both amyloid and tau pathology and selectively dysregulates cdk5 in a mouse model of AD. J Neurochem.

[545] Voss K (2014). Modulation of tau phosphorylation by environmental copper. Transl Neurodegener.

[546] Doll S (2017). ACSL4 dictates ferroptosis sensitivity by shaping cellular lipid composition. Nat Chem Biol.

[547] Shah R, Shchepinov MS, Pratt DA (2018). Resolving the role of lipoxygenases in the initiation and execution of Ferroptosis. ACS Cent Sci.

[548] Watt NT, Whitehouse IJ, Hooper NM (2010). The role of zinc in Alzheimer’s disease. Int J Alzheimers Dis.

[549] Andrasi E (2005). Brain aluminum, magnesium and phosphorus contents of control and Alzheimer-diseased patients. J Alzheimers Dis.

[550] Cilliler AE, Ozturk S, Ozbakir S (2007). Serum magnesium level and clinical deterioration in Alzheimer’s disease. Gerontology.

[551] Li W (2014). Elevation of brain magnesium prevents synaptic loss and reverses cognitive deficits in Alzheimer’s disease mouse model. Mol Brain.

[552] Wilson RS (2011). Vulnerability to stress, anxiety, and development of dementia in old age. Am J Geriatr Psychiatry.

[553] Donovan NJ (2018). Longitudinal Association of Amyloid Beta and Anxious-Depressive Symptoms in cognitively Normal older adults. Am J Psychiatry.

[554] Hayashi T (2015). Conversion of psychological stress into cellular stress response: roles of the sigma-1 receptor in the process. Psychiatry Clin Neurosci.

[555] Miller MW, Sadeh N (2014). Traumatic stress, oxidative stress and post-traumatic stress disorder: neurodegeneration and the accelerated-aging hypothesis. Mol Psychiatry.

[556] Nicolaides NC (2015). Stress, the stress system and the role of glucocorticoids. Neuroimmunomodulation.

[557] Smith SM, Vale WW (2006). The role of the hypothalamic-pituitary-adrenal axis in neuroendocrine responses to stress. Dialogues Clin Neurosci.

[558] Ellis BJ, Del Giudice M (2014). Beyond allostatic load: rethinking the role of stress in regulating human development. Dev Psychopathol.

[559] Sapolsky RM, Krey LC, McEwen BS (1985). Prolonged glucocorticoid exposure reduces hippocampal neuron number: implications for aging. J Neurosci.

[560] Bubu OM, et al. Sleep, cognitive impairment, and alzheimer’s disease: a systematic review and meta-analysis. Sleep. 2017;40(1):10.1093. 10.1093/sleep/zsw032 28364458

[561] Shi L (2018). Sleep disturbances increase the risk of dementia: A systematic review and meta-analysis. Sleep Med Rev.

[562] Lim AS (2013). Sleep fragmentation and the risk of incident Alzheimer’s disease and cognitive decline in older persons. Sleep.

[563] McCurry SM (2000). Treatment of sleep disturbance in Alzheimer’s disease. Sleep Med Rev.

[564] Vitiello MV, Prinz PN (1989). Alzheimer’s disease. Sleep and sleep/wake patterns. Clin Geriatr Med.

[565] Rauchs G (2010). Sleep and episodic memory: a review of the literature in young healthy subjects and potential links between sleep changes and memory impairment observed during aging and Alzheimer’s disease. Rev Neurol (Paris).

[566] Rauchs G (2008). Is there a link between sleep changes and memory in Alzheimer’s disease?. Neuroreport.

[567] Roh JH (2012). Disruption of the sleep-wake cycle and diurnal fluctuation of beta-amyloid in mice with Alzheimer’s disease pathology. Sci Transl Med.

[568] Ooms S (2014). Effect of 1 night of total sleep deprivation on cerebrospinal fluid beta-amyloid 42 in healthy middle-aged men: a randomized clinical trial. JAMA Neurol.

[569] Lucey BP (2018). Effect of sleep on overnight cerebrospinal fluid amyloid beta kinetics. Ann Neurol.

[570] Di Meco A, Joshi YB, Pratico D (2014). Sleep deprivation impairs memory, tau metabolism, and synaptic integrity of a mouse model of Alzheimer’s disease with plaques and tangles. Neurobiol Aging.

[571] Rothman SM (2013). Chronic mild sleep restriction accentuates contextual memory impairments, and accumulations of cortical Abeta and pTau in a mouse model of Alzheimer’s disease. Brain Res.

[572] Clarke G (2013). The microbiome-gut-brain axis during early life regulates the hippocampal serotonergic system in a sex-dependent manner. Mol Psychiatry.

[573] Fultz NE (2019). Coupled electrophysiological, hemodynamic, and cerebrospinal fluid oscillations in human sleep. Science.

[574] Lim MM, Gerstner JR, Holtzman DM (2014). The sleep-wake cycle and Alzheimer’s disease: what do we know?. Neurodegener Dis Manag.

[575] Tilg H, Moschen AR (2014). Microbiota and diabetes: an evolving relationship. Gut.

[576] Ley RE, Peterson DA, Gordon JI (2006). Ecological and evolutionary forces shaping microbial diversity in the human intestine. Cell.

[577] Clark A, Mach N (2016). Exercise-induced stress behavior, gut-microbiota-brain axis and diet: a systematic review for athletes. J Int Soc Sports Nutr.

[578] Gibson GR, Roberfroid MB (1995). Dietary modulation of the human colonic microbiota: introducing the concept of prebiotics. J Nutr.

[579] Yano JM (2015). Indigenous bacteria from the gut microbiota regulate host serotonin biosynthesis. Cell.

[580] Cirrito JR (2011). Serotonin signaling is associated with lower amyloid-beta levels and plaques in transgenic mice and humans. Proc Natl Acad Sci U S A.

[581] Neufeld KM (2011). Reduced anxiety-like behavior and central neurochemical change in germ-free mice. Neurogastroenterol Motil.

[582] Wang T (2015). Lactobacillus fermentum NS9 restores the antibiotic induced physiological and psychological abnormalities in rats. Benef Microbes.

[583] Desbonnet L (2015). Re: gut microbiota depletion from early adolescence in mice: implications for brain and behaviour. Brain Behav Immun.

[584] Mohle L (2016). Ly6C(hi) monocytes provide a link between antibiotic-induced changes in gut microbiota and adult hippocampal neurogenesis. Cell Rep.

[585] Minter MR (2016). Antibiotic-induced perturbations in gut microbial diversity influences neuro-inflammation and amyloidosis in a murine model of Alzheimer’s disease. Sci Rep.

[586] Cox PA, et al. Dietary exposure to an environmental toxin triggers neurofibrillary tangles and amyloid deposits in the brain. Proc Biol Sci. 2016;283(1823):20152397. 10.1098/rspb.2015.2397 PMC4795023 26791617

[587] Brenner SR (2013). Blue-green algae or cyanobacteria in the intestinal micro-flora may produce neurotoxins such as Beta-N-Methylamino-L-alanine (BMAA) which may be related to development of amyotrophic lateral sclerosis, Alzheimer’s disease and Parkinson-dementia-complex in humans and equine motor neuron disease in horses. Med Hypotheses.

[588] Romagnoli N, et al. Plasma concentration rise after the intramuscular administration of high dose medetomidine (0.13 mg/kg) for semen collection in cats. Vet Sci. 2020;7(1):17. 10.3390/vetsci7010017 PMC7157624 32028578

[589] Jiang C (2017). The gut microbiota and Alzheimer’s disease. J Alzheimers Dis.

[590] Zhuang ZQ (2018). Gut microbiota is altered in patients with Alzheimer’s disease. J Alzheimers Dis.

[591] Harach T (2017). Reduction of Abeta amyloid pathology in APPPS1 transgenic mice in the absence of gut microbiota. Sci Rep.

[592] Sun J (2019). Fecal microbiota transplantation alleviated Alzheimer’s disease-like pathogenesis in APP/PS1 transgenic mice. Transl Psychiatry.

[593] Kim MS, et al. Transfer of a healthy microbiota reduces amyloid and tau pathology in an Alzheimer’s disease animal model. Gut. 2020;69(2):283–94. 10.1136/gutjnl-2018-317431 31471351

[594] Alosco ML (2014). Improved memory function two years after bariatric surgery. Obesity (Silver Spring).

[595] Levin-Allerhand JA, Lominska CE, Smith JD (2002). Increased amyloid- levels in APPSWE transgenic mice treated chronically with a physiological high-fat high-cholesterol diet. J Nutr Health Aging.

[596] Puig KL (2012). Amyloid precursor protein and proinflammatory changes are regulated in brain and adipose tissue in a murine model of high fat diet-induced obesity. PLoS One.

[597] Zhang Y (2016). Geniposide attenuates the phosphorylation of tau protein in cellular and insulin-deficient APP/PS1 transgenic mouse model of Alzheimer’s disease. Chem Biol Drug Des.

[598] Fiocco AJ (2012). Sodium intake and physical activity impact cognitive maintenance in older adults: the NuAge Study. Neurobiol Aging.

[599] Kendig MD, Morris MJ (2019). Reviewing the effects of dietary salt on cognition: mechanisms and future directions. Asia Pac J Clin Nutr.

[600] Faraco G (2019). Dietary salt induces cognitive impairment by promoting tau pathology. Ann Neurol.

[601] Yamada M, Naiki H (2012). Cerebral amyloid angiopathy. Prog Mol Biol Transl Sci.

[602] Yamada M (2015). Cerebral amyloid angiopathy: emerging concepts. J Stroke.

[603] Boyle PA (2015). Cerebral amyloid angiopathy and cognitive outcomes in community-based older persons. Neurology.

[604] Iturria-Medina Y (2016). Early role of vascular dysregulation on late-onset Alzheimer’s disease based on multifactorial data-driven analysis. Nat Commun.

[605] Fotiadis P (2016). Cortical atrophy in patients with cerebral amyloid angiopathy: a case-control study. Lancet Neurol.

[606] Tarasoff-Conway JM (2015). Clearance systems in the brain-implications for Alzheimer disease. Nat Rev Neurol.

[607] Wilcock DM, Vitek MP, Colton CA (2009). Vascular amyloid alters astrocytic water and potassium channels in mouse models and humans with Alzheimer’s disease. Neuroscience.

[608] Greenberg SM (2020). Cerebral amyloid angiopathy and Alzheimer disease - one peptide, two pathways. Nat Rev Neurol.

[609] Wilcock DM, Schmitt FA, Head E (2016). Cerebrovascular contributions to aging and Alzheimer’s disease in Down syndrome. Biochim Biophys Acta.

[610] Mann DM (2001). Amyloid angiopathy and variability in amyloid beta deposition is determined by mutation position in presenilin-1-linked Alzheimer’s disease. Am J Pathol.

[611] Marini S (2019). Association of Apolipoprotein E with Intracerebral Hemorrhage Risk by race/ethnicity: A Meta-analysis. JAMA Neurol.

[612] Woo D (2014). Meta-analysis of genome-wide association studies identifies 1q22 as a susceptibility locus for intracerebral hemorrhage. Am J Hum Genet.

[613] Abe M, Bonini NM (2013). MicroRNAs and neurodegeneration: role and impact. Trends Cell Biol.

[614] Long JM (2019). Novel upregulation of amyloid-beta precursor protein (APP) by microRNA-346 via targeting of APP mRNA 5′-untranslated region: implications in Alzheimer’s disease. Mol Psychiatry.

[615] Geng L (2018). Inhibition of miR-128 abates Abeta-mediated cytotoxicity by targeting PPAR-gamma via NF-kappaB inactivation in primary mouse cortical neurons and Neuro2a cells. Yonsei Med J.

[616] Magistri M (2015). Transcriptomics profiling of Alzheimer’s disease reveal neurovascular defects, altered amyloid-beta homeostasis, and deregulated expression of Long noncoding RNAs. J Alzheimers Dis.

[617] Zhang TM (2018). Expression of BC1 impairs spatial learning and memory in Alzheimer’s disease via APP translation. Mol Neurobiol.

[618] Gu C (2018). Long noncoding RNA EBF3-AS promotes neuron apoptosis in Alzheimer’s disease. DNA Cell Biol.

[619] Zhang L (2019). Silencing of Long noncoding RNA SOX21-AS1 relieves neuronal oxidative stress injury in mice with Alzheimer’s disease by upregulating FZD3/5 via the Wnt signaling pathway. Mol Neurobiol.

[620] Dube U (2019). An atlas of cortical circular RNA expression in Alzheimer disease brains demonstrates clinical and pathological associations. Nat Neurosci.

[621] Hansen TB (2013). Natural RNA circles function as efficient microRNA sponges. Nature.

[622] Pogue AI, Lukiw WJ (2018). Up-regulated pro-inflammatory MicroRNAs (miRNAs) in Alzheimer’s disease (AD) and age-related macular degeneration (AMD). Cell Mol Neurobiol.

[623] Zhao Z (2015). Establishment and dysfunction of the blood-brain barrier. Cell.

[624] Zlokovic BV (2011). Neurovascular pathways to neurodegeneration in Alzheimer’s disease and other disorders. Nat Rev Neurosci.

[625] Arvanitakis Z (2016). Relation of cerebral vessel disease to Alzheimer’s disease dementia and cognitive function in elderly people: a cross-sectional study. Lancet Neurol.

[626] Montagne A (2015). Blood-brain barrier breakdown in the aging human hippocampus. Neuron.

[627] Nelson AR (2016). Neurovascular dysfunction and neurodegeneration in dementia and Alzheimer’s disease. Biochim Biophys Acta.

[628] Sweeney MD (2019). Blood-brain barrier: from physiology to disease and Back. Physiol Rev.

[629] Sweeney MD, Sagare AP, Zlokovic BV (2018). Blood-brain barrier breakdown in Alzheimer disease and other neurodegenerative disorders. Nat Rev Neurol.

[630] Riedel WJ (2014). Preventing cognitive decline in preclinical Alzheimer’s disease. Curr Opin Pharmacol.

[631] Caamano-Isorna F (2006). Education and dementia: a meta-analytic study. Neuroepidemiology.

[632] Valenzuela MJ (2008). Brain reserve and the prevention of dementia. Curr Opin Psychiatry.

[633] Zaidi ZF (2010). Gender differences in human brain: A review. The Open Anatomy Journal.

[634] Yiannopoulou KG, et al. Reasons for failed trials of disease-modifying treatments for alzheimer disease and their contribution in recent research. Biomedicines. 2019;7(4):97. 10.3390/biomedicines7040097 PMC6966425 31835422

[635] Chakrabarti S (2015). Metabolic risk factors of sporadic Alzheimer’s disease: implications in the pathology. Pathogenesis and Treatment Aging Dis.

[636] Beckman D, et al. Oligomeric Abeta in the monkey brain impacts synaptic integrity and induces accelerated cortical aging. Proc Natl Acad Sci U S A. 2019;116(52);26239–46. 10.1073/pnas.1902301116 PMC6936351 31871145

[637] Paspalas CD (2018). The aged rhesus macaque manifests Braak stage III/IV Alzheimer’s-like pathology. Alzheimers Dement.

[638] Egan MF (2019). Randomized trial of Verubecestat for prodromal Alzheimer’s disease. N Engl J Med.

[639] Egan MF (2019). Further analyses of the safety of verubecestat in the phase 3 EPOCH trial of mild-to-moderate Alzheimer’s disease. Alzheimers Res Ther.

[640] Henley D (2019). Preliminary results of a trial of Atabecestat in preclinical Alzheimer’s disease. N Engl J Med.

[641] Long JM, Holtzman DM (2019). Alzheimer disease: an update on pathobiology and treatment strategies. Cell.

[642] Hitt BD (2010). BACE1−/− mice exhibit seizure activity that does not correlate with sodium channel level or axonal localization. Mol Neurodegener.

[643] Hu X (2010). BACE1 deficiency causes altered neuronal activity and neurodegeneration. J Neurosci.

[644] Ou-Yang MH, et al. Axonal organization defects in the hippocampus of adult conditional BACE1 knockout mice. Sci Transl Med. 2018;10(459):eaao5620. 10.1126/scitranslmed.aao5620 PMC11017370 30232227

[645] Schneider L (2020). A resurrection of aducanumab for Alzheimer’s disease. Lancet Neurol.

[646] Satlin A (2016). Design of a Bayesian adaptive phase 2 proof-of-concept trial for BAN2401, a putative disease-modifying monoclonal antibody for the treatment of Alzheimer’s disease. Alzheimers Dement (N Y).

[647] Logovinsky V (2016). Safety and tolerability of BAN2401--a clinical study in Alzheimer’s disease with a protofibril selective Abeta antibody. Alzheimers Res Ther.

[648] Gomez-Isla T (1996). Profound loss of layer II entorhinal cortex neurons occurs in very mild Alzheimer’s disease. J Neurosci.

[649] Soeda Y (2019). Methylene blue inhibits formation of tau fibrils but not of granular tau oligomers: A plausible key to understanding failure of a clinical trial for Alzheimer’s disease. J Alzheimers Dis.

[650] Lovestone S (2015). A phase II trial of tideglusib in Alzheimer’s disease. J Alzheimers Dis.

[651] Beurel E, Grieco SF, Jope RS (2015). Glycogen synthase kinase-3 (GSK3): regulation, actions, and diseases. Pharmacol Ther.

[652] Boxer AL (2019). Safety of the tau-directed monoclonal antibody BIIB092 in progressive supranuclear palsy: a randomised, placebo-controlled, multiple ascending dose phase 1b trial. Lancet Neurol.

